# Genome-wide association mapping and genomic prediction of agronomical traits and breeding values in Iranian wheat under rain-fed and well-watered conditions

**DOI:** 10.1186/s12864-022-08968-w

**Published:** 2022-12-15

**Authors:** Ehsan Rabieyan, Mohammad Reza Bihamta, Mohsen Esmaeilzadeh Moghaddam, Valiollah Mohammadi, Hadi Alipour

**Affiliations:** 1 Department of Agronomy and Plant Breeding, Faculty of Agricultural Sciences and Engineering, University of Tehran, Karaj, Iran; 2 Cereal Department, Seed and Plant Improvement Institute, Karaj, Iran; 3 Department of Plant Production and Genetics, Faculty of Agriculture, Urmia University, Urmia, Iran

**Keywords:** Drought stress, Estimated breeding values, GWAS, Genotyping-by-sequencing, Wheat accessions

## Abstract

**Background:**

The markers detected by genome-wide association study (GWAS) make it possible to dissect genetic structure and diversity at many loci. This can enable a wheat breeder to reveal and used genomic loci controlling drought tolerance. This study was focused on determining the population structure of Iranian 208 wheat landraces and 90 cultivars via genotyping-by-sequencing (GBS) and also on detecting marker-trait associations (MTAs) by GWAS and genomic prediction (GS) of wheat agronomic traits for drought-tolerance breeding. GWASs were conducted using both the original phenotypes (pGWAS) and estimated breeding values (eGWAS). The bayesian ridge regression (BRR), genomic best linear unbiased prediction (gBLUP), and ridge regression-best linear unbiased prediction (rrBLUP) approaches were used to estimate breeding values and estimate prediction accuracies in genomic selection.

**Results:**

Population structure analysis using 2,174,975 SNPs revealed four genetically distinct sub-populations from wheat accessions. D-Genome harbored the lowest number of significant marker pairs and the highest linkage disequilibrium (LD), reflecting different evolutionary histories of wheat genomes. From pGWAS, BRR, gBLUP, and rrBLUP, 284, 363, 359 and 295 significant MTAs were found under normal and 195, 365, 362 and 302 under stress conditions, respectively. The gBLUP with the most similarity (80.98 and 71.28% in well-watered and rain-fed environments, correspondingly) with the pGWAS method in the terms of discovered significant SNPs, suggesting the potential of gBLUP in uncovering SNPs. Results from gene ontology revealed that 29 and 30 SNPs in the imputed dataset were located in protein-coding regions for well-watered and rain-fed conditions, respectively. gBLUP model revealed genetic effects better than other models, suggesting a suitable tool for genome selection in wheat.

**Conclusion:**

We illustrate that Iranian landraces of bread wheat contain novel alleles that are adaptive to drought stress environments. gBLUP model can be helpful for fine mapping and cloning of the relevant QTLs and genes, and for carrying out trait introgression and marker-assisted selection in both normal and drought environments in wheat collections.

**Supplementary Information:**

The online version contains supplementary material available at 10.1186/s12864-022-08968-w.

## Background

Wheat (*Triticum aestivum* L., AABBDD, 2n = 6x = 42), as an economically important crop, provides iron, calcium, zinc, vitamin B, starch, fiber, fats, and dietary proteins [1, 2]. Genetic research on this crop has led to its improved productivity. For example, the last decade (2011-2020) witnessed ~ 1% yield increase per annum [3]. However, further improvement is imperative to feed the global population, which will reach over 9 B by 2050 [4]. As the most important detrimental factor, wheat production is restricted by water-limited conditions in most parts of the world. Improvement of crop tolerance to drought stress is one of the essential efforts that can guarantee sustainable yield in wheat fields [2, 4]. Right now, research attempts are focusing on exploring the genetic foundation of drought tolerance traits by using association analysis of agronomic characteristics and genomic regions [5].

The breeding of high-yielding and drought-tolerant wheat varieties continues to be a challenging task, because of large “environment×genotype” interactions and low heritability related to yield as a complicated agronomic property [6]. To overcome this problem, high-throughput methods in phenomics, including digital imaging, and in genomics, including association mapping, have been used to uncover the genetic mechanisms underlying yield and its relative characteristics under drought. The findings obtained from these methodologies had been practical for further enhancement in wheat yield not only in water-restricted environmental conditions but also in drought-stressed environments [3].

The advent of next-generation sequencing technologies has provided an opportunity to evaluate genetic variation and discover new markers through implementing the genotyping-by-sequencing (GBS) approach [7]. From this approach, molecular markers such as single nucleotide polymorphism (SNP) have been successfully adopted to discover the complicated agronomical properties of wheat and also have been well-known as key elements in the genome-wide association study (GWAS) approach [8]. The purpose of this approach is to detect genomic regions that can either be QTL, gene, or marker related to important traits for gene introgression, gene discovery, or marker-assisted breeding [2]. The markers detected by GWAS make it possible to dissect genetic structure and diversity at many loci. This can enable a wheat breeder to reveal and used genomic loci controlling drought tolerance [5].

In addition to trait mean-based GWAS (pGWAS), there is a chance to estimate breeding values by some methods such as BRR (bayesian ridge regression), gBLUP (genomic best linear unbiased prediction), and rrBLUP (ridge regression-best linear unbiased prediction) and use them in association mapping (i.e., eGWAS). There is a lack of certainty on the best algorithm when utilizing a multiple-regression model in genomic selection and GWAS since the structure of the population and the architecture of the trait have a remarkable effect on identifying marker impacts [9]. As a result, it is imperative to compare the findings from the various algorithms when dissecting the genetic basis of a complicated trait in a crop population for the first time. This process ensures the efficient detection of QTLs responsible for controlling a quantitative trait, and better control of the error of type I, which is often higher in association mapping studies [10].

To date, about 800 marker-trait associations (MTA) and quantitative trait loci (QTL) have been discovered for wheat drought tolerance traits, including yield, root, physiological, and agronomic ones by using association mapping (~ 100 MTAs) and bi-parental mapping (~ 700 QTLs). Only 70 loci, however, are known as the major genomic regions explaining more than 20% of phenotype diversity [11]. In the past, association mapping research in drought-stressed wheat has utilized a small number of molecular markers [12–16], which seems inadequate for efficiently exploring diversity in diverse wheat collections.

Genomic prediction (GP) is a powerful tool to boost the efficiency and speed of breeding schedules by reducing time cycles and increasing selection accuracy. This approach provides an opportunity by which a candidate gene can be chosen via genotyping before phenotype determination [17]. Genomic prediction utilizes all genetic markers within a model to train a prediction model, which is consisted of all genetic impacts. The model is applied to a validation set for estimating its accuracy [18]. Several studies have demonstrated high or moderate GP accuracy for quantitative characteristics in barley (*Hordeum vulgare* L.) [19], maize (*Zea mays* L.) [20], rice (*Oryza sativa* L.) [21], oat (*Avena sativa* L.) [22] and wheat (*Triticum aestivum* L.) [17].

This study was aimed at detecting drought tolerance candidate QTLs, genes, or markers linked with agronomical traits by using GWAS in 208 wheat landraces and 90 cultivars grown under normal and drought conditions. In eGWAS, the goal is to identify SNPs related to the correction value of the traits, which are passed on to the next generation. The next purpose of this work was to select the best model for estimating prediction accuracies in genomic selection. To the best of our knowledge, our report is the first study on pGWAS and eGWAS of agronomical characteristics in Iranian wheat landraces under rain-fed and well-watered conditions. The findings from this research will be an interesting source for marker-assisted breeding, genomic selection, introgression of favorable genes into high-yielding cultivars, and improvement of yield-associated characteristics under drought.

## Results

### Phenotypic data summary

**Fig. 1:**
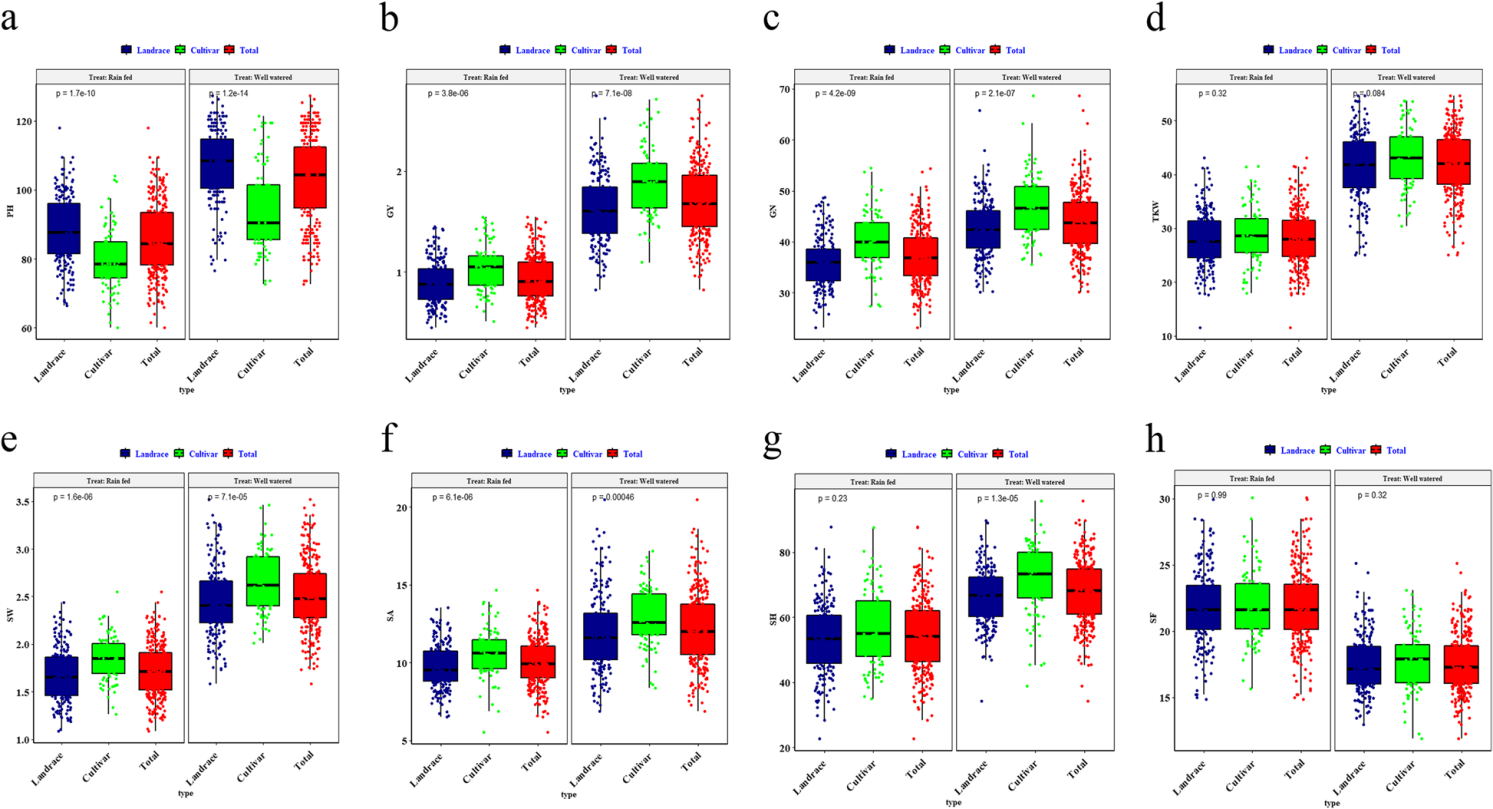
Box-plot representation of the distribution for agronomic traits of Iranian landraces and cultivars in the well-watered and rain-fed environments. Abbreviations: **a** Plant height (cm); **b** Grain yield (g per plant); **c** Grain per spike; **d** Thousand kernel weight (g); **e** Spike weight (g); **f** Spike area (cm^2^); **g** Spike harvest index (%); **h** Spike fertility

In this study, 298 landraces and cultivars of bread wheat were grown under rain-fed and well-watered conditions and analyzed for various agronomic traits. According to the analysis of variance, genotypic, environmental, and genotype×environmental effects on agronomical traits were significant under rain-fed and well-watered environments. Variances associated with genotypic effects were higher than those associated with environment and genotype×environment effects across all traits, indicating genotypic effects had a greater impact. There is a high heritability in plant height traits, but a low heritability in grain yield traits. However, the agronomical traits of wheat grain showed acceptable heritability (Table S1). The box plots related to eight agronomical traits of wheat landraces and cultivars under favorable conditions (well-watered) and drought stress (rain-fed) are shown in Fig. 1. The mean of all traits under stress decreased when compared to a normal situation in both cultivars and native populations implying the presence of considerable diversity in agronomical traits of wheat accessions, and this variation is greater in native populations. The mean of all traits, except plant height, in both conditions, was higher in cultivars than in landraces.

Correlation analysis between traits in the normal environment showed that yield had the highest significant, positive correlation with the following traits, spike harvest index (*r* = 0.72**), spike weight (*r* = 0.71**), 1000-kernel weight (*r* = 0.69**), and the number of grains (*r* = 0.61**). However, in the stress environment, grain yield had the highest significant, positive correlation with the following traits: spike harvest index (*r* = 0.76**), 1000-kernel weight (*r* = 0.74**), the grains per spike (*r* = 0.66**), and spike weight (*r* = 0.54**) (Fig. S1).

### Clustering analysis

**Fig. 2:**
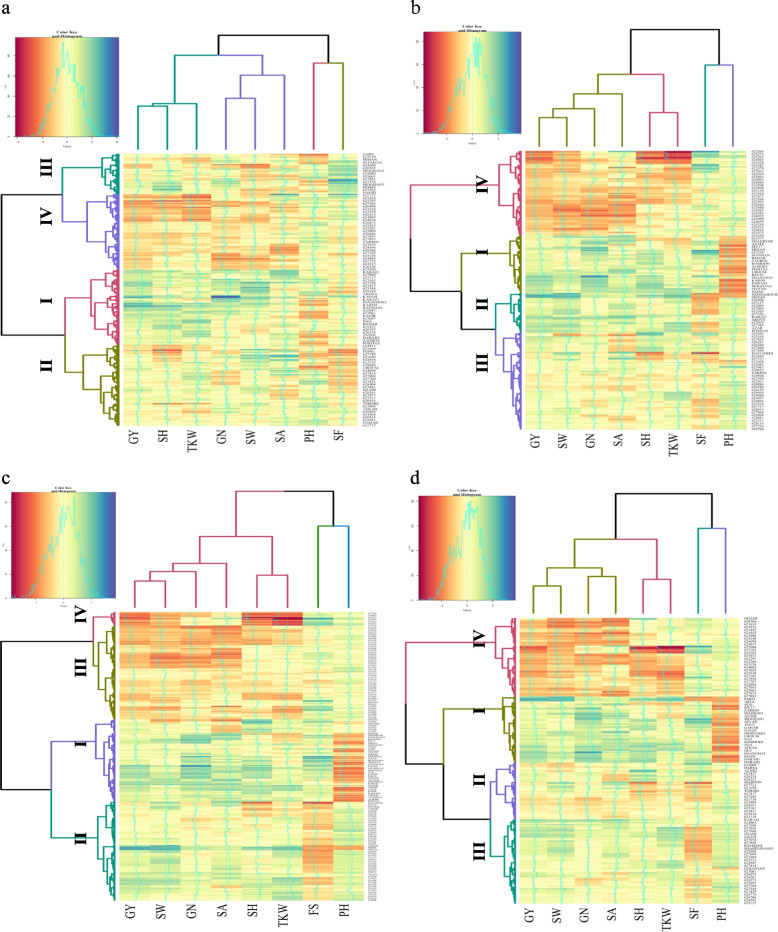
Hierarchical clustering and heatmap of Iranian landraces and cultivars based on the wheat agronomic traits and breeding values in well-watered environments. Agronomic traits (**a**), BRR (**b**), gBLUP (**c**), and rrBLUP (**d**). Abbreviations: PH, Plant height; GY, Grain yield; GN, Grain number per spike; TKW, Thousand kernel weight; SW, Spike weight; SA, Spike area; SH, Spike harvest index (%); SF, Spike fertility

Under normal conditions, the heatmap was plotted based on the mean of agronomic traits and breeding values by using three methods: BRR, gBLUP, and rrBLUP. From the results, wheat accessions were clustered into four groups. In clustering based on the mean of traits, Group No.1 included 82 high-yielding genotypes that were 41 cultivars and 41 landraces, Group No.2 consisted of 89 genotypes with average to high yield (24 cultivars and 65 landraces), Group No.3 contained 44 genotypes with average to low yield (21 cultivars and 23 landraces), and Group No.4 composed of 83 low yielding genotypes that were mainly native populations (4 cultivars and 79 landraces) (Fig. 2a). From the BRR method, wheat genotypes were divided into four groups; the first, second, third, and fourth groups consisted of 61, 42, 104, and 91 genotypes, respectively (Fig. 2b). From the gBLUP, the first group included 85 genotypes with a high breeding value of grain yield (72 cultivars and 13 landraces), the second group consisted of 102 genotypes with medium to high breeding value for yield and yield components (16 cultivars and 86 landraces), the third group contained 97 genotypes with medium to low breeding value for yield and components (2 cultivars and 97 landraces), the fourth group composed of genotypes (17 landraces) with low breeding values for yield and yield components (Fig. 2c). From the BRR method, wheat genotypes were divided into four groups; the first, second, third, and fourth groups consisted of 69 (67 cultivars and 2 landraces), 59 (9 cultivars and 50 landraces), 88 (12 cultivars and 76 landraces), and 82 genotypes (2 cultivars and 88 landraces), respectively (Fig. 2d). The results of gBLUP were most similar to the trait mean method in terms of genotype clustering.

**Fig. 3:**
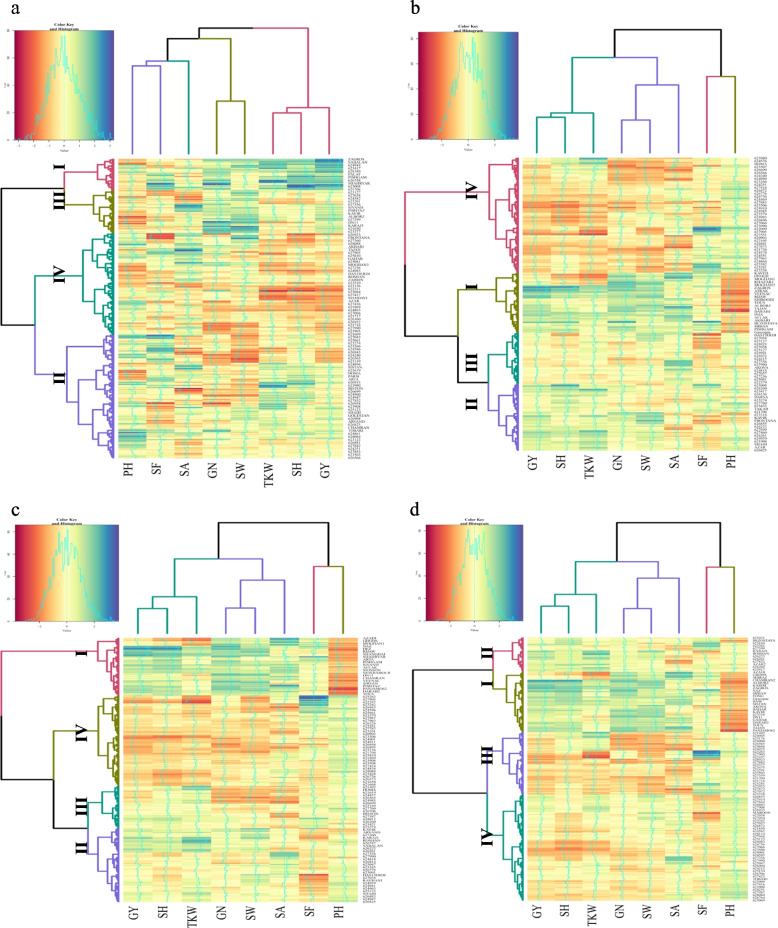
Hierarchical clustering and heatmap of Iranian landraces and cultivars based on the wheat agronomic traits and breeding values in rain-fed environments. Agronomic traits (**a**), BRR (**b**), gBLUP (**c**), and rrBLUP (**d**). Abbreviations: PH, Plant height; GY, Grain yield; GN, Grain number per spike; TKW, Thousand kernel weight; SW, Spike weight; SA, Spike area; SH, Spike harvest index; SF, Spike fertility

Drought-stressed genotypes were also classified into four groups based on the trait mean and the breeding value methods. In clustering based on the mean of traits, the cluster 1 included 31 genotypes with high yield, which were mainly cultivars (18 cultivars and 13 landraces), the cluster 2 consisted of 123 genotypes with average to high yield (24 cultivars and 99 landraces), the cluster 3 contained 43 genotypes with average to low yield (19 cultivars and 24 landraces), and cluster 4 composed of 101 genotypes with low average yield, which were mainly native populations (29 cultivars and 72 landraces) (Fig. 3a). From the BRR, the first group included 61 cultivars with a high breeding value of grain yield, the second group consisted of 67 genotypes (18 cultivars and 49 landraces) with medium to high breeding value for yield and yield components, the third group contained 53 genotypes with medium to low breeding value for yield and components (8 cultivars and 45 landraces), the fourth group composed of 117 genotypes (3 cultivars and 114 landraces) with low breeding values for yield and yield components (Fig. 3b). From the gBLUP method, wheat genotypes were divided into four groups; the first, second, third, and fourth groups consisted of 65, 83, 48, and 102 genotypes, respectively (Fig. 3c). Clustering based on breeding values by using BRR, gBLUP, and rrBLUP had 42, 48, and 39% similarity in terms of genotype clustering in different clusters, respectively. This indicates that the gBLUP categorized wheat accessions more accurately than the other two BRR and rrBLUP methods (Fig. 3b, c and d).

### Linkage Disequilibrium (LD)

LD assessment indicated that this indicator varies between chromosomes and across each chromosome and it usually decreases with increasing distances between SNP locations. A total of 1,858,425 marker pairs with r^2^ = 0.211 were identified in cultivars, of which 700,991 (37.72%) harbored significant linkages at *P* < 0.001. The strongest LD was recorded between marker pairs on chr 4 A (r^2^ = 0.318). Genomes D and B possessed the lowest (63,924) and highest (370,359) number of significant marker pairs, respectively. A similar assessment on wheat landraces found 1,867,575 marker pairs with r^2^ = 0.182, of which 847,725 (45.39%) harbored significant linkages at *P* < 0.01. Similar to cultivars, marker pairs on chr 4 A showed the strongest LD (r^2^ = 0.369). Genomes D and B possessed the lowest and highest number of marker pairs (92,702 and 427,017), respectively. In the D genome, the LD decay was slower than the LD decay in A and B genomes, indicating that the size of the linkage blocks is larger in the D genome. In addition, in cultivars, compared to the native populations in genome D, the LD decay was slower, which probably indicates the selection of more genome-related traits in breeding work. Based on the observations, the most significant marker pairs in wheat landraces were found at distance < 10 cM (Table 1).

**Table 1:** A summary of LD observed among marker pairs and the number of significant marker pairs per genome and chromosome

Chromosome	Total				Landrace				Cultivar			
	**TNSP**	**r** ^**2**^	**Dis. (cM)**	**NSSP**	**TNSP**	**r** ^**2**^	**Dis. (cM)**	**NSSP**	**TNSP**	**r** ^**2**^	**Dis. (cM)**	**NSSP**
1 A	111,575	0.111829	1.333712	49,917 (44.74%)	94,575	0.116906	1.568634	34,895 (36.9%)	85,625	0.148069	1.736676	27,111 (31.66%)
2 A	137,150	0.251605	0.856962	79,772 (58.16%)	125,450	0.289098	0.936772	68,972 (54.98%)	119,450	0.288518	0.972951	57,769 (48.36%)
3 A	96,450	0.130453	2.27878	44,914 (46.57%)	74,950	0.134097	2.933748	28,787 (38.41%)	85,000	0.15728	2.574908	25,912 (30.48%)
4 A	130,500	0.317779	1.378513	79,428 (60.86%)	110,850	0.369392	1.594492	66,016 (59.55%)	116,700	0.36745	1.50704	58,086 (49.77%)
5 A	71,850	0.132927	2.005721	32,488 (45.22%)	60,100	0.146486	2.402626	24,483 (40.74%)	60,600	0.166755	2.38547	18,725 (30.9%)
6 A	99,050	0.158856	1.296073	52,549 (53.05%)	85,850	0.178539	1.498357	40,739 (47.45%)	86,550	0.178744	1.486057	29,651 (34.26%)
7 A	149,700	0.193545	1.164988	78,616 (52.52%)	128,550	0.211862	1.358487	64,114 (49.87%)	129,900	0.232161	1.343972	49,454 (38.07%)
1B	150,800	0.154279	0.932852	80,419 (53.33%)	135,600	0.154625	1.035051	64,442 (47.52%)	132,400	0.20421	1.063407	49,705 (37.54%)
2B	187,300	0.156885	0.764253	102,236 (54.58%)	157,350	0.176011	0.910909	79,057 (50.24%)	166,950	0.19665	0.858127	66,140 (39.62%)
3B	201,700	0.210733	0.771726	119,399 (59.2%)	173,200	0.220043	0.89872	90,266 (52.12%)	177,550	0.243607	0.876084	78,180 (44.03%)
4B	60,050	0.115027	2.20477	23,537 (39.2%)	44,800	0.09777	2.968273	12,423 (27.73%)	52,600	0.142347	2.516753	13,477 (25.62%)
5B	152,400	0.15014	1.292476	80,669 (52.93%)	136,300	0.14202	1.445522	57,252 (42%)	135,650	0.202818	1.431617	55,651 (41.03%)
6B	190,850	0.13708	0.658245	99,314 (52.04%)	167,500	0.135522	0.750676	71,975 (42.97%)	159,700	0.203568	0.787671	66,038 (41.35%)
7B	150,100	0.121987	0.987127	70,107 (46.71%)	127,550	0.12878	1.153868	51,602 (40.46%)	134,150	0.155388	1.102364	41,168 (30.69%)
1D	48,650	0.238268	3.477302	26,009 (53.46%)	42,500	0.226198	3.808863	20,075 (47.24%)	38,350	0.285881	4.409069	16,564 (43.19%)
2D	69,550	0.183692	1.586178	31,547 (45.36%)	55,400	0.163933	1.999469	21,117 (38.12%)	49,600	0.228564	2.23156	16,357 (32.98%)
3D	37,050	0.116765	4.639072	5460 (14.74%)	31,800	0.165445	5.245984	11,619 (36.54%)	26,800	0.137566	6.273779	5458 (20.37%)
4D	13,500	0.122822	9.104484	4560 (33.78%)	11,800	0.130958	10.56137	3577 (30.31%)	11,550	0.154924	10.56621	2312 (20.02%)
5D	31,750	0.130873	6.894582	12,308 (38.77%)	26,250	0.134737	8.311197	9238 (35.19%)	23,700	0.147915	9.317761	5518 (23.28%)
6D	38,300	0.123729	4.134238	15,652 (40.87%)	34,900	0.136001	4.545476	12,619 (36.16%)	29,750	0.137805	5.369092	6852 (23.03%)
7D	46,700	0.150286	4.409549	17,838 (38.2%)	42,300	0.147515	4.882439	14,457 (34.18%)	35,850	0.201644	5.778975	10,863 (30.3%)
A genome	796,275	0.195029	1.397647	417,684 (52.45%)	680,325	0.220024	1.631824	328,006 (48.21%)	683,825	0.232699	1.61945	266,708 (39%)
B genome	1,093,200	0.154972	0.95375	575,681 (52.66%)	942,300	0.1588	1.106081	427,017 (45.32%)	959,000	0.199661	1.084318	370,359 (38.62%)
D genome	285,500	0.162046	4.054108	113,374 (39.71%)	244,950	0.1634	4.684331	92,702 (37.85%)	215,600	0.197637	5.369609	63,924 (29.65%)
Whole genomes	2,174,975	0.170566	1.523235	1,106,739 (50.89%)	1,867,575	0.181706	1.766921	847,725 (45.39%)	1,858,425	0.211583	1.778371	700,991 (37.72%)

Abbreviations: *r*^2^ average squared allele frequency correlation, *TNSP* Total number of SNP pairs, *NSSP* Number of significant SNP pairs (*P* < 0.001), *Dis* Distance

### Population structure

**Fig. 4:**
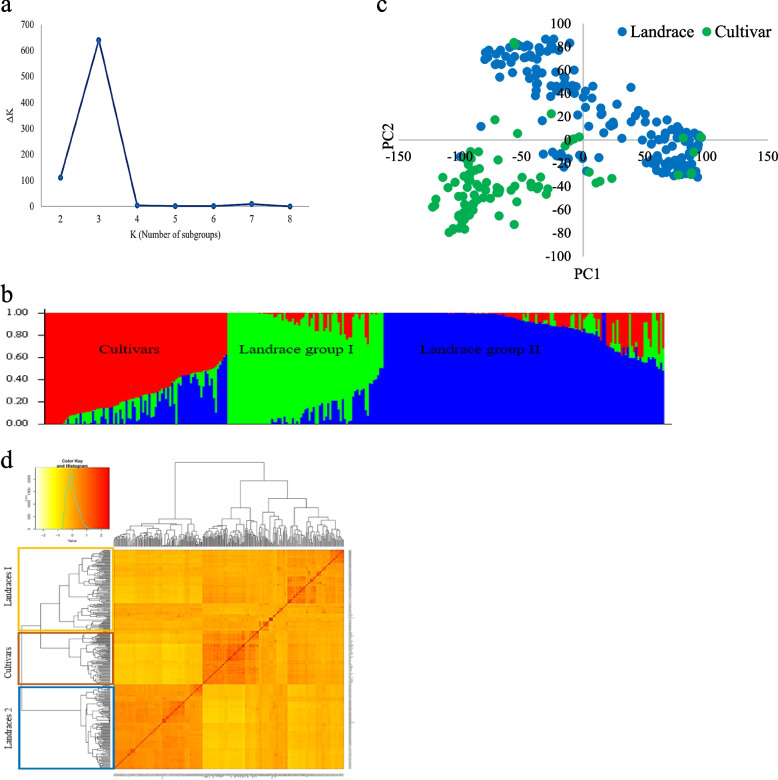
Determination of subpopulations number in wheat genotypes based on ΔK values (**a**), A structure plot of the 298 wheat genotypes and landraces determined by K = 3 (**b**), Principle component analysis (PCA) for a total of 298 Iranian bread wheat accessions (**c**), Cluster analysis using Kinship matrix of imputed data for Iranian wheat accessions (**d**)

To estimate the subpopulations, the ΔK value was plotted against the number of clusters (K). The largest ΔK value was found at K = 3, reflecting three population substructures, Sub.1, Sub.2, and Sub.3 (Fig. 4a). Sub.1 included 113 genotypes with 6 cultivars and 107 landraces; Sub2 contained 111 genotypes with 97 landraces and 14 cultivars; Sub.3 consisted of 74 genotypes with 70 cultivars and 4 landraces (Fig. 4b). From PCA analysis, the estimated PCs showed that PCs 1 and 2 explained 10.29 and 6.28% of the genotypic variation, respectively (Fig. 4c). Cluster analysis using the kinship matrix also supported the STRUCTURE results (Fig. 4d).

### Genome-wide association studies for agronomic traits and estimated breeding values

**Fig. 5:**
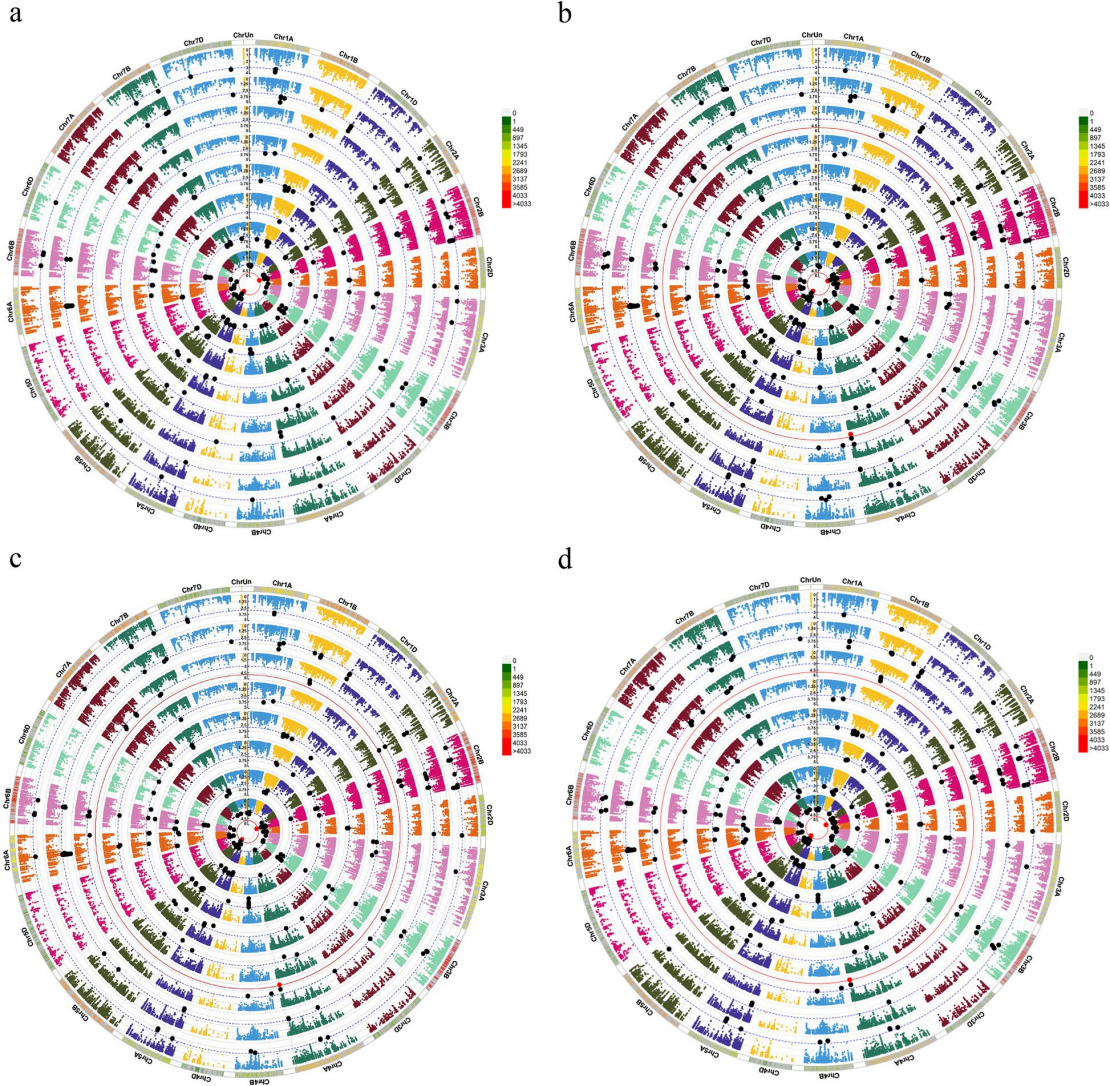
Circular Manhattan plots to draw common regions associated with **a** = Agronomic traits, **b** = BRR, **c** = gBLUP, and **d** = rrBLUP for Iranian wheat landraces and cultivars in well-watered environments. Inner to outer circles represents average trait and breeding values including PH, GY, GN, TKW, SW, SA, SH and SF, respectively. The chromosomes are plotted at the outmost circle where thin-dotted blue and red lines indicate significant level at *P* value < 0.001 (− log10 (p) > 3) and < 0.00001 (− log10 (p) > 5), respectively. Black and red dots indicate genome-wide significantly associated SNPs at *P* value < 0.001 and < 0.00001 probability level, respectively. Scale between ChrUn and Chr1A indicates − log10 (p) values. Colored boxes outside on the top right side indicate SNP density across the genome where green to red indicates less dense to dense. Abbreviations: PH, Plant height; GY, Grain yield; GN, Grain number per spike; TKW, Thousand kernel weight; SW, Spike weight; SA, Spike area; SH, Spike harvest index; SF, Spike fertility

Under optimal irrigation and using imputed markers and -log10 *P* > 3, 283 significant SNPs were discovered for agronomic characteristics by MLM. Of these, 106, 137, and 40 markers were for genomes A, B, and D, respectively. Therefore, genome B had the highest number of significant SNPs. The number of significant markers for PH, GY, GN, TKW, SW, SA, SH, and SF were 39, 57, 19, 48, 11, 31, 43, and 35, respectively (Fig. S2a). The number of significant SNPs based on BRR, gBLUP, and rrBLUP were 362, 358, and 294, respectively. (Fig. S2b, c and d) The gBLUP method with the most similarity (81.27%) in the terms of significant markers had the best justification when compared to other methods (Table 2). BRR, gBLUP, and rrBLUP led to identifying 125, 118, and 111 significant SNPs for genome A; 201, 195, and 147 significant SNPs for genome B; as well as 36, 45, and 36 significant SNPs for genome D, respectively. (Fig. S2b, c and d). The Manhattan Plot results for all original traits are averaged (Fig. 5a) and the correction values of BRR, gBLUP, and rrBLUP (Fig. 5b, c and d) are shown in Fig. 5. The Manhattan circular plot shows significant markers at *P* value < 0.001 (black) and < 0.00001 (red). The Manhattan rectangular and Q-Q plot are shown in Fig. S3. Markers obtained with the mean of agronomic traits were very similar to the results of the breeding value methods, especially gBLUP.

**Table 2:** Similarity of expected MTAs using assigned SNPs for pGWAS and eGWAS

	Well-watered				Rain-fed			
	**pGWAS**	**BRR**	**gBLUP**	**rrBLUP**	**pGWAS**	**BRR**	**gBLUP**	**rrBLUP**
MTA	283	362	358	294	194	364	361	301
Same as pGWAS	-	212	230	195	-	137	139	122
Different with pGWAS	-	151	129	100	-	228	223	180
pGWAS is the same as eGWAS(BRR,gBLUP,rrBLUP)	239	-	-	-	146	-	-	-
Similarity(%)	-	74.91	81.27	68.90	-	70.61	71.64	62.88
GO	12	18	15	16	13	22	20	16
Same as pGWAS	-	8	8	7	-	10	9	8
Different with pGWAS	-	10	7	9	-	12	11	8
pGWAS is the same as eGWAS(BRR,gBLUP,rrBLUP)	8	-	-	-	10	-	-	-
Similarity(%)	-	66.67	66.67	58.33	-	76.92	69.23	61.53

**Fig. 6:**
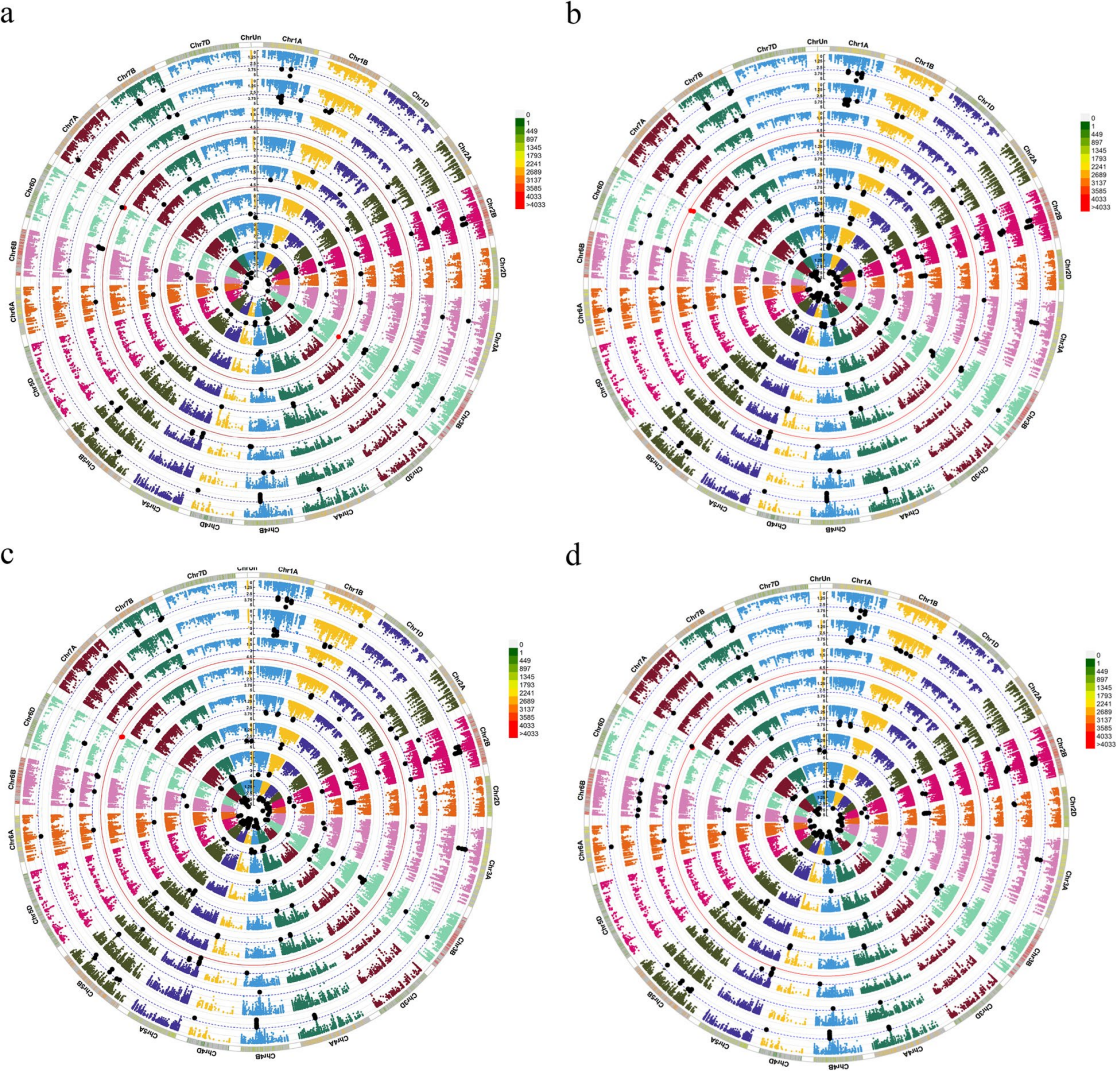
Circular Manhattan plots to draw common regions associated with **a** = Agronomic traits, **b** = BRR, **c** = gBLUP, and **d** = rrBLUP for Iranian wheat landraces and cultivars in rain-fed environments. Inner to outer circles represents average trait and breeding values including PH, GY, GN, TKW, SW, SA, SH and SF, respectively. The chromosomes are plotted at the outmost circle where thin dotted blue and red lines indicate significant level at *P* value < 0.001 (− log10 (p) > 3) and < 0.00001 (− log10 (p) > 5), respectively. Black and red dots indicate genome-wide significantly associated SNPs at *P* value < 0.001 and < 0.00001 probability level, respectively. Scale between ChrUn and Chr1A indicates − log10 (p) values. Colored boxes outside on the top right side indicate SNP density across the genome where green to red indicates less dense to dense. Abbreviations: PH, Plant height; GY, Grain yield; GN, Grain number per spike; TKW, Thousand kernel weight; SW, Spike weight; SA, Spike area; SH, Spike harvest index; SF, Spike fertility

In stress, less significant markers were identified than the normal situation, 194 significant SNPs were identified by the MLM method; Of these, 48, 129, and 17 markers belonged to genomes A, B, and D, respectively. Genome B had the highest percentage of significant SNPs in a stressful environment. The number of significant markers for PH, GY, GN, TKW, SW, SA, SH, and SF were 9, 30, 16, 21, 15, 31, 31, and 41, respectively (Fig. S4a). The number of significant SNPs obtained by BRR, gBLUP, and rrBLUP methods was 364, 361, and 301, respectively (Fig. S4b, c and d). The gBLUP with the most similarity (71.64%) in the terms of significant markers had the best justification when compared to other methods (Table 2). By BRR, gBLUP, and rrBLUP, a total of 134, 121, and 97 significant SNPs for genome A, 187, 198, and 167 SNPs for genome B, as well as 43, 42, and 37 SNPs for genome D were identified, respectively (Fig. S4b, c and d). The Manhattan circular plot shows significant SNPs at *P* value < 0.001 (black) and < 0.00001 (red) (Fig. 6). The Manhattan rectangular and Q-Q plot are shown in Fig. S5.

### Gene ontology

The markers with the highest significance (*P* < 0.0001) and pleiotropic impact were studied in more detail. In the normal environment, 29 markers containing overlapping genes were identified that are involved in important biological and molecular processes. 12 markers were identified based on the pGWAS method and 17 markers were identified based on the eGWAS method. The number of GO based on BRR, gBLUP, and rrBLUP were 18, 15, and 16, respectively. The gBLUP and BRR method was most similar to (66.67%) the pGWAS method. The most significant markers were located on chr 6B, 5B, and 5 A. Of these, 8 SNPs were detected by both pGWAS and eGWAS methods. Some of the uncovered MTAs were responsible for the following molecular and biological processes: lipid biosynthetic process, protein-binding, carbohydrate-binding, lipid transport, RNA-binding, protein ubiquitination, protein deubiquitination, protein catabolic regulation, nucleoside metabolic process, UMP salvage, CTP salvage, and ubiquitin-dependent protein catabolic process (Table 3).

**Table 3:** Description of some expected MTAs using imputed SNPs for agronomic traits of Iranian wheat accessions in well-watered environment

No	SNP	Sequence	Trait- Index	Chromosome	Position (bp)	Analysis method	Biological process	Cellular component	Molecular process
1	rs15519	TGCAGCACTCTGCAAGAAAAACGTCAAAGTAAGAACCACCTACCCACATCTGCTCCAATTCAAA	TKW	1D	47,767	pGWAS	lipid biosynthetic process	integral component of membrane	iron ion binding, oxidoreductase activity
2	rs23792	TGCAGCCCCTGGTCCTCCTGGTGGGAGAGCGTGTGGAACTCAAGGTAGCTGCCGTCCGTCACAA	SA	2 A	11,390	pGWAS	protein binding	-	-
3	rs59777	TGCAGTCTTTCAGAAGTGCAGATGTAAACGTATTGCTATATCAGTGGTTTGAACTACATGGTAA	TKW	2D	58,883	pGWAS, eGWAS(BRR,gBLUP,rrBLUP)	protein ubiquitination, positive regulation of protein catabolic process	Protein binding	-
4	rs61092	TGCAGTGCTGCTAGCGATCAGCTTGGTAGTCTGACAGGAAGGAGAGGCGTATCTACCTATTTAT	GY	3D	54,810	pGWAS	carbohydrate binding	-	-
5	rs23471	TGCAGCCCCATGGCTGGCCACTGCCCCGCCGACGCCACCTGCGGGTTTGGAGACGCCACCACGC	GY	5 A	58,225	pGWAS, eGWAS(BRR,gBLUP,rrBLUP)	RNA binding	-	
6	rs31132	TGCAGCGCTCTCGGCGGTGACTCGTCGTCGCTCGGTGGCATCACCATCAACAAGACACGCGCGC	TKW	5 A	58,225	pGWAS	lipid transport	lipid binding	-
7	rs55428	TGCAGGTTTCAATTACGGAGGGAAAAACTCCAAGAAACTTATTGTTAGCAAGACGAAGTGACTG	GY	5 A	109,694	pGWAS, eGWAS(BRR,gBLUP,rrBLUP)	protein binding	-	-
8	rs31181	TGCAGCGCTGCATCTCTGGATTGTAGCGACAAGGAACTAAGCATGGATTGGAGGTATTATGTAA	PH	5B	26,242	pGWAS, eGWAS(BRR,gBLUP,rrBLUP)	-	-	-
9	rs63031	TGCAGTTCATCAAATTCGAAGGCCTCTCGTGCTAGCATAGCCATCACTTAGTTTGGGAACTGAA	GN	5B	45,594	pGWAS, eGWAS (BRR,gBLUP)	nucleoside metabolic process, UMP salvage, CTP salvage	uridine kinase activity, ATP binding, kinase activity	-
10	rs11075	TGCAGCAAATCGTCACTGCCTTCTAGCACGCCCGCCGTCTCTTAGTTGCAGCACCTAGCCGCCG	SH	6 A	46,797	pGWAS, eGWAS(BRR,gBLUP,rrBLUP)	-	-	-
11	rs44444	TGCAGGAGCTGAGCAACGAGGCCACAGCCGCCGCAGAAAAGGAGTCCCTGAACGGCACACTGGC	TKW	6B	92,187	pGWAS, eGWAS(BRR,gBLUP,rrBLUP)	ubiquitin-dependent protein catabolic process, protein deubiquitination	intracellular anatomical structure	thiol-dependent deubiquitinase
12	rs34582	TGCAGCTACCGCGTGACAAGCTACGTTACGCACGGGGTGCCGCCTCTGGCCGTGGCGGCATGGC	PH	6B	58,062	pGWAS, eGWAS(BRR,gBLUP,rrBLUP)	protein binding	-	-
14	rs44962	TGCAGGAGTCGCCACAGGATGTCTCATTTCTTGCCTTTGCTGGAAGCGTGAATTTCTGCCGATT	SA	1B	45,574	eGWAS(rrBLUP)	DNA topological change	chromosome	DNA binding, DNA topoisomerase activity, DNA topoisomerase type I (single strand cut, ATP-independent) activity
15	rs2390	TGCAGAAGTTCGACCTTTCAACATCTGTCCTGGCAATGCCAGCCATATGAAAAACTTCACATGG	GN	1B	45,574	eGWAS(gBLUP,rrBLUP)	-	anaphase-promoting complex	
16	rs64529	TGCAGTTGTCTATCTCCAGAGAGGCCAGAGACCGTAAACCTCGCAAACAAGTACCCAGCTGCTC	SH	1B	47,847	eGWAS(BRR)	-		ADP binding
17	rs9736	TGCAGATGTGCTTGGCCGTTCGATTGATGCTCTGTTCTTCTTTGCCAGATCCCCAACGGGTGCG	SA	1B	47,279	eGWAS(BRR,gBLUP,rrBLUP)	carbohydrate transport	integral component of membrane	
18	rs28731	TGCAGCGAGCAAGAACATGGCCAAAACGCCGTCGCCGAGATCGGAAGAGCGGGATCACCGACTG	SW	2 A	92,517	eGWAS(rrBLUP)	-	-	electron transfer activity
19	rs3810	TGCAGACCCAAACAAACAGTGTTCAGCCCATGCAAAGCACGAACGTACGTACTAGTATATGCAA	PH	2B	59,184	eGWAS(BRR,gBLUP)	transmembrane transport	membrane, integral component of membrane	transmembrane transporter activity
20	rs49651	TGCAGGGACGGAGACGGAGGTAGGCGGAGGCGTGGTCGGCTTCTTCGCCCTCGTCCTTGGTGGC	SA	2B	71,688	eGWAS(BRR,gBLUP,rrBLUP)	-	-	protein binding
21	rs7932	TGCAGATATTTATCGCCCAAGAGCAAAGATGCTTGACCAGGATTTGGATTGCGGACCGAGATCG	SH	2B	86,479	eGWAS(rrBLUP)	transcription, DNA-templated	-	DNA binding, DNA-directed 5’-3’ RNA polymerase activity, ribonucleoside binding
22	rs41022	TGCAGCTTCTACAGGTCTCTCGTGCTCCATGCATCAAACATGTGGGGACTGGATTCTTGCAGGC	FS	7B	118,551	eGWAS(BRR,rrBLUP)	-	-	ADP binding
23	rs38543	TGCAGCTGCAACCAACACCCTGACGGCGGGCCAGTCGCTCGCCGTCGGCGGCAGCAAGCTCGTC	PH	2D	79,343	eGWAS(BRR)	protein phosphorylation, recognition of pollen	integral component of membrane	protein kinase activity, protein serine/threonine kinase activity, ATP binding
24	rs54935	TGCAGGTTCATTGAGAGAGCGCAGGCTCTGATTCATGGAGATCTCCATACTGGTTCCATCATGT	SH	2D	82,753	eGWAS(BRR,gBLUP)	methionine biosynthetic process, phosphorylation	-	S-methyl-5-thioribose kinase activity
25	rs46842	TGCAGGCCAGCCAAATTTATTGGCACGCGAACGGGAAAACGAACTGTTAAAATATCTGTAACTA	PH	3B	45,525	eGWAS(BRR,gBLUP)	-	-	oxidoreductase activity, oxidoreductase activity, acting on the CH-CH group of donors, NAD or NADP as acceptor, metal ion binding
26	rs24758	TGCAGCCGACGGAGCTCGCGAGCCACATGAGCTCCCGCTGCCCTGCTCTCGAGGACTTGAAACT	PH	3B	121,341	eGWAS(rrBLUP)	-	-	protein binding
27	rs63419	TGCAGTTCGAGCGCCGATGGTGCCTCTTGTTGTGTTGTGTCCCCCCTCGCCATGTGTTGTCCAT	GY	4 A	61,015	eGWAS(rrBLUP)	cation transport, calcium ion transport, transmembrane transport	integral component of membrane	cation transmembrane transporter activity, calcium:proton antiporter activity
28	rs40457	TGCAGCTTAAACATACAAGCAAGCCATACATGCCACGGATGTGGCGCCATTGGTTTACCTTTTA	SH	4 A	146,426	eGWAS(BRR,gBLUP)	-	integral component of membrane	-
29	rs62498	TGCAGTTAATCATTTATTAGTACTAGTTATTAAAAGACCAAGATAGTGAAGACAGAATTCCCTG	SA	4 A	147,563	eGWAS(BRR)	protein phosphorylation	-	protein kinase activity, ATP binding

Abbreviations: *PH* Plant height, *GY* Grain yield, *GN* Grain number per spike, *TKW* Thousand kernel weight, *SW* Spike weight, *SA* Spike area, *SH* Spike harvest index, *SF* Spike fertility

In the stress environment, 30 markers containing overlapping genes were identified. The most significant SNPs were located on the genome B. 13 and 17 markers were identified based on pGWAS and eGWAS methods, respectively. Of these, 10 markers were uncovered by both pGWAS and eGWAS methods, which indicates the approval of the above methods in discovering significant markers. Some of the uncovered MTAs were responsible for the following molecular and biological processes: nucleosome assembly, response to water deprivation, protein-binding, peptidase, monooxygenase, ATP-binding, acyltransferase, oxidoreductase , microtubule-binding, acyltransferase, ADP-binding, methyltransferase activity, metal ion-binding, protein dimerization, serine-type endopeptidase, ATPase, serine-type peptidase, hydrolase, ATP-dependent microtubule motor activity, and heme-binding (Table 4). The following pathways have been discovered using rice reference genomes: metabolic pathways (Fig. S6), oxidative phosphorylation (Fig. S7), biosynthesis of amino acids (Fig. S8), ascorbate and aldarate metabolism (Fig. S9), sulfur metabolism (Fig. S10), and fatty acid elongation (Fig. S11) ([23–25], www.kegg.jp/kegg/kegg1.html).

**Table 4:** Description of some expected MTAs using imputed SNPs for agronomic traits of Iranian wheat accessions in rain-fed environment

No	SNP	Sequence	Trait- Index	Chromosome	Position (bp)	Analysis method	Biological process	Cellular component	Molecular process
1	rs65348	TGCAGTTTTCCGATCGGATATGTCAGCGGCGTCGAGGACCATGCATGGATCGTTTAAAGGTGAT	SH	1 A	44,512	pGWAS, eGWAS(BRR,gBLUP,rrBLUP)	-	-	protein binding
2	rs64529	TGCAGTTGTCTATCTCCAGAGAGGCCAGAGACCGTAAACCTCGCAAACAAGTACCCAGCTGCTC	TKW	1B	47,847	pGWAS, eGWAS(BRR,gBLUP,rrBLUP)	-	-	ADP binding
3	rs51900	TGCAGGGTGGGGGCGGAGAAAAAGGAGGAGGGGCGGCCGAGATCGGAAGAGCGGGATCACCGAC	TKW	2D	28,183	pGWAS, eGWAS(BRR,gBLUP,rrBLUP)	vesicle-mediated transport	plasma membrane, integral component of membrane	protein binding
4	rs56561	TGCAGTACTGGTACCCGCCGCCGCCGTACCAACCGCACCTGTGCCACCTCGCCGAGGAGGACCC	PH	2D	82,753	pGWAS	metal ion transport		metal ion binding
5	rs64448	TGCAGTTGTAATCTTCCATGGAATCCCAACAAGTTTAGAGCGTGTCGATTCGTGGTAGATGGAT	SW	3B	56,892	pGWAS	-	membrane, integral component of membrane	monooxygenase activity, iron ion binding, oxidoreductase activity, acting on paired donors, with incorporation or reduction of molecular oxygen, heme binding
6	rs16023	TGCAGCAGAGGTGGTTTGGAGGTTTGGTGGCGGCAGGATTCCCCTCCCGCGGGCGGCTCGGCTC	GY	3B	56,892	pGWAS	auxin-activated signaling pathway, transmembrane transport, intracellular auxin transport	membrane, integral component of membrane	-
7	rs51991	TGCAGGGTTCGCTCGTCGACGTCAACCCTTTGGAAGCGCAGCTCGAGCGCGGCATCCTTCTGGA	GY	4 A	129,369	pGWAS, eGWAS(BRR,gBLUP,rrBLUP)			ATP binding, ATPase
8	rs38090	TGCAGCTCTGGTTACAGTAGAACGACGAACAAACCTGAACCTGCATCCACACCACCCAGCATTC	TKW	3B	7980	pGWAS, eGWAS(BRR,gBLUP,rrBLUP)	fatty acid biosynthetic process	membrane, integral component of membrane	acyltransferase activity, acyltransferase activity, transferring groups other than amino-acyl groups
9	rs5942	TGCAGAGCATGGTCAGCTTCAGCAGTTCGACAAGCACACGCACCATAGGAGAAAGGTTGCACAT	SA	4B	93,598	pGWAS, eGWAS(BRR)	-	-	methyltransferase activity, protein dimerization activity
10	rs60493	TGCAGTGCAGACGGTATACTTACTCTAGAGTGCAAGCAAAGGAGAAACCGAGGGGAGGAGGAGG	SA	5 A	5684	pGWAS, eGWAS(BRR,gBLUP,rrBLUP)	proteolysis	-	serine-type endopeptidase activity, peptidase activit, serine-type peptidase activity, hydrolase activity
11	rs32859	TGCAGCGGTAGTTCGCTGGCATTGGCATTAGCCAAGGAGCGATGAGCATGGACCCGAGATCGGA	SA	5 A	38,892	pGWAS, eGWAS(BRR,gBLUP,rrBLUP)	-	integral component of membrane	-
12	rs40738	TGCAGCTTCATAGGTCGTACTAGATACTGCAAATACTTTGAAAGCTTAGTTACATGGTTTGTGG	SA	6B	94,461	pGWAS, eGWAS(BRR,gBLUP)	-	integral component of membrane	-
13	rs3733	TGCAGACCAGCACGCCCGCCGCGGCCGCCGTGGTAACGGCGCCGAGATCGGAAGAGCGGGATCA	FS	7B	51,193	pGWAS, eGWAS(BRR,gBLUP,rrBLUP)	microtubule-based movement	-	microtubule motor activity, ATP binding, microtubule binding, ATP-dependent microtubule motor activity
14	rs50966	TGCAGGGGAGGGGCGAGGAAAAGCCTAGCCGCCGAAGCCGTAGAGGGTGCGGCCCTGGCGCTTG	GY	1 A	50,198	eGWAS(rrBLUP)	nucleosome assembly, response to water deprivation	nucleosome, nucleus, chromosome, nucleolus, vacuolar membrane, cytosol, plasma membrane, plasmodesma, chloroplast,thylakoid	DNA binding, protein heterodimerization activity
15	rs60665	TGCAGTGCATTCCTAGCAAGTACTAGGTTAGTTTACTCGTTCAAATACCAAAAGGCAATCTAAG	FS	1 A	66,684	eGWAS(BRR,gBLUP,rrBLUP)	-	-	tRNA binding,GTPase activity,GTP binding
16	rs52091	TGCAGGGTTTGACATTCTGCAAGTACCACCTCAACACCGAGATCGGAAGAGCGGGATCACCGAC	GN	1B	45,574	eGWAS(BRR,gBLUP,rrBLUP)	-	-	protein binding
17	rs14671	TGCAGCACCTTCACGGCAACCATGGAGCCGTCCCGCAGCGTGCCGCGGTACACGCGGCTGTAGC	PH	5 A	3411	eGWAS(BRR,gBLUP)	protein phosphorylation	-	protein kinase activity,ATP binding
18	rs17744	TGCAGCAGGAGCTTGCCGATAAGGTGGCTCTCGACCGAAACGTGGACGAGGCAGACCTCAACAA	PH	1D	9094	eGWAS(BRR)	-	-	monooxygenase activity, iron ion binding,oxidoreductase activity, acting on paired donors, with incorporation or reduction of molecular oxygen,heme binding
19	rs33741	TGCAGCGTGCCTGTGGCTATACGTACTGATCGTTTCCCCGTGTTCCTCCACACGGGCAGGTTCG	PH	2 A	59,228	eGWAS(BRR,gBLUP)	biosynthetic process	-	strictosidine synthase activity
20	rs61008	TGCAGTGCTACTCACACAGGGGAATCAGGCCTGACATTCGCCATCTTCTTCTGCTCAGCCAACC	SW	2B	59,184	eGWAS(BRR,gBLUP,rrBLUP)	-	integral component of membrane	
21	rs21291	TGCAGCCACCTTCGAAATGTGCATCCCCTTTACCCGTATCGGGAGAACGAGGTGTAGCTCAGTT	SH	2B	67,141	eGWAS(BRR)	-	-	ADP binding
22	rs48315	TGCAGGCGGATCTGGTCCAGCAGACGGTCCGCCTCGCCCTCGGCGTCGGCGTCGGCGGCGTCGG	TKW	2D	27,046	eGWAS(BRR,gBLUP)	lipid metabolic process, signal transduction,lipid catabolic process,intracellular signal transduction	intracellular anatomical structure	phosphatidylinositol phospholipase C activity,phosphoric diester hydrolase activity
23	rs16199	TGCAGCAGCAACCACCACCATGGAAAGAGAGAGACAGAGACGGTGAGCTCCTCTGGACAGCGAG	TKW	2D	28,183	eGWAS(BRR,gBLUP,rrBLUP)	lipid metabolic process	-	oxidoreductase activity, acting on paired donors, with oxidation of a pair of donors resulting in the reduction of molecular oxygen to two molecules of water
24	rs25087	TGCAGCCGCAGAAACATGACCGCGCTGACACCGACCATCCTGCCCGCCGCGCCGTCGCCGACGA	FS	3 A	53,669	eGWAS(BRR,gBLUP)	-	-	protein binding
25	rs10286	TGCAGATTGGAATTTCTGAAAGGCCTCCACAAGATGAAGGAAGCAACGATCGATCCG	PH	5B	35,359	eGWAS(BRR,gBLUP)	-	-	hydrolase activity
26	rs19868	TGCAGCATGGAGTTTAAAAATATTCAATGGTAATTTACCAGACCGAAAGACAAATAAGCAATGC	PH	3B	18,217	eGWAS(rrBLUP)	-	-	nucleotide binding, nucleic acid binding, RNA helicase activity,helicase activity,ATP binding
27	rs27041	TGCAGCCTCTCTACCTTAGAGATCTTGGGGATGACCACCGTGTTCCTCTGGAGGCCCCACCGAG	SW	3B	22,764	eGWAS(rrBLUP)	-	-	oxidoreductase activity, aldose-6-phosphate reductase (NADPH) activity,D-threo-aldose 1-dehydrogenase activity
28	rs3365	TGCAGACACTAGTATCATTGGAAGCACAGGATGAGTCCGTTAGACAGTTGGGGGAGCTGAGGCA	TKW	4 A	61,015	eGWAS(rrBLUP)	-	-	ADP binding
29	rs31074	TGCAGCGCTATGGTAGCTTTGGTTGGTAGTTACTCTGAACCGAGATCGGAAGAGCGGGATCACC	PH	4 A	125,958	eGWAS(BRR,gBLUP)	-	integral component of membrane	-
30	rs17272	TGCAGCAGCGGCGGGAGCATAGGATCGTGGAGAGGGAGCAGGGACGGCGAGCTTACGGAGCGGG	PH	4 A	125,958	eGWAS(gBLUP)	transmembrane transport	integral component of membrane	oligopeptide transmembrane transporter activity

Abbreviations: *PH* Plant height, *GY* Grain yield, *GN* Grain number per spike, *TKW* Thousand kernel weight, *SW* Spike weight, *SA* Spike area, *SH* Spike harvest index, *SF* Spike fertility

### Genomic prediction

**Fig. 7:**
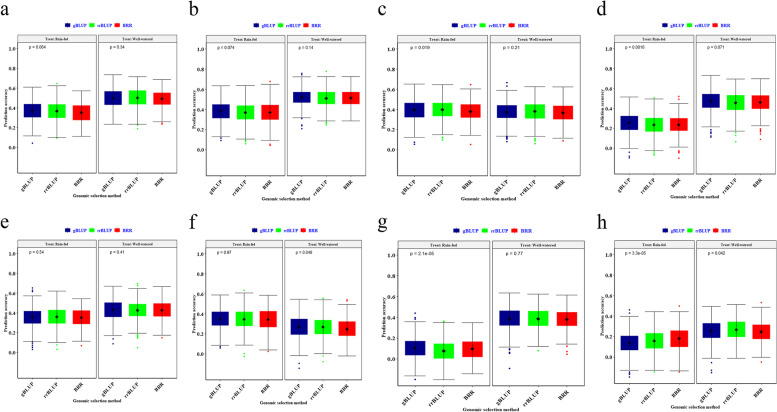
The effect of genomic selection (GS) method on genomic prediction (GP) accuracy for agronomic traits for Iranian landraces and cultivars in the well-watered environment. A-H) The prediction accuracy for gBLUP, rr-BLUP, and BRR-based genomic selection (GS) is demonstrated with blue, green and red colors, respectively. The boxplots show the first, second (median), and third quartile. The middle points indicate a mean of GP accuracies for the trait of interest. **a** Plant height; **b** Grain yield; **c** Grain per spike; **d** Thousand kernel weight; **e** Spike weight; **f** Spike area; **g** Spike harvest index; **h** Spike fertility

The gBLUP, rrBLUP and BRR approaches using imputed SNPs led to the identification of the highest prediction accuracies for 5, 3, and 1 phenotypes in rain-fed, and 5, 3, and zero phenotypes in well-irrigated environments, respectively (Fig. 7). Under rain-fed, the highest prediction accuracy was determined via the gBLUP model for GY (0.381), PH (0.369), SA (0.347), SH (0.104), TKW (0.253), via the rrBLUP for GN (0.396), SW (0.359), via the BRR for SF (0.179). Under well-watered, the highest prediction accuracies were determined via the gBLUP for GY (0.521), SA (0.269), SH (0.384), SW (0.432), TKW (0.470), via the rrBLUP for GN (0.379), PH (0.499), and SF (0.265) (Fig. 7).

## Discussion

Shedding light on the genetic mechanisms controlling quantitative traits such as grain yield in wheat represents an opportunity for the improvement of drought tolerance. To achieve this goal, this experiment aimed at exploring the structure of the population and at uncovering MTAs in Iranian wheat accessions. Significant, positive correlations among the wheat characteristics confirmed the value of the data in the current GWAS analysis. This is evidenced by Laido et al. [26] who highlighted the relationship between morphological characteristics having a high correlation to detect relevant QTLs.

High correlation occurring between agronomic traits can be justified by indirect or direct contributions of one trait to another [27]. Taking a look at the wheat genome, genomic regions responsible for such agronomic characteristics can be equivalent. This is supported by the presence of multi-trait correlations where one gene has a pleiotropic impact on highly-associated characteristics [2]. For example, Mwadzingeni et al. [8] showed that one locus controls several wheat properties such as grains per spike, spike length, and plant height, which are highly linked often [28]. Such observations support the requirement to confirm if such locus is not also linked to another trait, because it shares similar sequences with the regions responsible for the latter trait. Some loci, however, affect only one crop property [8].

Breeding value-clustering by using BRR, gBLUP, and rrBLUP had 77, 68, and 83% similarity with the trait mean method in the terms of wheat accessions grouping, respectively. This indicates that rrBLUP can categorize wheat accessions more accurately than the other methods. Moreover, rrBLUP with the most similarity with the trait mean method in the terms of discovered significant markers, suggesting its potential in uncovering SNPs. As a result, rrBLUP model can detect genetic impacts in wheat populations better than other models. Overall, obtaining the best outcomes from the breeding value-based methods depend on the genetic architecture of trait, genetic variation, etc. [18].

### Linkage disequilibrium of markers

Of the results, the SNPs covered the wheat genome well. The SNPs were higher in genome B. The higher frequency of SNPs in genome B results from the evolutionary events [29]. Genomes D had the highest LD followed by genome A, followed by genome B. At the chromosome level, the strongest LD was recorded between marker pairs on chr 4 A. The fact that cultivars exhibited higher LD in contrast to landraces, particularly in the genome D, is presumably a consequence of selection throughout the time of breeding efforts [30]. The presence of closely linked marker pairs with non-significant LDs and marker pairs in LD over a long distance in this research has been shown previously in wheat and other crops [8, 31]. This reflects that LD is not static because LD can be affected by various elements including genetic admixture [8].

### Population structure of Iranian wheat accessions

The population under consideration was divided into four distinct sub-populations. This is expected because the wheat accessions have diverse pedigrees. Of course, the presence of common parents or origins in the pedigree of accessions often leads to some relationships among them [2]. The findings derived from the population substructure analysis are beneficial in following superior parents that can be used in the improvement of wheat tolerance to drought stress conditions [3]. Therefore, latter researchers can utilize this genetic pool to employ the genetically disparate accessions, which in turn exhibit wheat farmer-preferred properties.

### SNPs and MTAs for wheat agronomic traits

From a brief look at the number of SNPs, lower significant SNPs were recorded under drought than normal conditions, reflecting GWAS analysis for exploring drought tolerance is affected greatly by environment*genotype interactions [8].

This experiment led to discovering of a total of 29 and 30 highly significant MTAs in normal and drought environmental conditions, correspondingly. Albeit only those associations at *P* < 0.0001 were regarded as significant, the rest of these MTAs may be helpful for enhancing wheat tolerance to drought stress. These associations can be located in genomic regions affecting the agronomic characteristics. The MTAs for yield appeared significant at a higher *P* value, because this trait is highly complicated in genetic nature with low heritability [32].

To date, many attempts have been focused on locating QTLs and genes affecting wheat traits in drought environments for facilitating marker-assisted breeding [2, 3]. The MTAs detected in this study are added to the previous pool of candidate genes and markers. However, it is a challenging task to align our results with earlier works because of the use of disparate reference genomes than the IWGSC Ref.Seq, the lack of accurate genomic locations, or the utilization of various markers (GBS-derived SNP vs. SSR and DART) [2, 3, 5, 9]. Of course, detection of MTAs on the same chromosome as previous projects increases the assurance of these MTAs.

Four MTAs for grain yield were recorded on chr 3B, 4 A, 5 A, and 3D in this study. Earlier research efforts have discovered MTAs/QTLs for grain yield on wheat chr 7B [31, 33, 34], 7 A [31, 34–36], 5B [15, 31, 34], 3D [34], 3 A [31, 34, 37, 38], 2B [34, 37–40], and 1B [34, 38, 39]. Thus, MTAs on chr 3B, 4 A, and 5 A have not been reported and they are new for wheat yield. Six MTAs for TKW were found on chr 5 A, 1B, 3B, 6B, 1D, and 2D. Earlier reports have detected MTAs/QTLs for TKW on chr 7D [35], 7B [31], 5B [41], 3B [35], 3 A [40, 41], 2D [39], 2B [31, 35, 39, 42], 2 A [35], 1 A [31, 39–41] and 1B [43]. For plant height, two MTAs were revealed on each of chr 5B, 6B, and 2D. All 21 chromosomes carry genes that control plant height in wheat [42, 44, 45]. Up to now, 24 reduced height (*Rh*t) genes (*Rht1–Rht24*) are catalogued in wheat [46, 47], where *Rht8* on chromosome arm 2DS has been extensively explored [48, 49]. We could locate only two QTLs to chromosome 2DL, whereas the ones reported by Borner et al. [50], on chromosome 2DS could not be detected. Other MTAs detected in our research effort were responsible for grains per spike, spike weight, spike fertility spike area, and spike harvest index. Some of the MTAs detected in this study were involved in the following important biological and molecular processes: metal ion binding, monooxygenase, acyltransferase, oxidoreductase‎, acyltransferase, methyltransferase, peptidase, and dependent microtubule motor activity. The gBLUP with the most similarity (80.98 and 71.28% in well-watered and rain-fed environments) with the trait mean method in the terms of discovered significant SNPs, suggesting the potential of gBLUP in uncovering SNPs. The results show that the gBLUP method performs better than the rrBLUP and BRR methods in terms of predicting the accuracy of genomic breeding values. In gBLUP, genomic relationships are used to estimate an individual’s genetic merit. Genomic relationships are estimated based on DNA marker information for this purpose. To make better predictions of merit, the matrix defines the covariance between individuals on the basis of observed similarity rather than expected similarity based on pedigree. Several studies have described the gBLUP method for estimating genomic breeding values [51–54]. Research shows that gBLUP and rrBLUP are similar models. One of the advantages of gBLUP over rrBLUP is the reduction of the dimensions of the mixed equations to the number of people in the reference population, the calculation of accuracy and error predicting corrective values ​​as commonly used in pedigree methods and combining The information of genotyped and non-genotyped individuals was mentioned simultaneously in the mixed equations [18].

Based on the GO results, the BRR and gBLUP methods were able to better identify the relationship between the studied traits, respectively, and were most similar to the pGWAS results. Generally speaking, genes/markers affecting a trait under drought also are responsible for that trait under normal conditions [8]. Ideally, the impacts of such genes/markers may not be influenced by any moderate changes in environmental conditions, thus they can be helpful in gene introgression or marker-assisted selection when adaptation improvement [55]. Some genes/markers, on the other hand, may affect specific traits differentially under various conditions [55].

Our findings suggested that genomic prediction is a helpful tool for predictive characterization of wheat genotypes, permitting phenotyping to be limited to a fraction of the germplasm rather than the whole collection [56–58]. Similarly, Kehel et al. [59] stated that genomic selection can be used within wheat accessions to predict key traits with an accuracy of more than 0.7, more especially for the traits with high to moderate heritability. Accounting for stratified populations is usually carried out by the first five principal components as covariates in a prediction model [57, 60, 61]. As expected, a significant population structure was identified in the Iranian wheat landraces, with the first five eigenvalues accounting for 30.5% of genetic diversity. The population structure indicated a negative effect on performance in GWAS and GP models, which was also exhibited in other researches [61, 62]. Of our observations, the highest prediction accuracy was achieved via the gBLUP model. Shabannejad et al. [18] evaluated classic approaches for exploiting GP accuracy by BRR, gBLUP, rrBLUP models in normal and drought environments in wheat cultivars and landraces. They identified the highest GP accuracies via the gBLUP and BRR method. The authors observed that obtaining the highest GP accuracy depends on the genetic variation, genetic architecture of trait, level of LD, and the genomic selection approach. As a result, the gBLUP model can detect genetic impacts in wheat populations better than other genomic prediction models.

## Conclusion

MTAs are the key elements to detecting genomic regions related to wheat agronomic traits under drought stress. The current experiment found 29 and 30 highly significant MTAs under normal and drought conditions. The markers detected would be useful genomic sources for cloning and fine mapping of underlying genes, and for conducting gene introgression and marker-based selection in wheat under normal and drought conditions. A further research attempt is needed for validating the markers detected in the current project using a larger wheat population.

## Methods

### Plant material and experimental conditions

A field research effort was performed in two growing seasons (2018-19 and 2019-20) under rain-fed (drought) and well-watered (normal) conditions at the research farm, University of Tehran, Iran. In this study, 90 cultivars and 208 landraces (Table S2) of wheat were investigated in an alpha-lattice experiment with two replications. The wheat accessions were cultivated in the plots including four rows (1*1 m^2^) at 0.5 m intervals. In the well-watered crops, the threshold of irrigation was regarded based on 40 mm evaporation from a standard pan. The reference crop evapotranspiration [ET_0_ = E_pan_× K_pan_; where *K*_pan_ is a pan coefficient (0.8) for each month and *E*_pan_ is the evaporation depth from the pan surface (40 mm)] and crop coefficient [*K*_C_] were estimated to measure evapotranspiration (ET_C_ = K_C_ × ET_0_) [63]. The time of irrigation was determined from the ratio of the assigned water for 1400 m^2^ (the cultivation area of total genotypes in two replications) to water discharge (10.8 m^3^/h). The volume of water required for each hectare (m^3^/ha) was calculated via the depth of ET_0_ (mm) multiplied by ten. The rain-fed crops were exposed to rainfall, which was the only accessible water source. The monthly rainfall pattern for the growing seasons is represented in Table S3. At the maturity stage, 20 plants were harvested from the middle rows of plots to measure traits, including spike fertility (ratio of grain number to spike weight), thousand-kernel weight (g), grain yield (g per plant), grain number per spike, spike weight (g), spike harvest index (ratio of spike grain weight to spike weight, %), spike area (cm^2^), and plant height (cm).

### GBS analysis

To sequence wheat accessions, this experiment followed the procedure as explained by Alipour et al. [29] to establish the GBS libraries. After trimming reads to 64 bp and categorizing them, single nucleotide polymorphisms were discovered by internal alignment. SNPs were called through the UNEAK GBS pipeline, where SNPs with low- allele frequency < 1% and low-quality scores < 15 were discarded to reduce false positives. The SNP imputation process was implemented by available allele frequencies in BEAGLE V.3.3.2 [64]. The LD was calculated by the TASSEL V.5 [65]. The W7984 reference genome was adopted in the recent study because of fulfilling the highest accuracy of imputation among the wheat references [30].

### Structure of wheat population

Population structure in the Iranian wheat accessions was revealed by STRUCTURE V.2.3.4. In this software, the parameters were set at 30,000 burn-in periods, with 30,000 MCMC iterations after burn-in [66]. To permit the picking up of repetition with the highest value of Ln likelihood, 10 replications were run for K values of 1 to 10. By using TASSEL software, genotypic data of wheat accessions were imputed [67]. Moreover, principal component analysis (PCA) was conducted to verify the STRUCTURE outcome. To determine the accession relationships, a neighbor-joining analysis was carried out by *TASSEL V.5.* Linkage disequilibrium (LD) was determined through R^2^ value, squared allele frequency correlation, from which the significant allele pairs were estimated by 1,000 permutations.

### Trait mean-based GWAS (pGWAS)

The mixed linear model (MLM) was followed to estimate the marker impacts on the wheat population. The general linear model was conducted by population structure matrix (Q) integrated as a covariate for correcting the effect of subpopulations. The mixed linear model was performed by both the family structure matrix (Kinship, K) and Q for controlling both errors of type I and II. The association mapping was implemented using MLM functions of *TASSEL V.5.* To correct for multiple test, a false discovery rate was utilized to declare significant MTAs [66, 68]. For a better answer in the recent study, only the outcomes of the MLM procedure were given. There are several methods to determine the threshold in GWAS and all of them have some advantage and disadvantage. But, the most important thing is confirming the results using further analysis. Here the threshold -logP > 3 was considered to find higher number of significant SNPs and identify the important ones using GO and pathway analysis. While from the threshold of -logP > 5 was considered to identify very significant and important SNPs. To explore associations between genotype and phenotype, a Manhattan plot was obtained using the CMplot package [69].

### Breeding value-based GWAS (eGWAS)

Three methods rrBLUP [70], BRR [71], and gBLUP [72] using the Intelligent Prediction and Association Tool (iPat) software were used to obtain the breeding values. A mixed linear model (MLM) was used to estimate the effects of markers using breeding values on wheat populations [9].

### Annotation of putative candidate MTAs

The ensemble-gramene database was employed to extract the molecular and biological functions of SNPs in the gene ontology by using the IWGSC RefSeq V.2.0, which has been provided for the Chinese Spring [http://www.gramene.org/]. Furthermore, the significant SNPs were analyzed via KOBAS version 2.0 for gene ontology enrichment analysis in KEGG [https://www.genome.jp/kegg/].

### Genomic prediction strategies

GP was calculated by various approaches: BRR [71, 73], gBLUP [72, 73], and rrBLUP [70, 73]. All of the analyses were performed by iPat [74]. For the population, 20% of genotypes were assigned randomly to a validation set and all of the residuals were utilized as a training set. This process was reiterated 100 times for all of the prediction approaches. The GP accuracy was calculated as Pearson’s correlation (r) between BLUPs and GEBVs over the validation and training sets [75].

### Statistical analysis

The descriptive statistics and correlation analysis were implemented by R V.4.1 using the dplyr, ggpubr, psych, and ggplot2 packages. Heatmap analysis was carried out using heatmap.2 function in gplots R package to classify wheat accessions.

## Supplementary Information

**Additional file 1: Supplementary Table 1.** Mean, coefficient of variation (CV), broad senseheritability (H^2^), and combined analysis of variance based onstudied traits in 298 Iranian wheat landraces and cultivars. **Supplementary Table 2.** Overview on the landraces and cultivars of Iranian wheat studied. **Supplementary Table 3.** Pattern of total monthly precipitation andirrigation for the 2018-19 and 2019-20 cropping seasons. **Supplementary Fig. 1.** Correlation coefficients between the studied agronomic traits for Iranian wheat landraces and cultivars. (A, Well watered; B, Rainfed). Abbreviations: PH, Plant height; GY, Grain yield; GN, Grain number per spike; TKW, Thousand kernel weight; SW, Spike weight; SA, Spike area; SH, Spike harvest index; SF, Spike fertility. **Supplementary Fig. 2.** GWAS results for agronomic traits andbreeding Values of Iranianlandraces and cultivars in well-watered environments. Agronomic traits (A), BRR (B), gBLUP (C), and rrBLUP (D). Abbreviations: PH, Plant height; GY, Grain yield; GN, Grain number per spike; TKW, Thousand kernel weight; SW, Spike weight; SA, Spike area; SH, Spike harvestindex; SF, Spike fertility. **Supplementary Fig. 3.** Manhattan and QQ-plots of highly associatedhaplotypes for and MLM in Iranian wheat landraces and cultivars in well-wateredenvironments. X axis represents chromosomes: 1) 1A, 2) 1B, 3) 1D, 4) 2A, 5) 2B, 6) 2D, 7) 3A, 8) 3B, 9) 3D, 10) 4A, 11) 4B, 12) 4D, 13) 5A, 14) 5B, 15) 5D, 16) 6A, 17) 6B, 18) 6D, 19) 7A, 20) 7B, 21) 7D. **Supplementary Fig. 4.** GWAS results foragronomic traits and breeding Values of Iranian landraces and cultivars inrain-fed environments. Agronomictraits (A), BRR (B), gBLUP (C), and rrBLUP (D). Abbreviations: PH, Plant height; GY, Grain yield; GN, Grain number per spike; TKW, Thousand kernel weight; SW, Spike weight; SA, Spike area; SH, Spike harvest index; SF, Spike fertility. **Supplementary Fig. 5.** Manhattan and QQ-plots of highly associatedhaplotypes for and MLM in Iranian wheat landraces and cultivars in rain-fed environments. X axis represents chromosomes: 1) 1A, 2) 1B, 3) 1D, 4) 2A, 5) 2B, 6) 2D, 7) 3A, 8) 3B, 9) 3D, 10) 4A, 11) 4B, 12) 4D, 13) 5A, 14) 5B, 15) 5D, 16) 6A, 17) 6B, 18) 6D, 19) 7A, 20) 7B, 21) 7D. **Supplementary Fig 6. **The KEGG pathway of metabolic pathways. **Supplementary Fig 7. **The KEGG pathway of oxidativephosphorylation. **Supplementary Fig 8. **The KEGG pathway of biosynthesis of amino acids. **Supplementary Fig 9. **The KEGG pathway of ascorbate and aldarate metabolism. **Supplementary Fig 10.** The KEGG pathway of sulfur metabolism. **Supplementary Fig 11.** The KEGG pathway of fatty acid elongation.

## Data Availability

The datasets generated and analyzed during the current study are available in the Figshare repository [10.6084/m9.figshare.18774476.v1].
