# Supplementary material for: Genome-wide association mapping and genomic prediction of agronomical traits and breeding values in Iranian wheat under rain-fed and well-watered conditions
Source: BMC Genomics. 2022 Dec 15;23:831. doi: 10.1186/s12864-022-08968-w (PMC9753272; doi:10.1186/s12864-022-08968-w)
Supplement: Supplementary file 1 — Additional file 1: Supplementary Table 1. Mean, coefficient of variation (CV), broad senseheritability (H2), and combined analysis of variance based onstudied traits in 298 Iranian wheat landraces and cultivars. Supplementary Table 2. Overview on the landraces and cultivars of Iranian wheat studied. Supplementary Table 3. Pattern of total monthly precipitation andirrigation for the 2018-19 and 2019-20 cropping seasons. Supplementary Fig. 1. Correlation coefficients between the studied agronomic traits for Iranian wheat landraces and cultivars. (A, Well watered; B, Rainfed). Abbreviations: PH, Plant height; GY, Grain yield; GN, Grain number per spike; TKW, Thousand kernel weight; SW, Spike weight; SA, Spike area; SH, Spike harvest index; SF, Spike fertility. Supplementary Fig. 2. GWAS results for agronomic traits andbreeding Values of Iranianlandraces and cultivars in well-watered environments. Agronomic traits (A), BRR (B), gBLUP (C), and rrBLUP (D). Abbreviations: PH, Plant height; GY, Grain yield; GN, Grain number per spike; TKW, Thousand kernel weight; SW, Spike weight; SA, Spike area; SH, Spike harvestindex; SF, Spike fertility. Supplementary Fig. 3. Manhattan and QQ-plots of highly associatedhaplotypes for and MLM in Iranian wheat landraces and cultivars in well-wateredenvironments. X axis represents chromosomes: 1) 1A, 2) 1B, 3) 1D, 4) 2A, 5) 2B, 6) 2D, 7) 3A, 8) 3B, 9) 3D, 10) 4A, 11) 4B, 12) 4D, 13) 5A, 14) 5B, 15) 5D, 16) 6A, 17) 6B, 18) 6D, 19) 7A, 20) 7B, 21) 7D. Supplementary Fig. 4. GWAS results foragronomic traits and breeding Values of Iranian landraces and cultivars inrain-fed environments. Agronomictraits (A), BRR (B), gBLUP (C), and rrBLUP (D). Abbreviations: PH, Plant height; GY, Grain yield; GN, Grain number per spike; TKW, Thousand kernel weight; SW, Spike weight; SA, Spike area; SH, Spike harvest index; SF, Spike fertility. Supplementary Fig. 5. Manhattan and QQ-plots of highly associatedhaplotypes for and MLM in Iranian wheat landraces and cul [file 12864_2022_8968_MOESM1_ESM.docx]

**Supplementary Table 1.** Mean, coefficient of variation (CV), broad sense heritability (H^2^), and combined analysis of variance based on studied traits in 298 Iranian wheat landraces and cultivars.

|  | **Well-watered** | | | | | | |  | **Rain-fed** | | | | | | |
| --- | --- | --- | --- | --- | --- | --- | --- | --- | --- | --- | --- | --- | --- | --- | --- |
| **Trait** | **Mean** | **CV(%)** | **H^2^** | **Mean squares** | | | |  | **Mean** | **CV(%)** | **H^2^** | **Mean squares** | | | |
|  |  |  |  | **Env** | **Rep (Env)** | **Gen** | **Gen× Env** |  |  |  |  | **Env** | **Rep (Env)** | **Gen** | **Gen× Env** |
| GY | 1.7074 | 32.05 | 42.07 | ** | * | *** | *** |  | 0.9340 | 34.33 | 41.50 | ** | ** | *** | *** |
| TKW | 42.100 | 17.02 | 56.11 | ** | * | *** | ** |  | 28.182 | 22.22 | 46.51 | ** | ** | *** | ** |
| GN | 43.867 | 15.79 | 60.07 | ** | * | *** | *** |  | 37.239 | 25.39 | 53.65 | ** | * | *** | *** |
| PH | 103.41 | 10.56 | 69.58 | ns | ** | *** | *** |  | 85.881 | 11.83 | 67.58 | ns | * | *** | *** |
| SW | 2.5152 | 12.77 | 51.21 | ** | * | *** | *** |  | 1.7233 | 6.200 | 55.21 | ** | * | *** | *** |
| SA | 12.181 | 9.740 | 57.39 | ** | * | *** | *** |  | 10.033 | 8.090 | 53.29 | ** | ** | *** | *** |
| SH | 67.824 | 12.04 | 61.34 | ** | * | *** | *** |  | 54.407 | 14.87 | 60.04 | ** | ** | *** | *** |
| SF | 17.594 | 18.03 | 57.07 | ** | * | *** | *** |  | 21.832 | 12.61 | 53.07 | ** | * | *** | *** |

*, ** and *** are signifcant at the probability level of 5%, 1% and 0.1%, respectively.

Abbreviations: PH, Plant height (cm); GY, Grain yield (g per plant); GN, Grain number per spike; TKW, Thousand kernel weight (g); SW, Spike weight (g); SA, Spike area (cm^2^); SH, Spike harvest index (%); SF, Spike fertility.

**Supplementary Table 2.** Overview on the landraces and cultivars of Iranian wheat studied

| **Genetic background: Landraces** | | | | | | |
| --- | --- | --- | --- | --- | --- | --- |
| **No.** | **Region of origin (Province)** | **USDA_PI_NO** |  | **No.** | **Region of origin (Province)** | **USDA_PI_NO** |
| 1 | Gilan | 625281 |  | 105 | Tehran | 621669 |
| 2 | Mazandaran | 625362 |  | 106 | Gazvin | 621704 |
| 3 | Khorasan | 625433 |  | 107 | Gazvin | 621706 |
| 4 | Khorasan | 625661 |  | 108 | Gazvin | 621712 |
| 5 | Khorasan | 625810 |  | 109 | Gazvin | 621716 |
| 6 | Kerman | 626156 |  | 110 | Azarbayjan-Gharbi | 620903 |
| 7 | Kerman | 626158 |  | 111 | Hamadan | 621420 |
| 8 | Kerman | 626215 |  | 112 | Hamadan | 621421 |
| 9 | Sistan-Balouchestan | 626223 |  | 113 | Bakhtaran | 621492 |
| 10 | Sistan-Balouchestan | 626226 |  | 114 | Hamadan | 621565 |
| 11 | Sistan-Balouchestan | 626234 |  | 115 | Mazandaran | 622084 |
| 12 | Markazi | 625080 |  | 116 | Gilan | 622098 |
| 13 | Markazi | 625081 |  | 117 | Gilan | 622099 |
| 14 | Markazi | 625123 |  | 118 | Gilan | 622105 |
| 15 | Markazi | 625127 |  | 119 | Mazandaran | 622247 |
| 16 | Markazi | 625139 |  | 120 | Mazandaran | 622264 |
| 17 | Mazandaran | 625263 |  | 121 | Mazandaran | 622272 |
| 18 | Sistan-Balouchestan | 626260 |  | 122 | Khorasan | 622311 |
| 19 | Sistan-Balouchestan | 626261 |  | 123 | Gazvin | 621717 |
| 20 | Esfahan | 626358 |  | 124 | Gazvin | 621735 |
| 21 | Esfahan | 626360 |  | 125 | Gazvin | 621736 |
| 22 | Esfahan | 626565 |  | 126 | Markazi | 621869 |
| 23 | Esfahan | 626566 |  | 127 | Markazi | 621908 |
| 24 | Esfahan | 626573 |  | 128 | Zanjan | 622063 |
| 25 | Ilam | 626699 |  | 129 | Yazd | 623109 |
| 26 | Hamadan | 626706 |  | 130 | Fars | 623123 |
| 27 | Khorasan | 626736 |  | 131 | Fars | 623125 |
| 28 | Yazd | 626747 |  | 132 | Fars | 623127 |
| 29 | Yazd | 626764 |  | 133 | Esfahan | 623008 |
| 30 | Khorasan | 626776 |  | 134 | Esfahan | 623069 |
| 31 | Esfahan | 626814 |  | 135 | Bakhtaran | 623090 |
| 32 | Esfahan | 626825 |  | 136 | Khorasan | 623091 |
| 33 | Yazd | 626846 |  | 137 | Khorasan | 622379 |
| 34 | Markazi | 626855 |  | 138 | Esfahan | 622894 |
| 35 | Fars | 626872 |  | 139 | Azarbayjan-Gharbi | 623266 |
| 36 | Kerman | 626908 |  | 140 | Bakhtaran | 623274 |
| 37 | Gilan | 626923 |  | 141 | Hamadan | 623291 |
| 38 | Gilan | 626924 |  | 142 | Yazd | 623318 |
| 39 | Hormozgan | 626932 |  | 143 | Fars | 623338 |
| 40 | Azarbayjan-Shargi | 626881 |  | 144 | Bakhtaran | 623344 |
| 41 | Fars | 626883 |  | 145 | Kordestan | 623345 |
| 42 | Azarbayjan-Shargi | 626895 |  | 146 | Kerman | 623377 |
| 43 | Azarbayjan-Shargi | 626904 |  | 147 | Kerman | 623379 |
| 44 | Zanjan | 627072 |  | 148 | Azarbayjan-Gharbi | 623136 |
| 45 | Khouzestan | 627099 |  | 149 | Fars | 623139 |
| 46 | Zanjan | 627102 |  | 150 | Azarbayjan-Gharbi | 623161 |
| 47 | Mazandaran | 627103 |  | 151 | Azarbayjan-Gharbi | 623162 |
| 48 | Khorasan | 627189 |  | 152 | Gilan | 623169 |
| 49 | Zanjan | 627055 |  | 153 | Khorasan | 623176 |
| 50 | Gilan | 627057 |  | 154 | Azarbayjan-Gharbi | 623510 |
| 51 | Markazi | 627061 |  | 155 | Bakhtaran | 623905 |
| 52 | Kerman | 627066 |  | 156 | Bakhtaran | 623908 |
| 53 | Hormozgan | 626933 |  | 157 | Bakhtaran | 623909 |
| 54 | Kerman | 626943 |  | 158 | Bakhtaran | 623953 |
| 55 | Azarbayjan-Gharbi | 626958 |  | 159 | Hamadan | 623980 |
| 56 | Esfahan | 626978 |  | 160 | Hamadan | 624215 |
| 57 | Khouzestan | 627036 |  | 161 | Ilam | 624240 |
| 58 | Khouzestan | 627038 |  | 162 | Ilam | 624251 |
| 59 | Azarbayjan-Gharbi | 627043 |  | 163 | Ilam | 623475 |
| 60 | Gilan | 627054 |  | 164 | Ilam | 623503 |
| 61 | Khorasan | 627236 |  | 165 | Bakhtaran | 623506 |
| 62 | Yazd | 627299 |  | 166 | Bakhtaran | 623507 |
| 63 | Hormozgan | 627356 |  | 167 | Bakhtaran | 623508 |
| 64 | Markazi | 627359 |  | 168 | Kerman | 623382 |
| 65 | Kerman | 627360 |  | 169 | Sistan-Balouchestan | 623417 |
| 66 | Bakhtaran | 627385 |  | 170 | Azarbayjan-Shargi | 623421 |
| 67 | Zanjan | 627399 |  | 171 | Azarbayjan-Shargi | 623428 |
| 68 | Azarbayjan-Shargi | 627410 |  | 172 | Ilam | 623473 |
| 69 | Bakhtaran | 627414 |  | 173 | Hamadan | 624596 |
| 70 | Bakhtaran | 627416 |  | 174 | Bakhtaran | 624804 |
| 71 | Bakhtaran | 627417 |  | 175 | Bakhtaran | 624805 |
| 72 | Hamadan | 627423 |  | 176 | Ilam | 624818 |
| 73 | Khorasan | 627460 |  | 177 | Ilam | 624837 |
| 74 | Yazd | 627484 |  | 178 | Ilam | 624838 |
| 75 | Azarbayjan-Shargi | 627787 |  | 179 | Ilam | 624846 |
| 76 | Kerman | 627842 |  | 180 | Ilam | 624849 |
| 77 | Sistan-Balouchestan | 627845 |  | 181 | Ilam | 624861 |
| 78 | Sistan-Balouchestan | 627849 |  | 182 | Kordestan | 624315 |
| 79 | Sistan-Balouchestan | 627852 |  | 183 | Bakhtaran | 624378 |
| 80 | Sistan-Balouchestan | 627853 |  | 184 | Bakhtaran | 624381 |
| 81 | Mazandaran | 627856 |  | 185 | Hamadan | 624576 |
| 82 | Zanjan | 627873 |  | 186 | Hamadan | 624580 |
| 83 | Esfahan | 627688 |  | 187 | Hamadan | 624582 |
| 84 | Yazd | 627723 |  | 188 | Hamadan | 624585 |
| 85 | Azarbayjan-Shargi | 627760 |  | 189 | Tehran | 624944 |
| 86 | Azarbayjan-Shargi | 627551 |  | 190 | Tehran | 624946 |
| 87 | Kordestan | 627587 |  | 191 | Tehran | 624947 |
| 88 | Esfahan | 627616 |  | 192 | Tehran | 624956 |
| 89 | Azarbayjan-Shargi | 627881 |  | 193 | Tehran | 624963 |
| 90 | Azarbayjan-Shargi | 627883 |  | 194 | Gazvin | 624980 |
| 91 | Mazandaran | 627905 |  | 195 | Gazvin | 624983 |
| 92 | Markazi | 627908 |  | 196 | Gazvin | 624985 |
| 93 | Markazi | 627948 |  | 197 | Gazvin | 624990 |
| 94 | Hamadan | 627963 |  | 198 | Markazi | 625047 |
| 95 | Zanjan | 627987 |  | 199 | Ilam | 624863 |
| 96 | Bakhtaran | 627990 |  | 200 | Ilam | 624864 |
| 97 | Bakhtaran | 628012 |  | 201 | Kordestan | 624894 |
| 98 | Mazandaran | 628084 |  | 202 | Kordestan | 624900 |
| 99 | Markazi | 628088 |  | 203 | Kordestan | 624901 |
| 100 | Esfahan | 628114 |  | 204 | Hamadan | 624910 |
| 101 | Ilam | 628189 |  | 205 | Hamadan | 624911 |
| 102 | Kordestan | 621619 |  | 206 | Hamadan | 624925 |
| 103 | Tehran | 621650 |  | 207 | Tehran | 624939 |
| 104 | Tehran | 621668 |  | 208 | Tehran | 624941 |

| **Genetic background: Cultivars** | | | |
| --- | --- | --- | --- |
| **No.** | **Variety Name** | **Introduced year** | **Growth Habit** |
| 209 | 4820 | 1951 | Spring |
| 210 | ADL | 1976 | Spring |
| 211 | AFLAK | 2010 | Spring |
| 212 | AKBARI | 2006 | Spring |
| 213 | ALBORZ | 1978 | Spring |
| 214 | ARTA | 2006 | Spring |
| 215 | AZADI | 1979 | Facultative |
| 216 | AZAR | 1957 | Winter |
| 217 | BAYAT | 1976 | Spring |
| 218 | BISTON | 1980 | Spring |
| 219 | CHAMRAN | 1997 | Spring |
| 220 | CHAMRAN-2 | 2013 | Spring |
| 221 | DARAB 1 | 1980 | Spring |
| 222 | DARAB 2 | 1995 | Spring |
| 223 | DASTJERDI | 1960 | Spring |
| 224 | DAYHIM | 1968 | Spring |
| 225 | DN-11 | --- | --- |
| 226 | FALAT | 1990 | Spring |
| 227 | FONG | --- | --- |
| 228 | FONTANA | --- | --- |
| 229 | GAHAR | 1996 | Spring |
| 230 | GHODS | 1988 | Spring |
| 231 | GOLESTAN | 1986 | Spring |
| 232 | HOMA | 2009 | Winter |
| 233 | KARAJ 1 | 1974 | Facultative |
| 234 | KARAJ 2 | 1974 | Winter |
| 235 | KARAJ 3 | 1974 | Winter |
| 236 | KARIM | 2011 | Spring |
| 237 | Gascogne | 1994 | --- |
| 238 | KAVEH | 1980 | Spring |
| 239 | KHAZAR 1 | 1974 | Spring |
| 240 | KOOHDASHT | 2002 | Spring |
| 241 | MAHDAVI | 1995 | Spring |
| 242 | MAROON | 1991 | Spring |
| 243 | MIHAN | 2010 | Winter |
| 244 | MOGHAN 1 | 1974 | Spring |
| 245 | MOGHAN 2 | 1974 | Spring |
| 246 | MOGHAN 3 | 2006 | Spring |
| 247 | MORVARID | 2009 | Spring |
| 248 | MV-17 | 1993 | Winter |
| 249 | NAVID 1990 | 1990 | Facultative |
| 250 | NAZ | 1978 | Spring |
| 251 | NEISHABOUR | 2006 | Spring |
| 252 | NICKNEJAD | 1995 | Spring |
| 253 | OFOGH | 2012 | Spring |
| 254 | OHADI | 2010 | Winter |
| 255 | PANJAMO 62 | 1968 | Spring |
| 256 | PISHGAM | 2008 | Facultative |
| 257 | QABOOS | 2014 | Spring |
| 258 | RAYHANI | 1942 | Spring |
| 259 | RIJAW | 2011 | Facultative |
| 260 | SIOSSON | 1994 | Spring |
| 261 | SHAHI | 1967 | Winter |
| 262 | SHAHPASSAND | 1942 | Winter |
| 263 | SIRVAN | 2012 | Spring |
| 264 | SISTAN | 2006 | Spring |
| 265 | TAK-AB | 2013 | Spring |
| 266 | TOUS | 2002 | Facultative |
| 267 | TOBARI 66 | 1969 | Spring |
| 268 | UROUM | 2009 | Winter |
| 269 | VEE/NAC | 1997 | Spring |
| 270 | ZARE | 2010 | Facultative |
| 271 | ZARRIN | 1995 | Spring |
| 272 | SHANGHAI #7 | --- | Spring |
| 273 | INIA 66 | 1969 | Spring |
| 274 | ARVAND 1 | 1974 | Spring |
| 275 | ROSHAN | 1960 | Spring |
| 276 | RASHID | 1968 | Facultative |
| 277 | SABALAN | 1980 | Spring |
| 278 | DARYA | 2006 | Spring |
| 279 | ATRAK | 1995 | Spring |
| 280 | BAHAR | 2007 | Spring |
| 281 | SEPAHAN | 2006 | Spring |
| 282 | BAM | 2006 | Spring |
| 283 | SHIRAZ | 2002 | Spring |
| 284 | PISHTAZ | 2002 | Spring |
| 285 | HAMOON | 2002 | Spring |
| 286 | DEZ | 2002 | Spring |
| 287 | SHIROODI | 1997 | Spring |
| 288 | MARVDASHT | 1999 | Spring |
| 289 | ZAGROS | 1996 | Spring |
| 290 | TAJAN | 1995 | Spring |
| 291 | ALVAND | 1995 | Facultative |
| 292 | KAVIR | 1997 | Spring |
| 293 | PARSI | 2009 | Spring |
| 294 | SIVAND | 2009 | Spring |
| 295 | BEZOSTAYA | 1969 | Winter |
| 296 | AKOVA | 1958 | Winter |
| 297 | SHAHRYAR | 2002 | Winter |
| 298 | AZAR 2 | 1997 | Winter |

**Supplementary Table 3** Pattern of total monthly precipitation and irrigation for the 2018-19 and 2019-20 cropping seasons.

| **Year** | **Month** | **Max Temperature °C** | **Min Temperature °C** | **Average Temperature °C** | **Average rainfall, mm** | **Average relative humidity** | **Sunny hours** | **Evaporation, mm** |
| --- | --- | --- | --- | --- | --- | --- | --- | --- |
| 2018-2019 | November | 14.561 | 4.104 | 10.900 | 0.93 | 45.810 | 6.893 | 3.068 |
|  | December | 9.242 | -0.119 | 4.671 | 41.11 | 60.134 | 5.065 | 0.000 |
|  | January | 8.406 | -0.613 | 3.668 | 15.04 | 57.750 | 6.652 | 0.000 |
|  | February | 7.871 | -2.254 | 2.536 | 27.99 | 61.429 | 6.868 | 0.000 |
|  | March | 14.216 | 4.623 | 9.271 | 38.44 | 56.847 | 5.942 | 0.179 |
|  | April | 21.093 | 9.563 | 15.110 | 46.65 | 49.954 | 6.587 | 4.497 |
|  | May | 29.229 | 14.261 | 21.935 | 22.01 | 38.722 | 10.435 | 7.377 |
|  | June | 34.159 | 17.597 | 26.083 | 0.00 | 32.304 | 12.763 | 11.676 |
| 2019-2020 | November | 17.080 | 6.383 | 11.520 | 0.63 | 43.479 | 6.960 | 3.189 |
|  | December | 12.303 | 1.652 | 6.671 | 4.71 | 50.419 | 7.226 | 0.000 |
|  | January | 9.077 | -0.055 | 4.052 | 19.84 | 54.476 | 6.526 | 0.000 |
|  | February | 10.739 | 2.039 | 6.464 | 31.73 | 64.755 | 5.829 | 0.000 |
|  | March | 20.558 | 8.377 | 14.652 | 14.11 | 38.952 | 7.303 | 0.000 |
|  | April | 19.983 | 7.793 | 13.633 | 45.81 | 51.413 | 7.563 | 6.714 |
|  | May | 25.513 | 12.061 | 18.432 | 57.07 | 54.907 | 8.287 | 6.161 |
|  | June | 33.807 | 17.347 | 25.583 | 7.23 | 37.492 | 11.100 | 11.143 |
|  |  |  |  |  |  |  |  |  |
|  | **Month** | **ET_0_ (mm)** | **K_C_** | **ET_C_ (mm)** | **Required water per ha (m^3^ /ha)** | **Required water for 1377 m^2^ (m^3^)** | **Water discharge (m^3^ /h)** | **Period of irrigation (h)** |
| 2018-2019  and 2019-2020 | March | 40 | 0.92 | 36.8 | 368 | 50.69 | 10.8 | 4.69 |
|  | April | 40 | 1.33 | 53.2 | 532 | 73.28 | 10.8 | 6.79 |
|  | May | 40 | 1.15 | 46 | 46 | 63.37 | 10.8 | 5.87 |
|  | June | 40 | 0.58 | 23.2 | 232 | 31.96 | 10.8 | 2.96 |

| A | B |
| --- | --- |
| 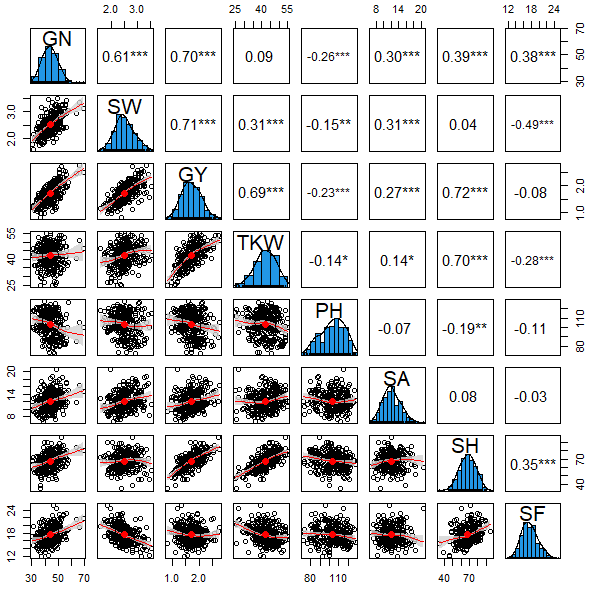 | 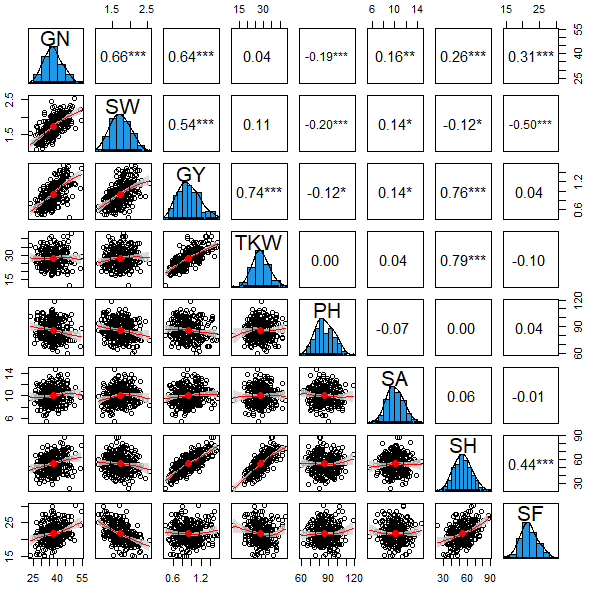 |

**Supplementary Fig. 1** Correlation coefficients between the studied agronomic traits for Iranian wheat landraces and cultivars. (A, Well watered; B, Rain fed). Abbreviations: PH, Plant height (cm); GY, Grain yield (g per plant); GN, Grain number per spike; TKW, Thousand kernel weight (g); SW, Spike weight (g); SA, Spike area (cm^2^); SH, Spike harvest index (%); SF, Spike fertility.

**A**

**Supplementary Fig. 2** GWAS results for agronomic traits and breeding Values of Iranian landraces and cultivars in well-watered environments. Agronomic traits (A), BRR (B), gBLUP (C), and rrBLUP (D). Abbreviations: PH, Plant height (cm); GY, Grain yield (g per plant); GN, Grain number per spike; TKW, Thousand kernel weight (g); SW, Spike weight (g); SA, Spike area (cm^2^); SH, Spike harvest index (%); SF, Spike fertility.

| **Agronomic traits (A)** |  |
| --- | --- |
| Plant height |  |
| 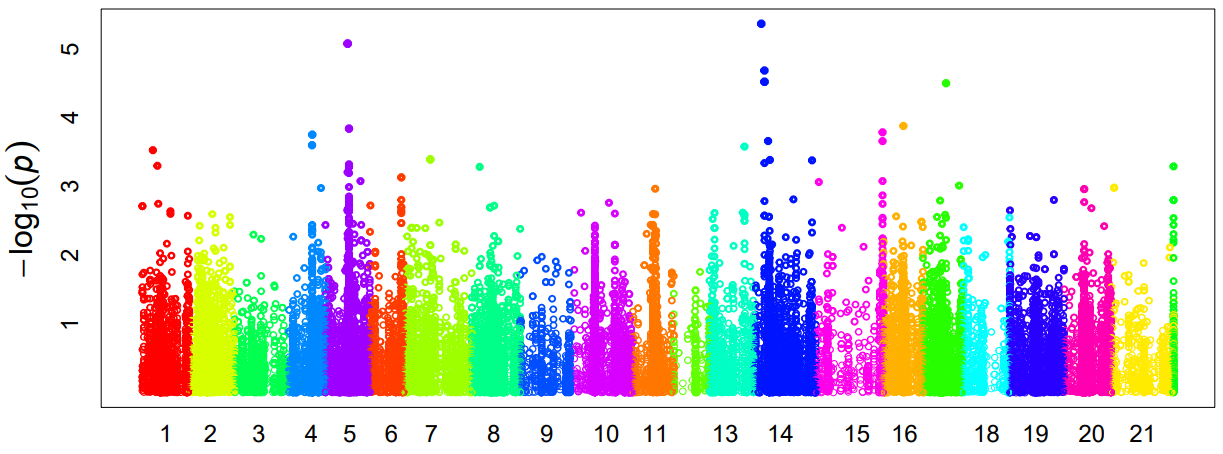 | 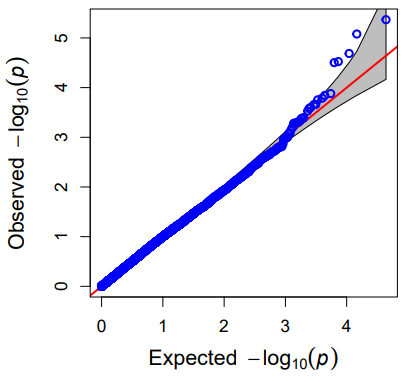 |
| Grain yield |  |
| 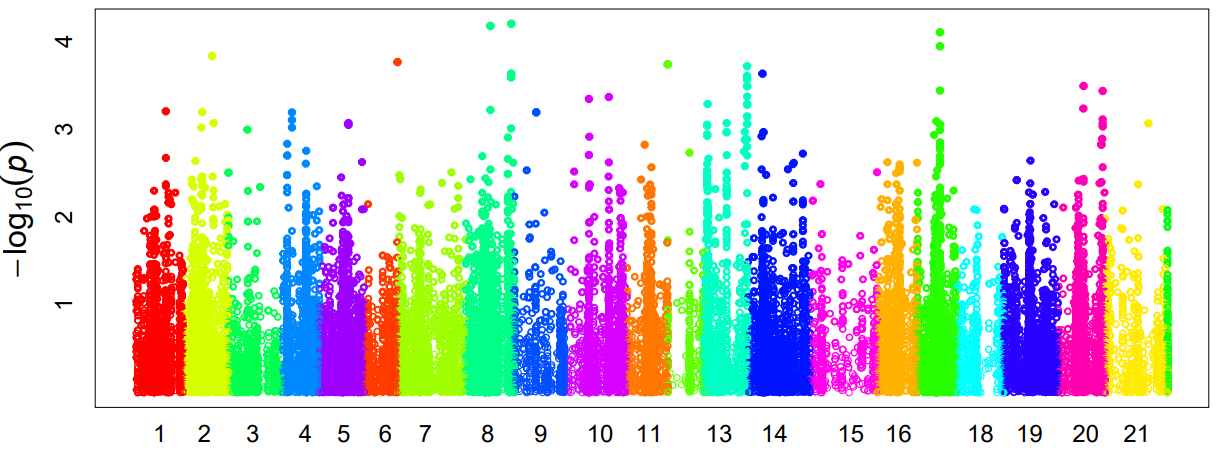 | 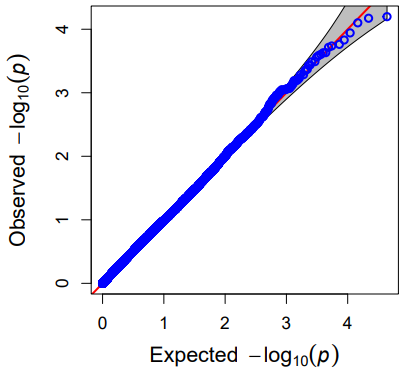 |
| Grain per spike |  |
| 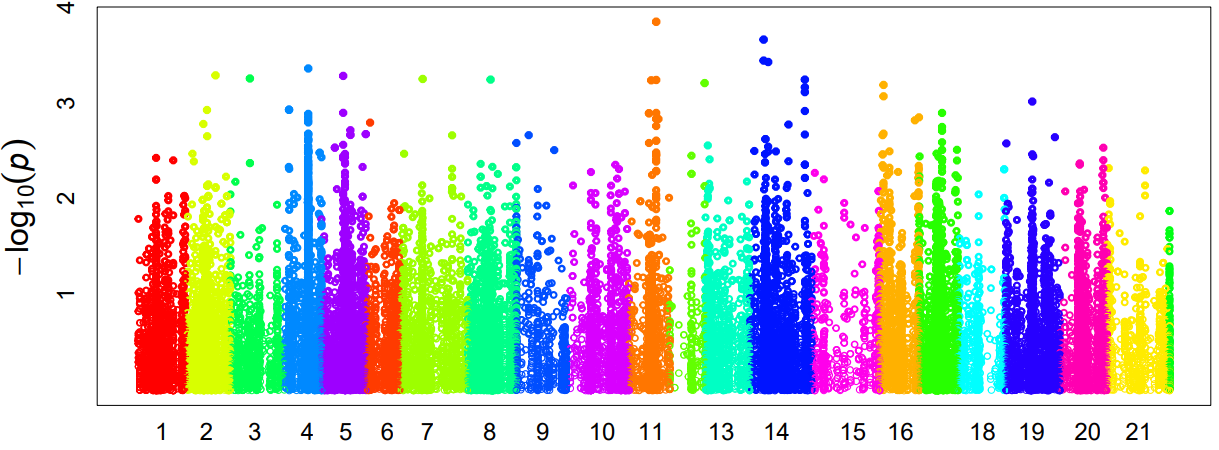 | 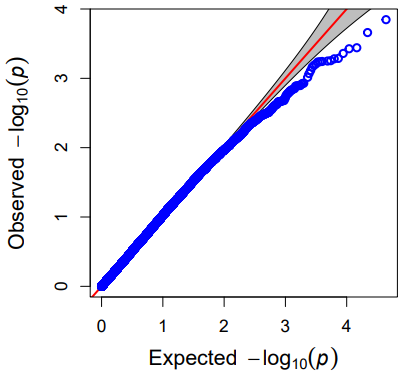 |
| Thousand kernel weight |  |
| 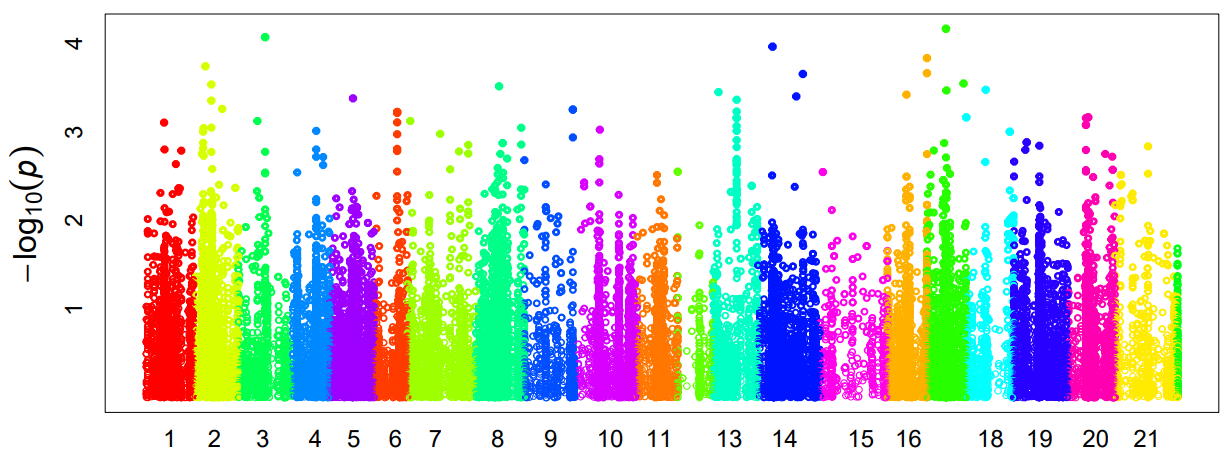 | 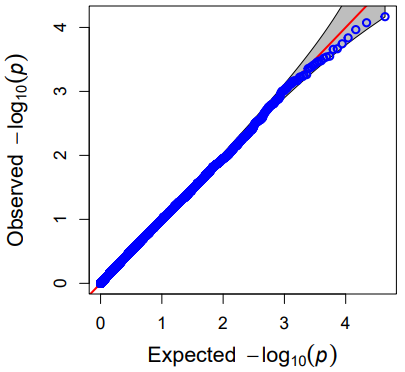 |
|  |  |
|  |  |
|  |  |
| Spike weight |  |
| 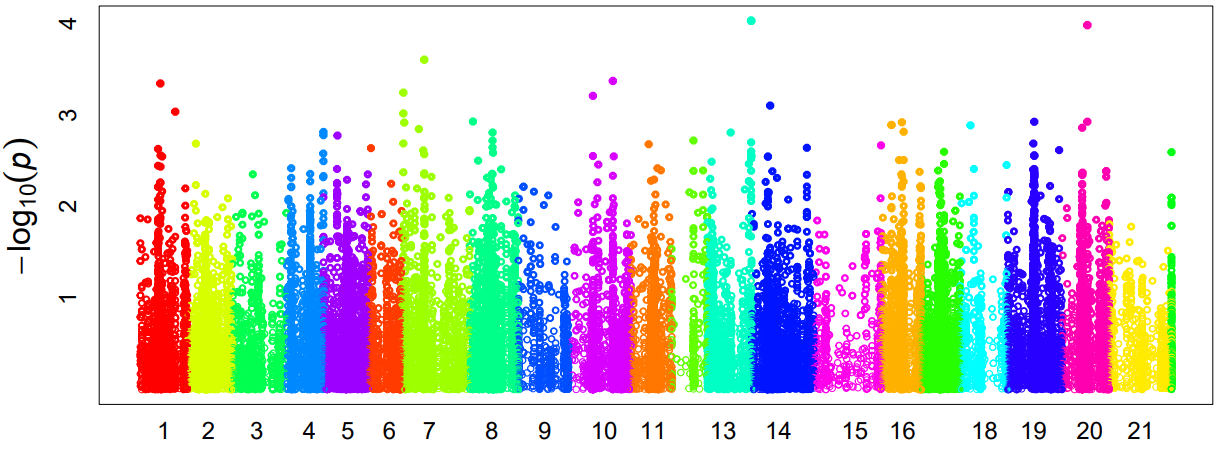 | 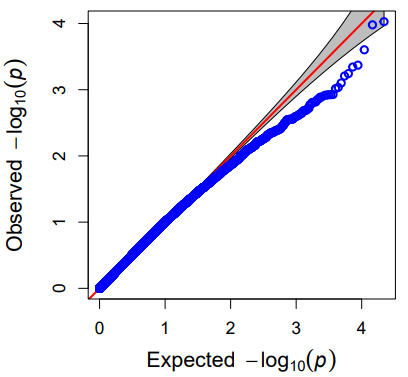 |
| Spike area |  |
| 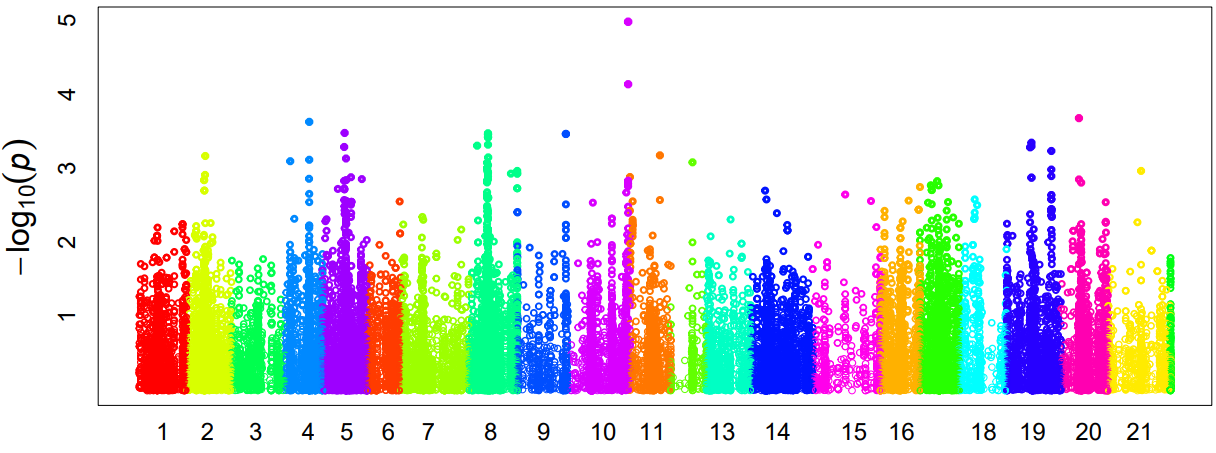 | 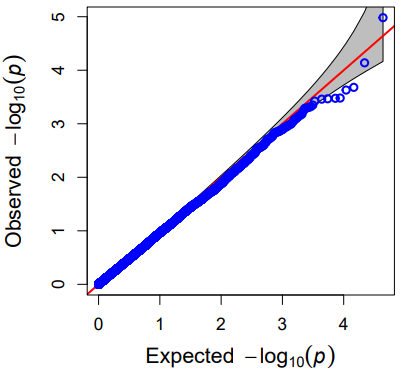 |
| Spike harvest index |  |
| 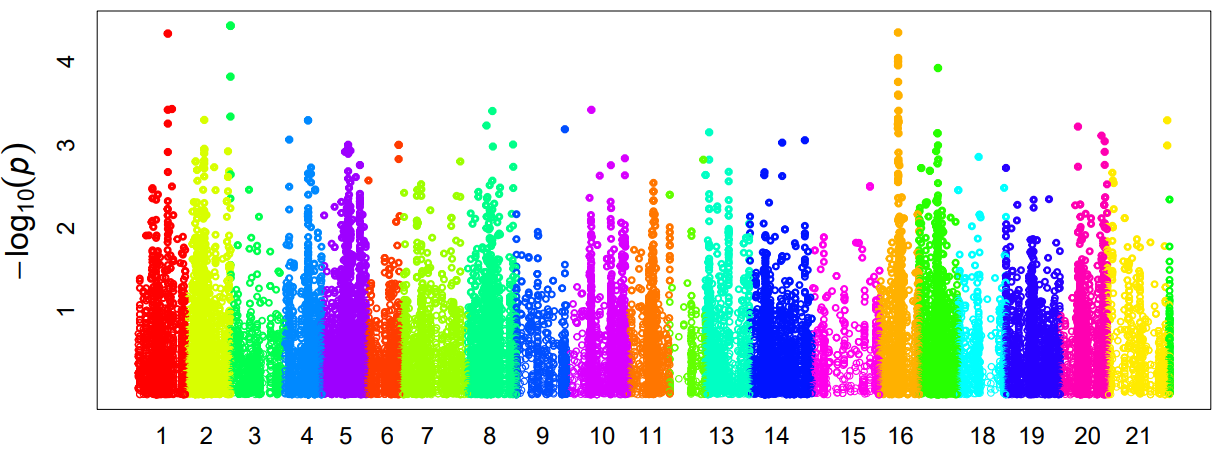 | 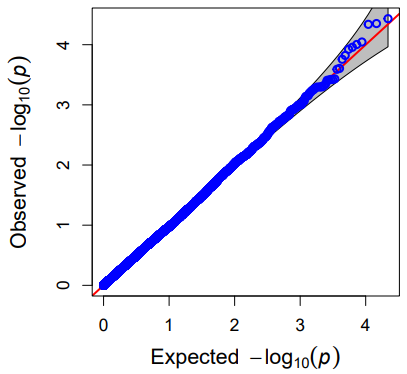 |
| Spike fertility |  |
| 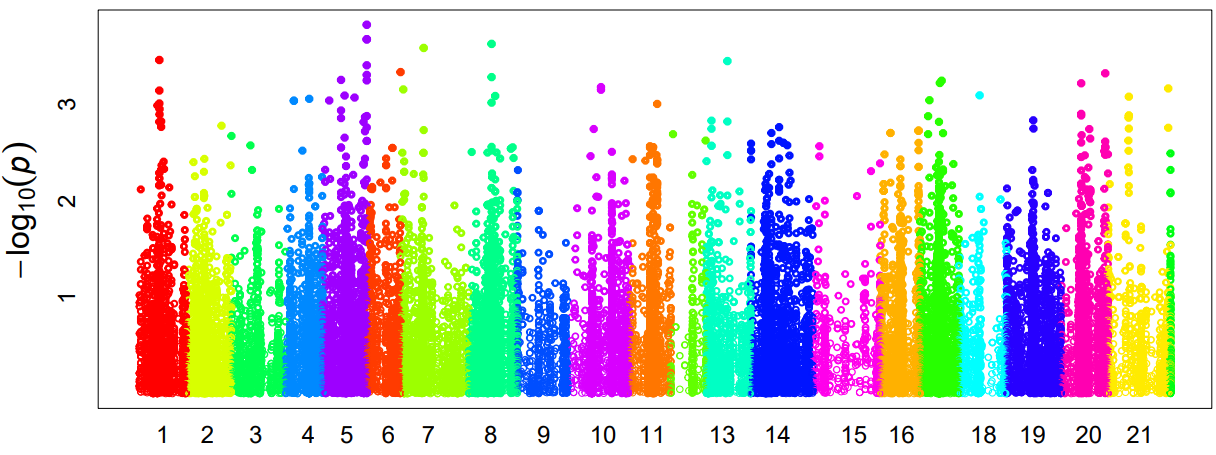 | 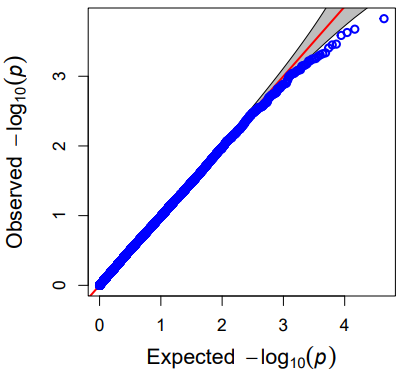 |
|  |  |
| **BRR (B)** |  |
| Plant height |  |
| 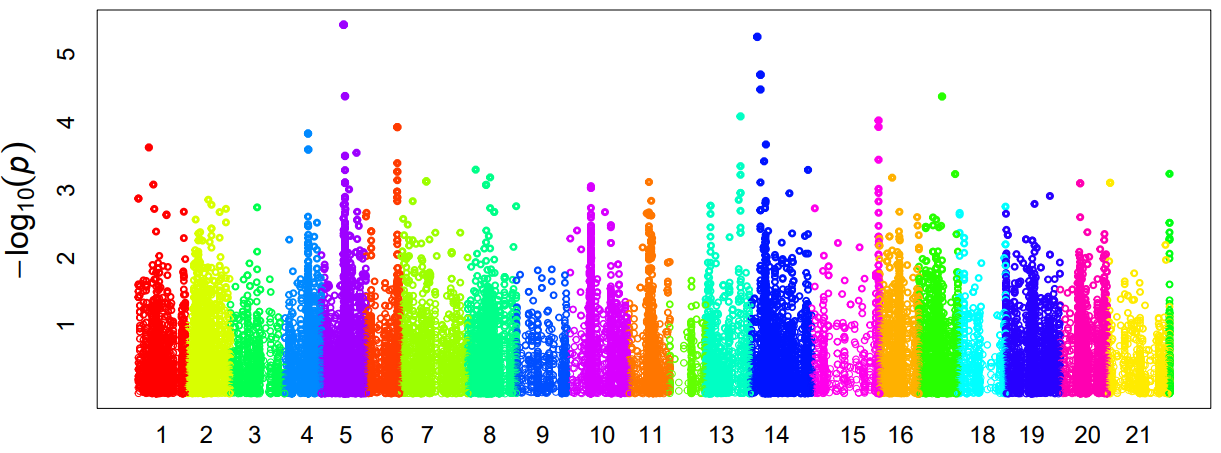 | 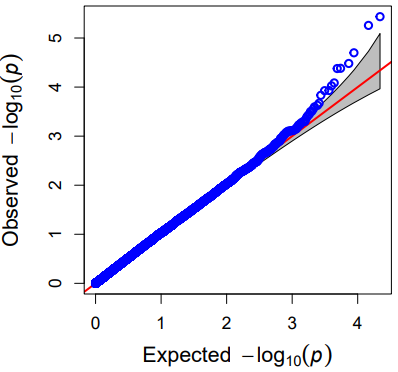 |
| Grain yield |  |
| 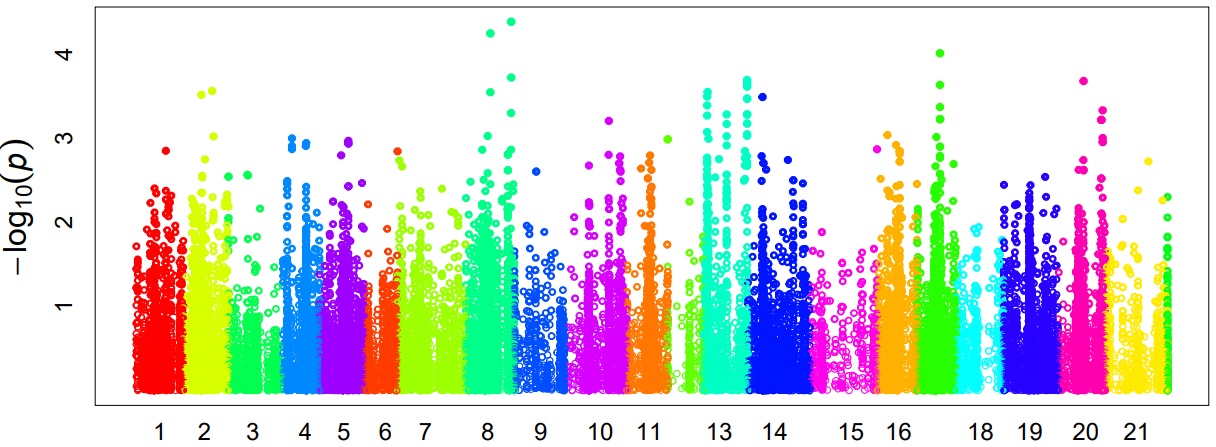 | 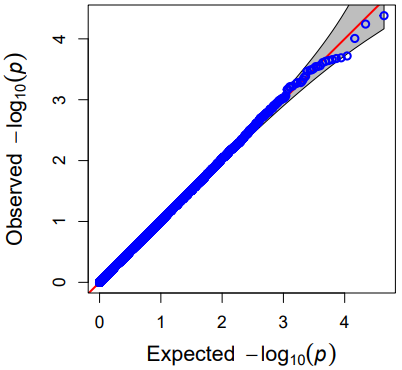 |
| Grain per spike |  |
| 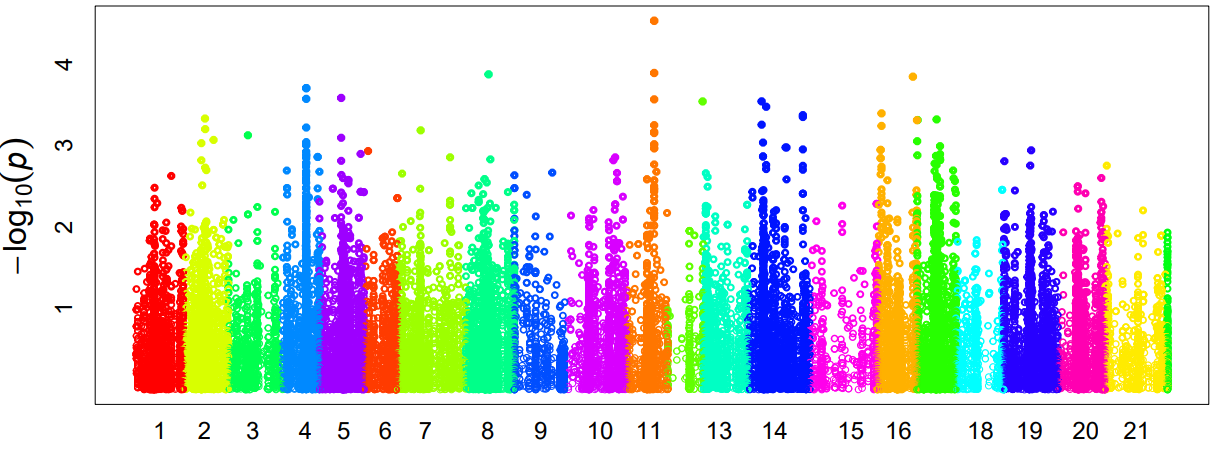 | 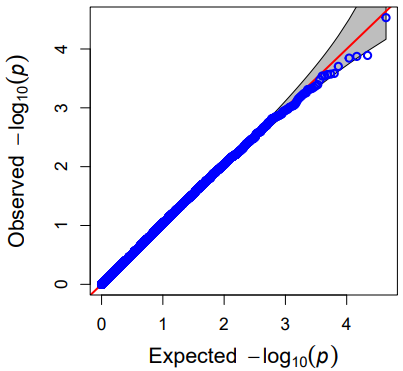 |
| Thousand kernel weight |  |
| 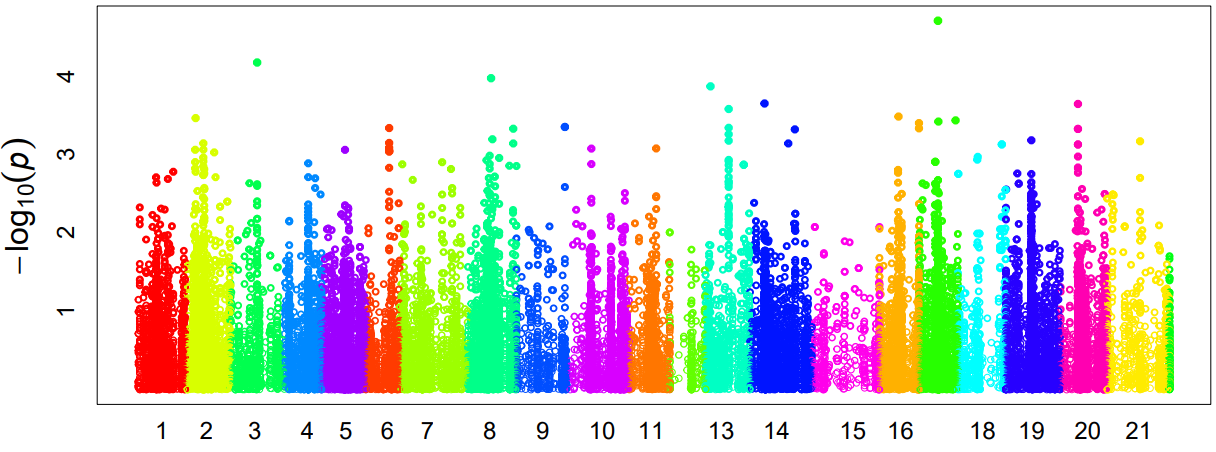 | 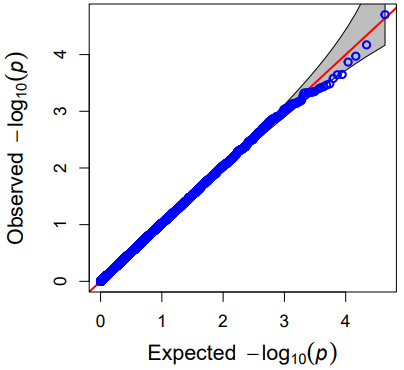 |
|  |  |
|  |  |
|  |  |
| Spike weight |  |
| 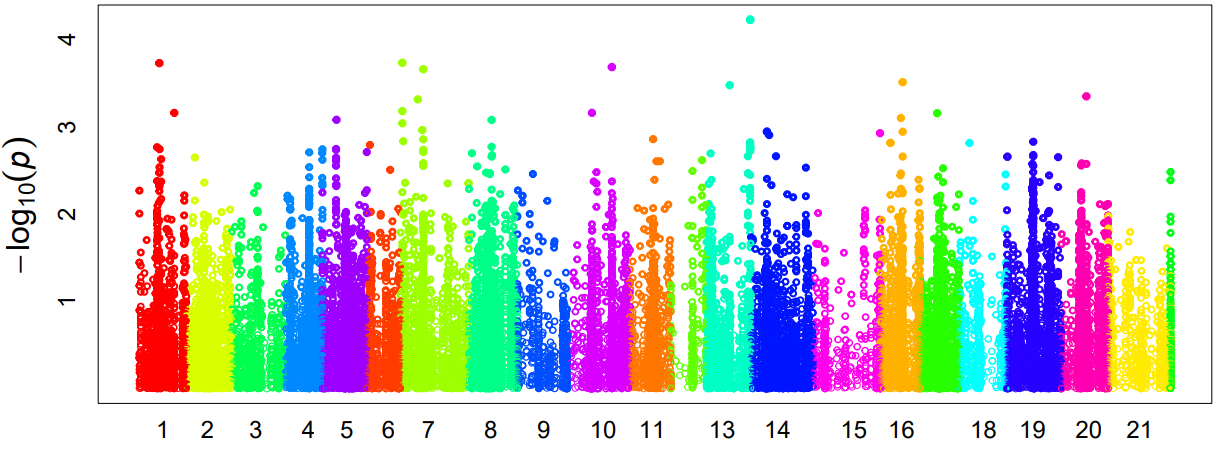 | 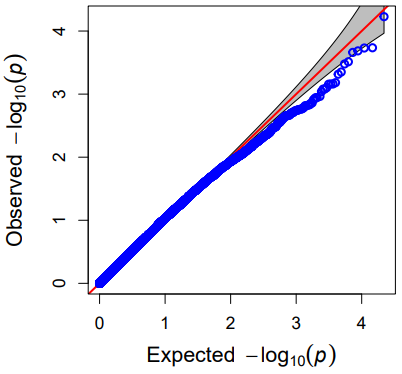 |
| Spike area |  |
| 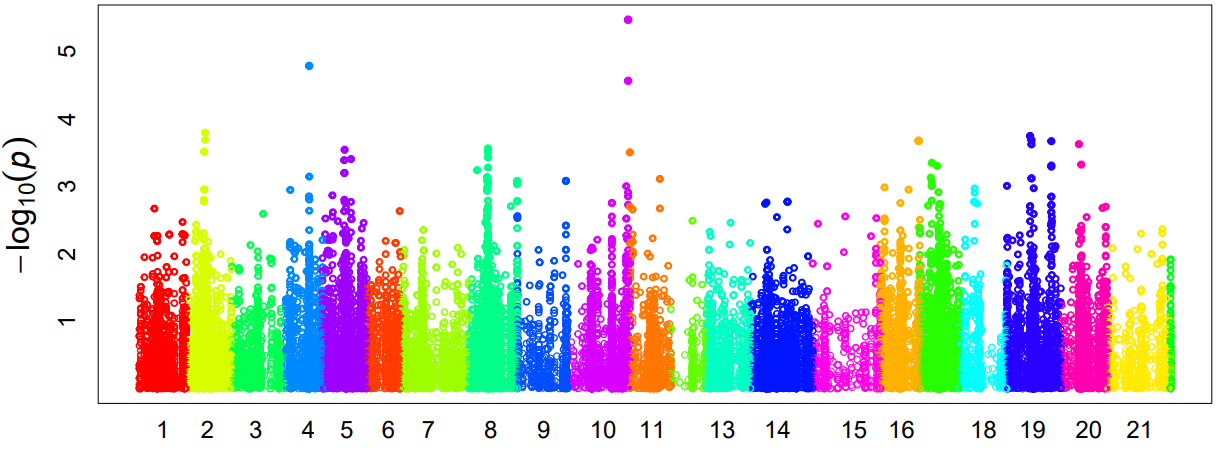 | 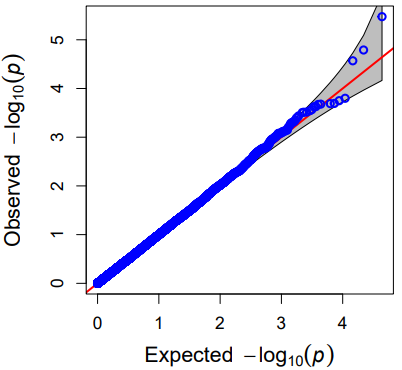 |
| Spike harvest index |  |
| 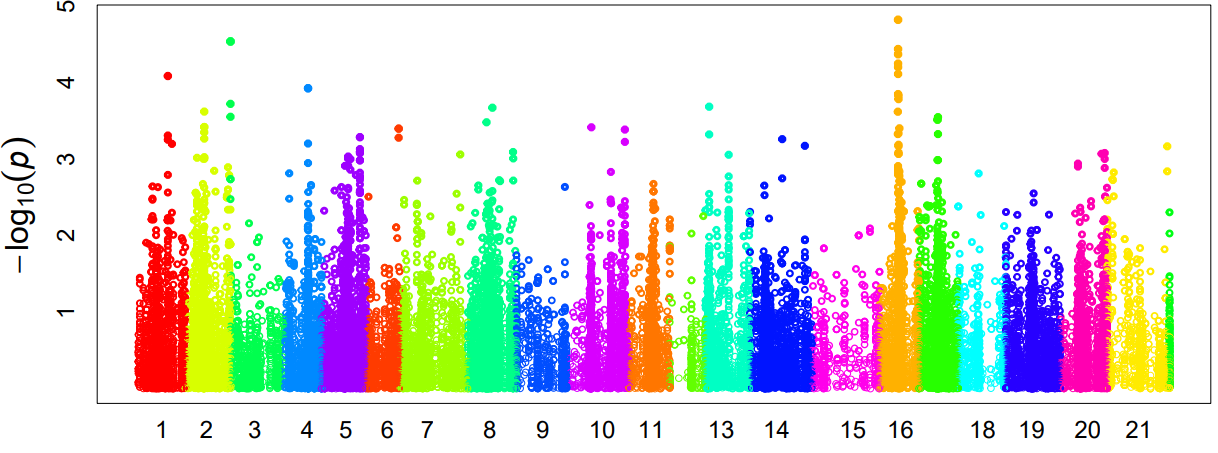 | 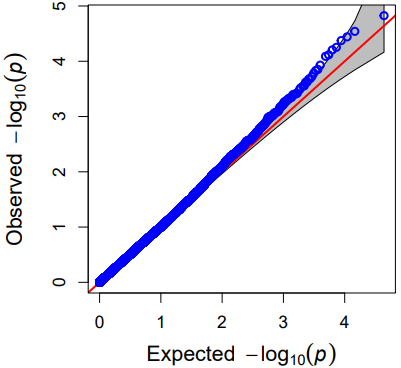 |
| Spike fertility |  |
| 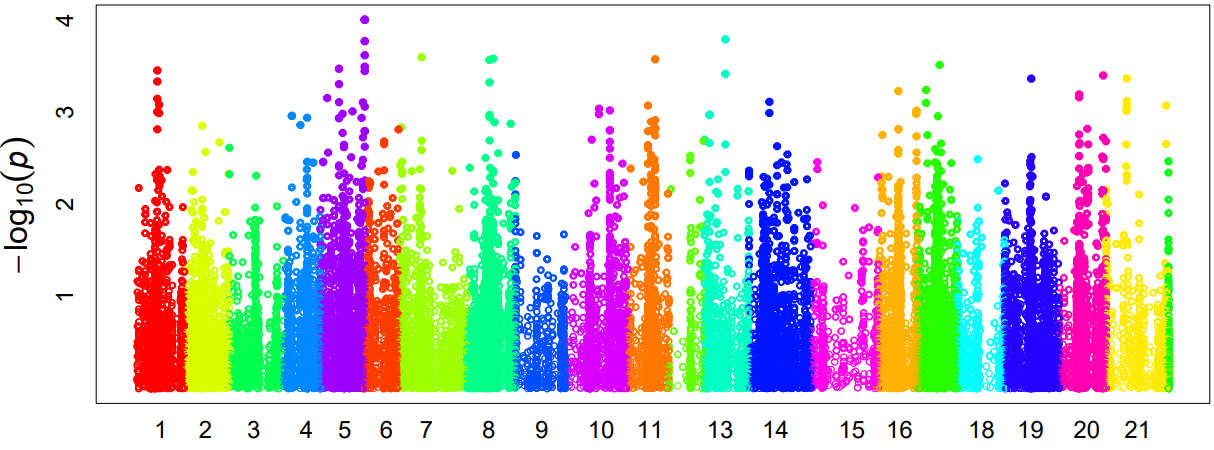 | 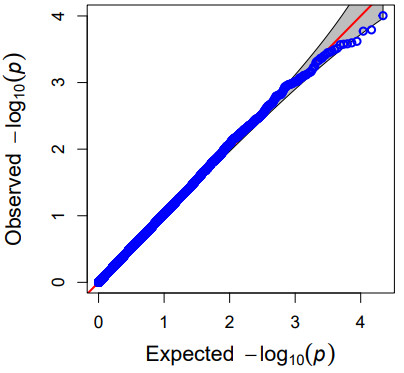 |
| **gBLUP (C)** |  |
| Plant height |  |
| 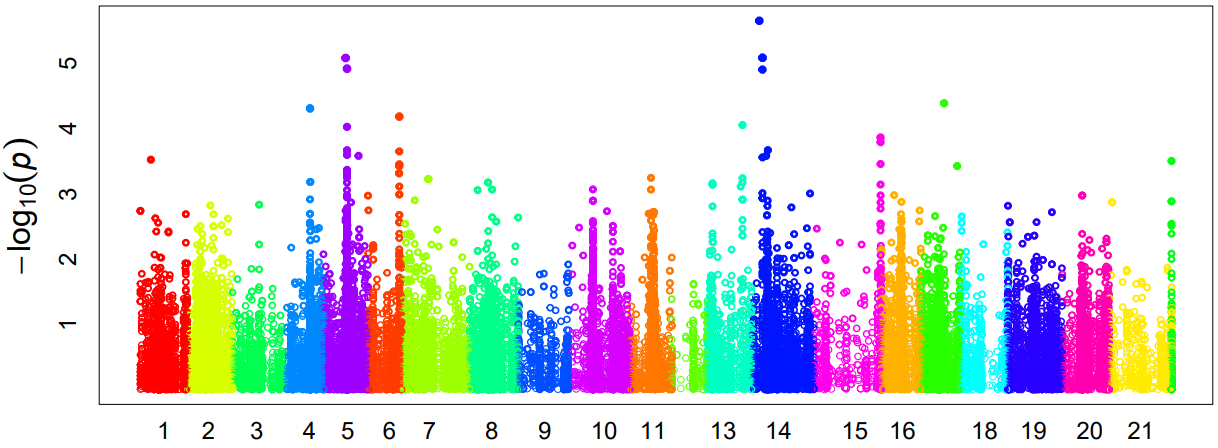 | 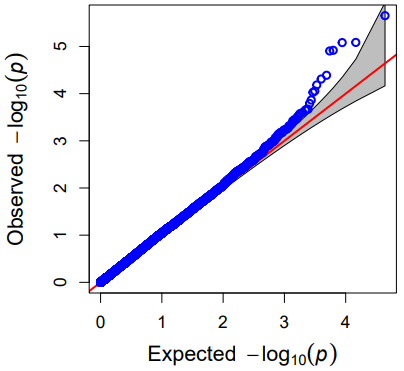 |
| Grain yield |  |
| 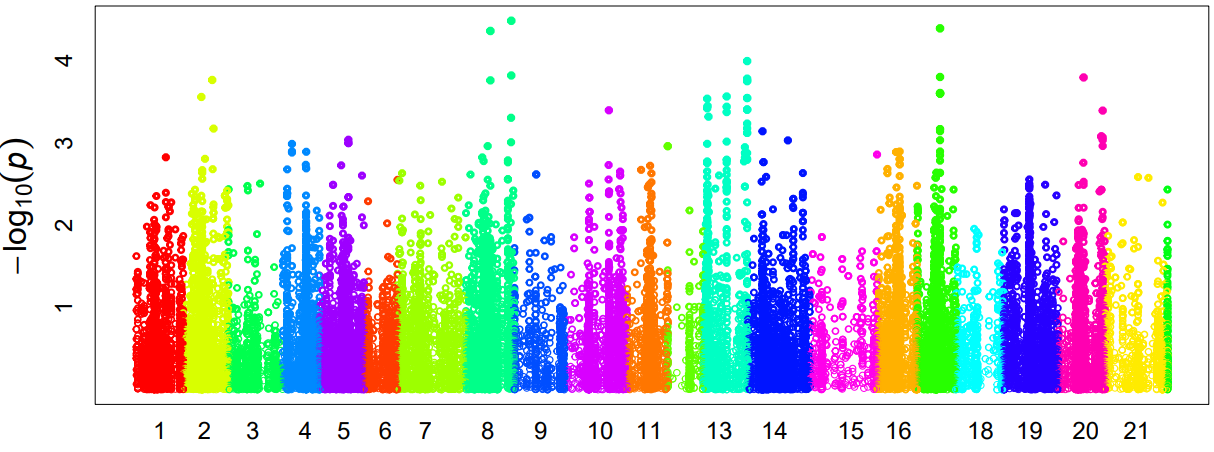 | 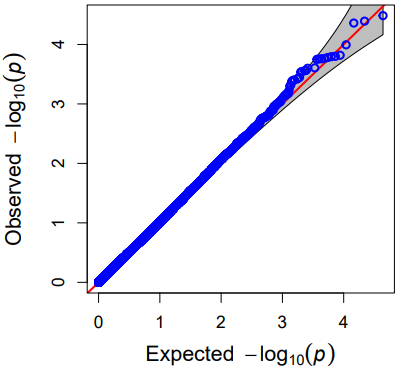 |
| Grain per spike |  |
| 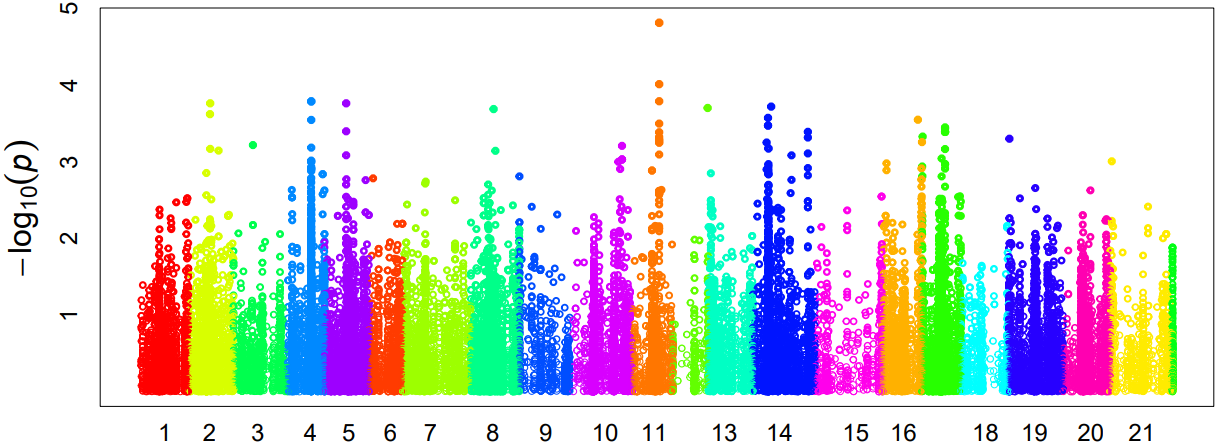 | 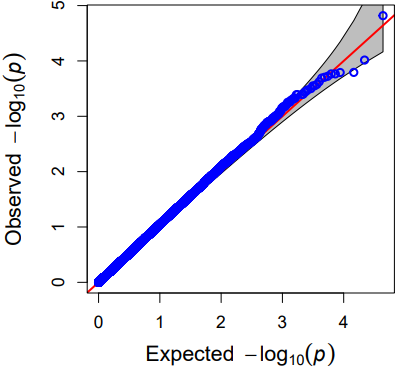 |
| Thousand kernel weight |  |
| 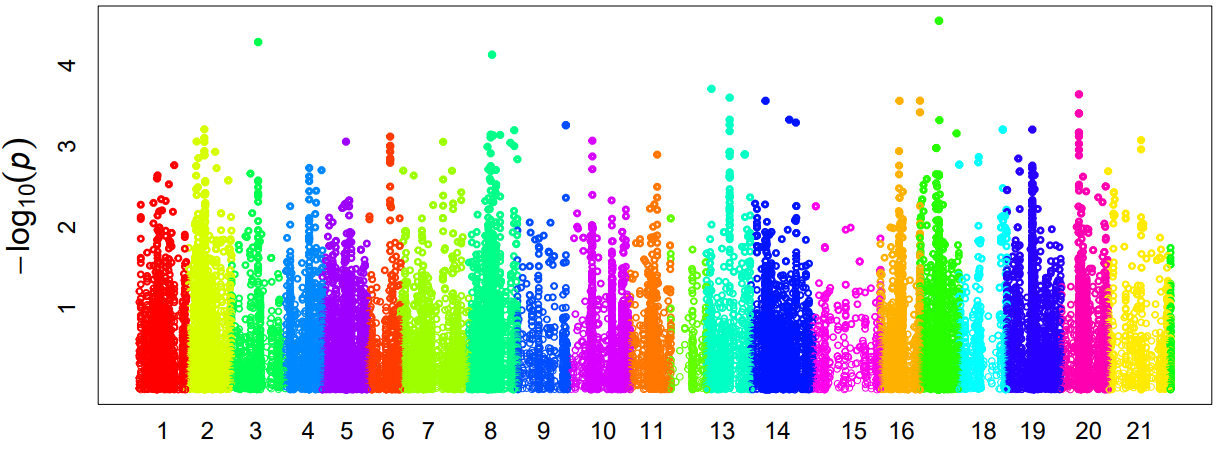 | 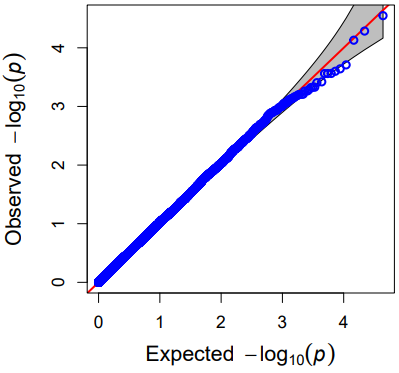 |
|  |  |
|  |  |
|  |  |
| Spike weight |  |
| 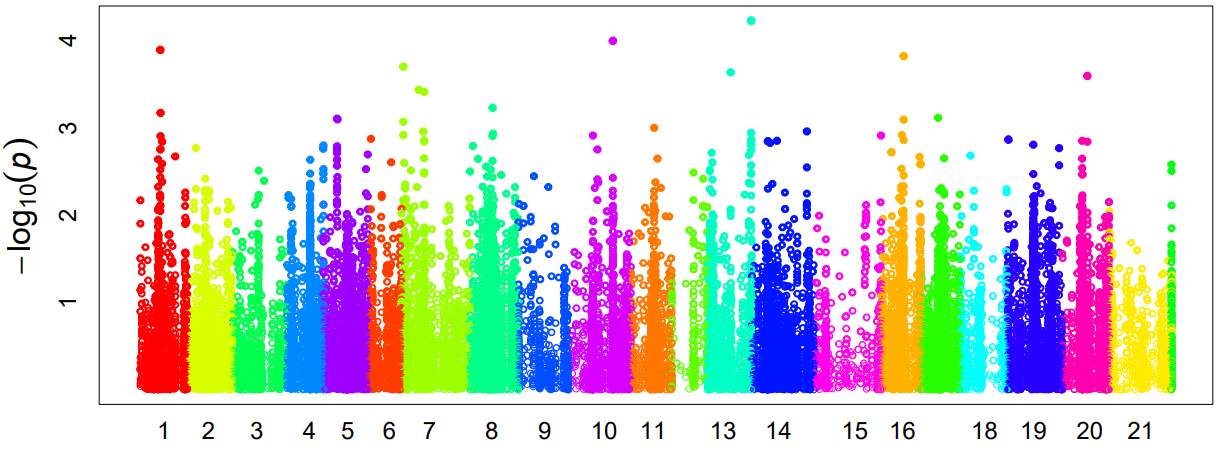 | 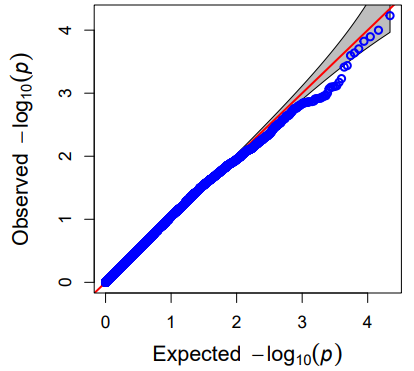 |
| Spike area |  |
| 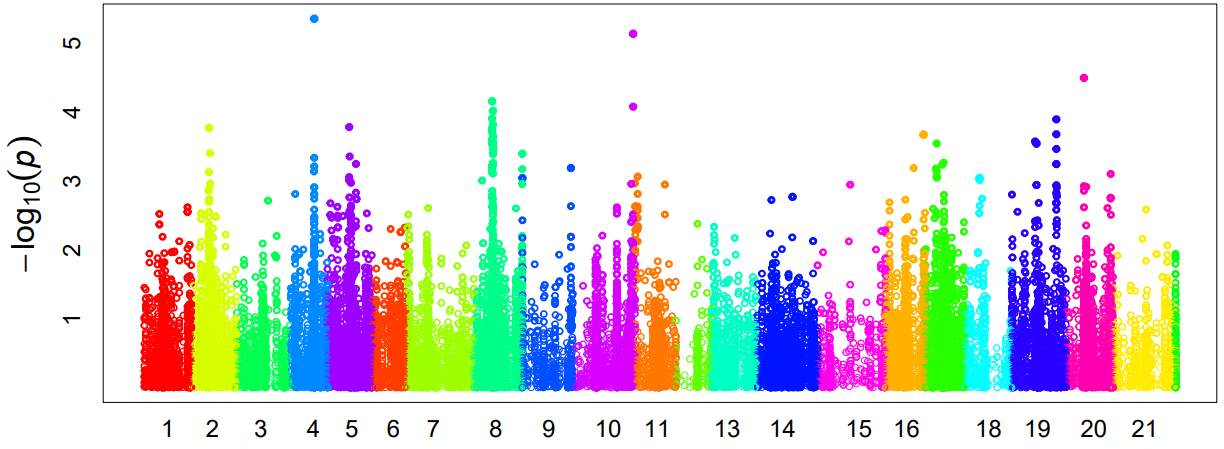 | 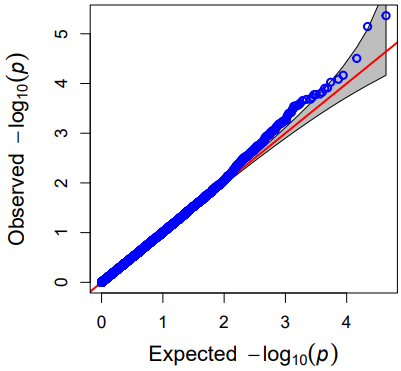 |
| Spike harvest index |  |
| 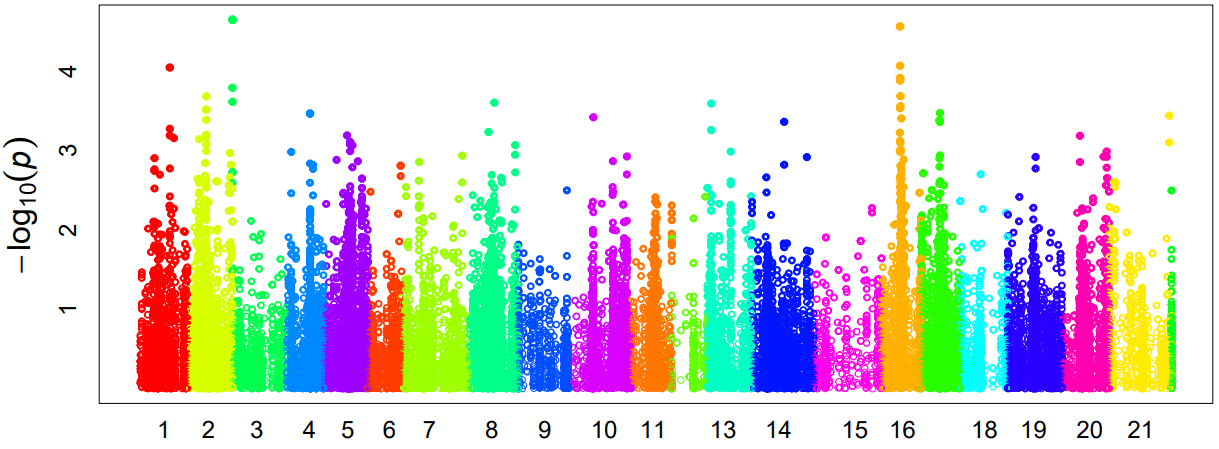 | 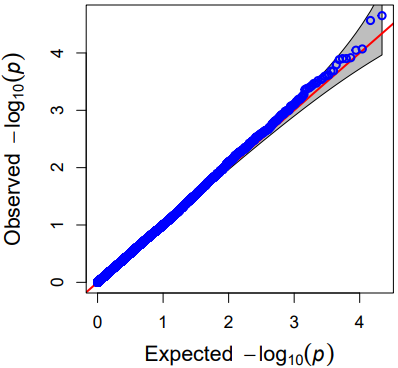 |
| Spike fertility |  |
| 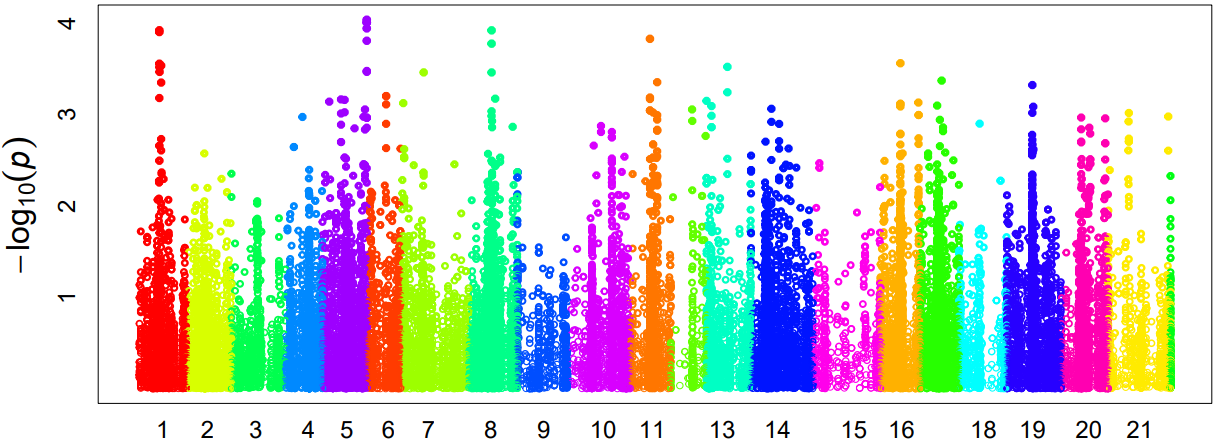 | 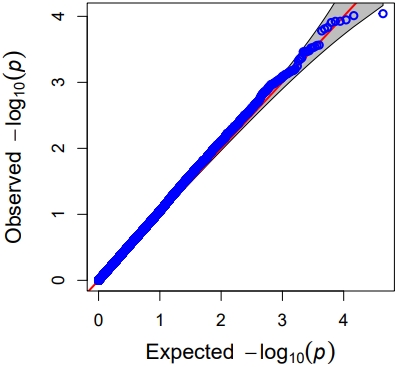 |
|  |  |
|  |  |
|  |  |
|  |  |
| **rrBLUP (D)** |  |
| Plant height |  |
| 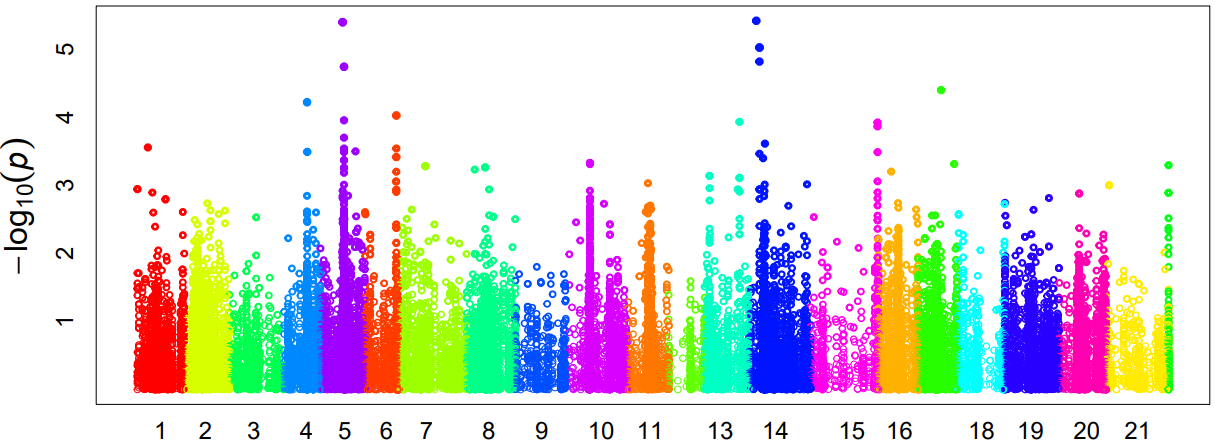 | 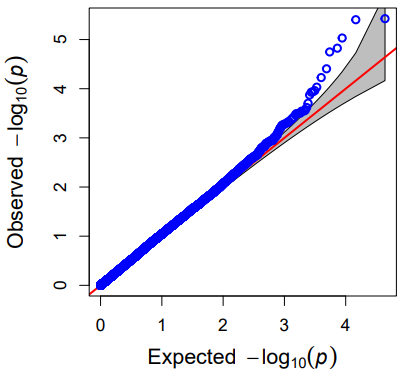 |
| Grain yield |  |
| 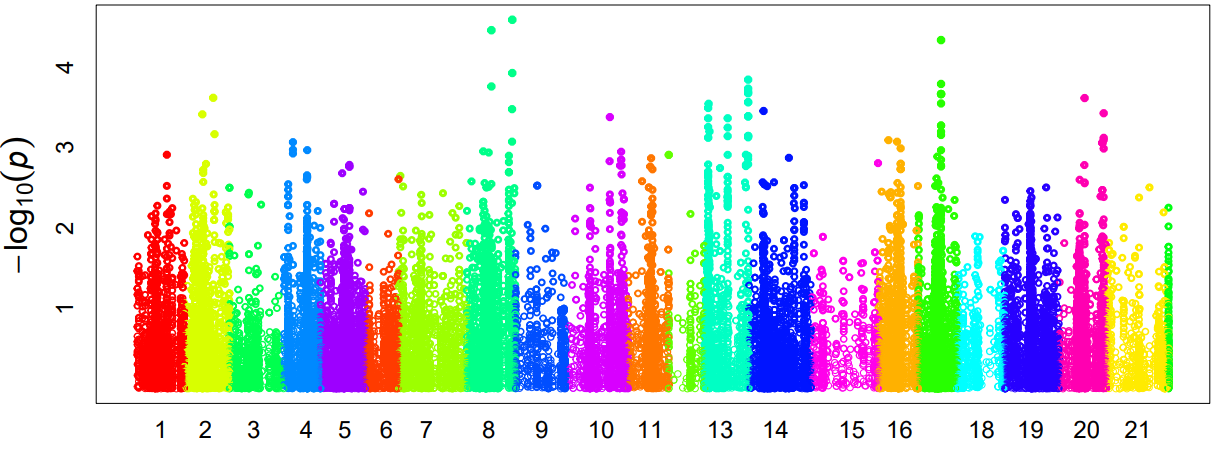 | 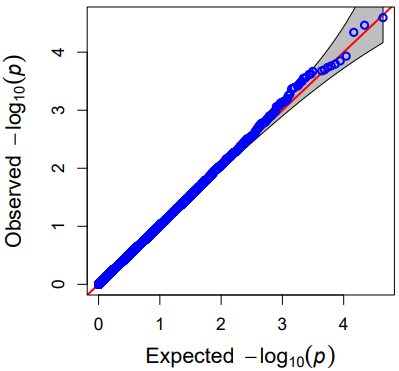 |
| Grain per spike |  |
| 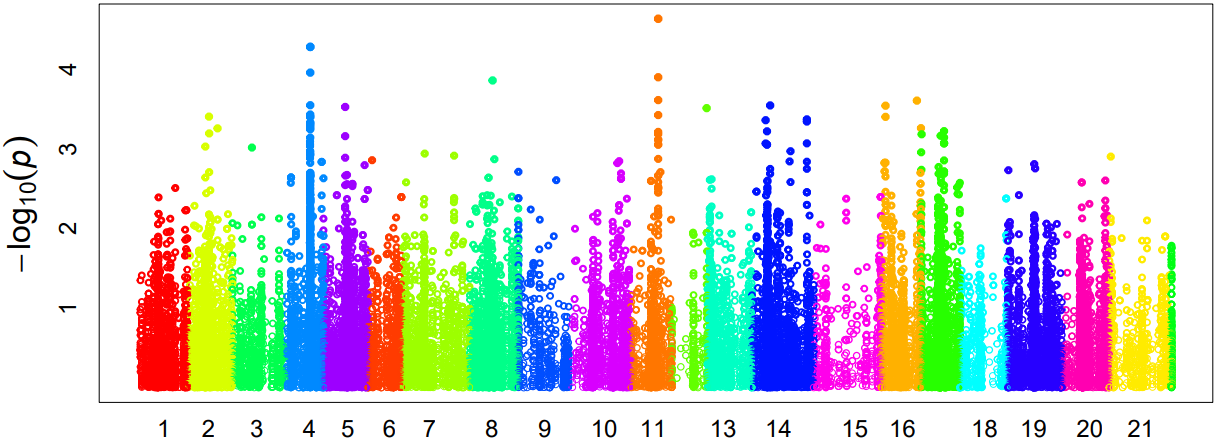 | 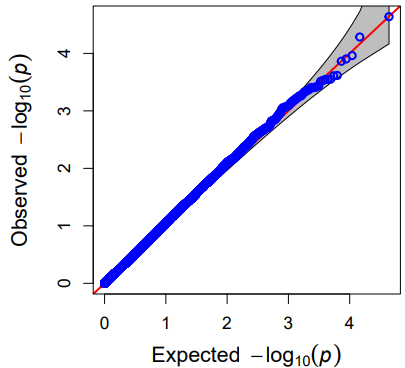 |
| Thousand kernel weight |  |
| 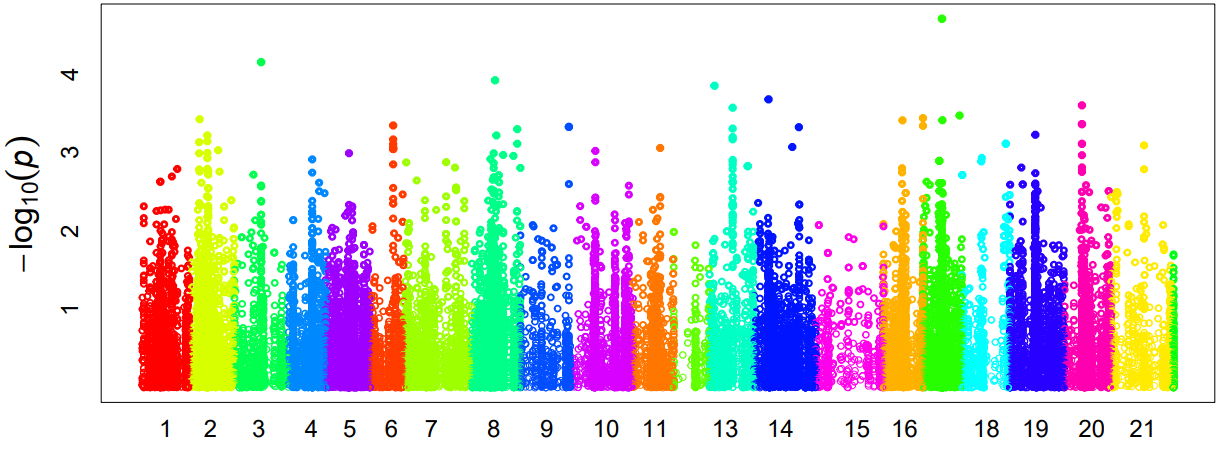 | 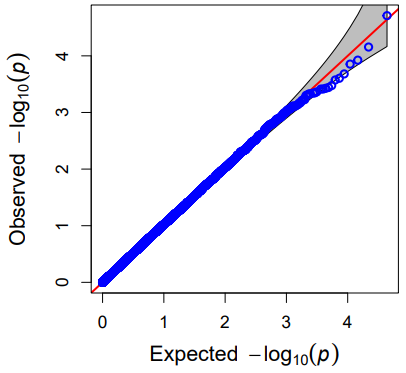 |
|  |  |
|  |  |
|  |  |
| Spike weight |  |
| 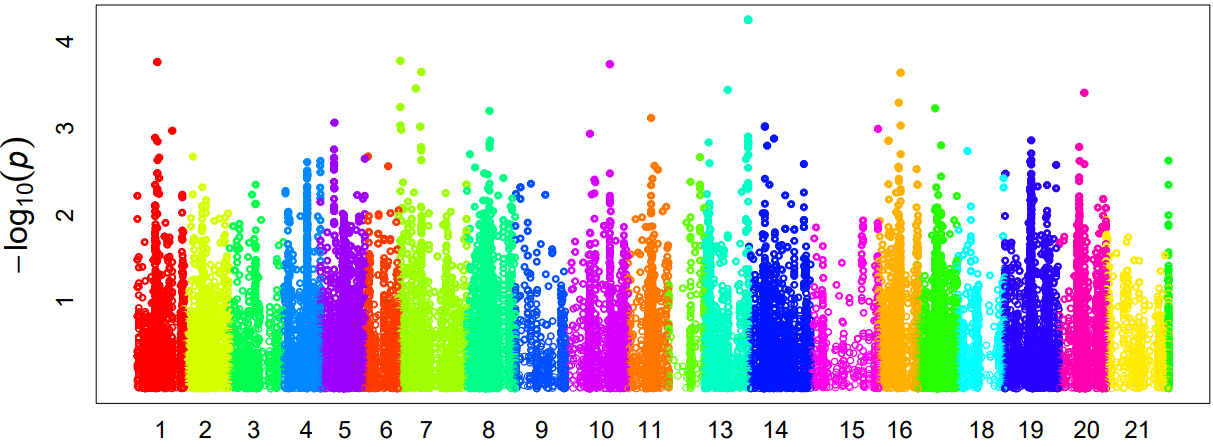 | 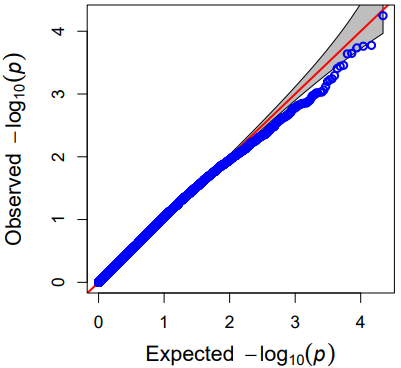 |
| Spike area |  |
| 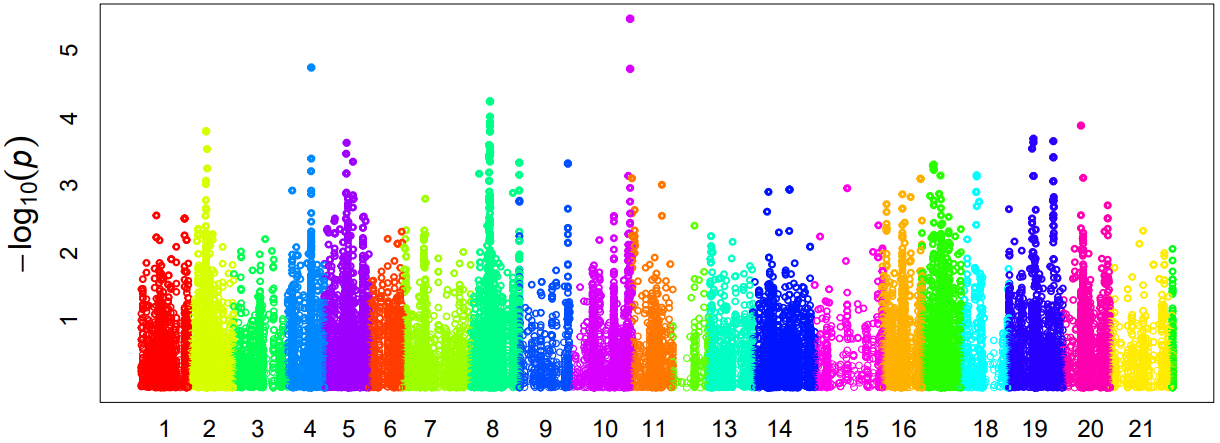 | 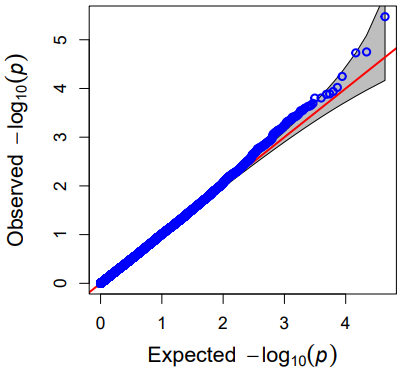 |
| Spike harvest index |  |
| 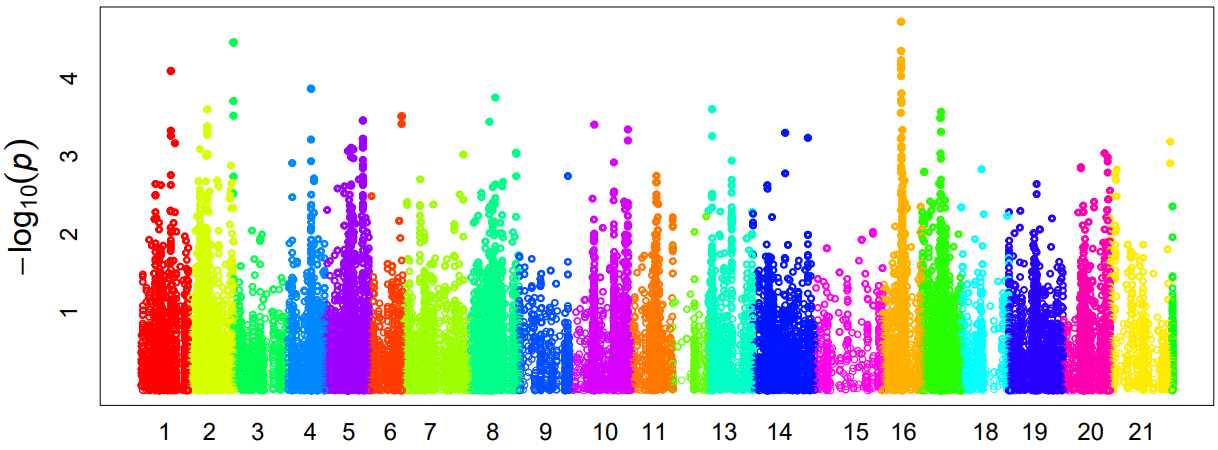 | 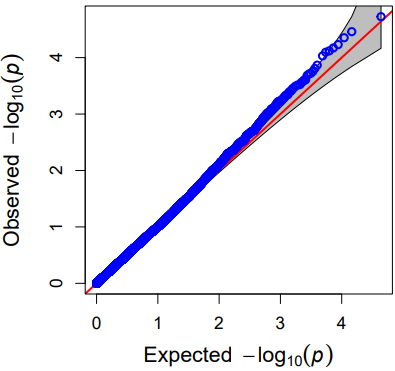 |
| Spike fertility |  |
| 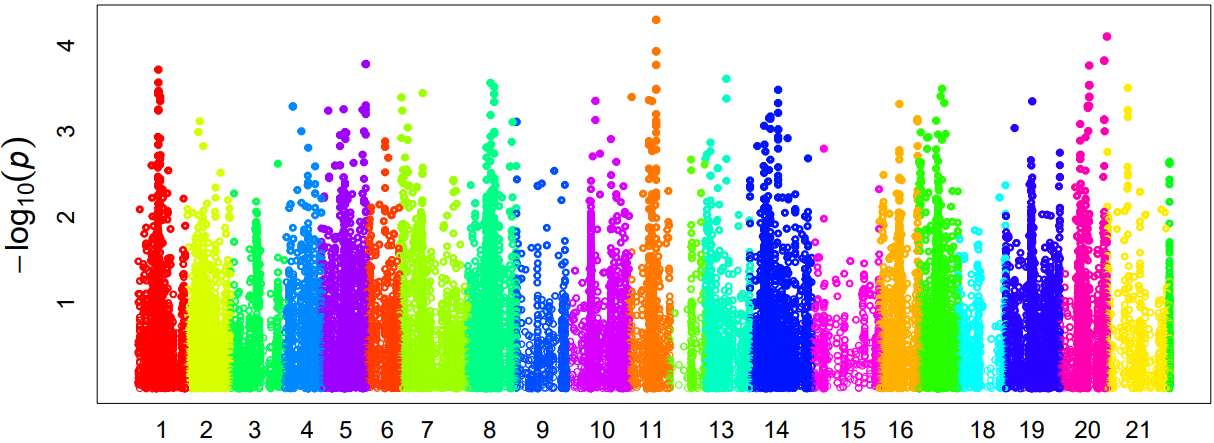 | 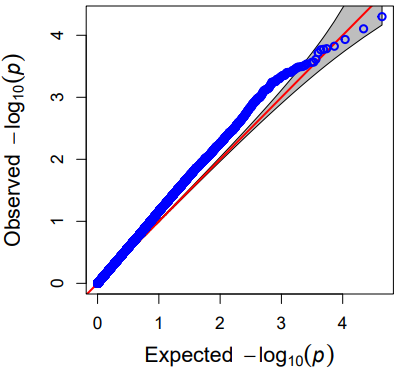 |

**Supplementary Fig. 3** Manhattan and QQ-plots of highly associated haplotypes for and MLM in Iranian wheat landraces and cultivars in well-watered environments. X axis represents chromosomes: 1) 1A, 2) 1B, 3) 1D, 4) 2A, 5) 2B, 6) 2D, 7) 3A, 8) 3B, 9) 3D, 10) 4A, 11) 4B, 12) 4D, 13) 5A, 14) 5B, 15) 5D, 16) 6A, 17) 6B, 18) 6D, 19) 7A, 20) 7B, 21)7D.

**Supplementary Fig. 4** GWAS results for agronomic traits and breeding Values of Iranian landraces and cultivars in rain-fed environments. Agronomic traits (A), BRR (B), gBLUP (C), and rrBLUP (D). Abbreviations: PH, Plant height (cm); GY, Grain yield (g per plant); GN, Grain number per spike; TKW, Thousand kernel weight (g); SW, Spike weight (g); SA, Spike area (cm^2^); SH, Spike harvest index (%); SF, Spike fertility.

| **Agronomic traits (A)** |  |
| --- | --- |
| Plant height |  |
| 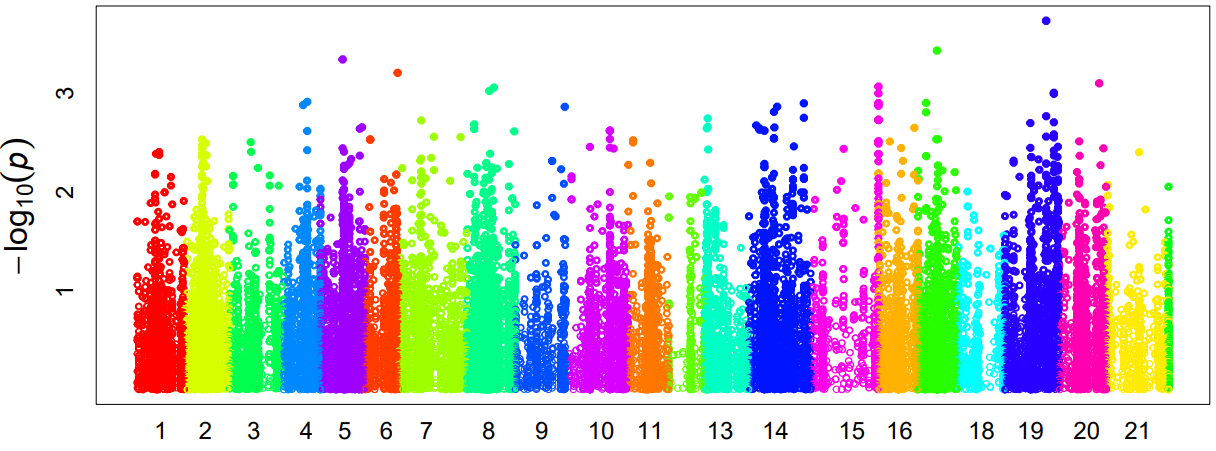 | 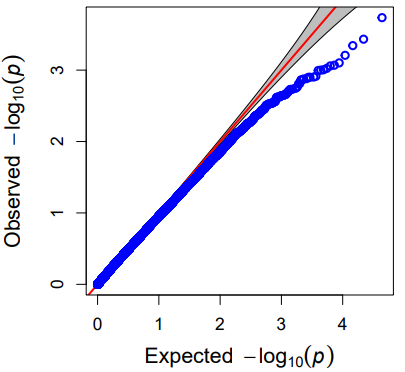 |
| Grain yield |  |
| 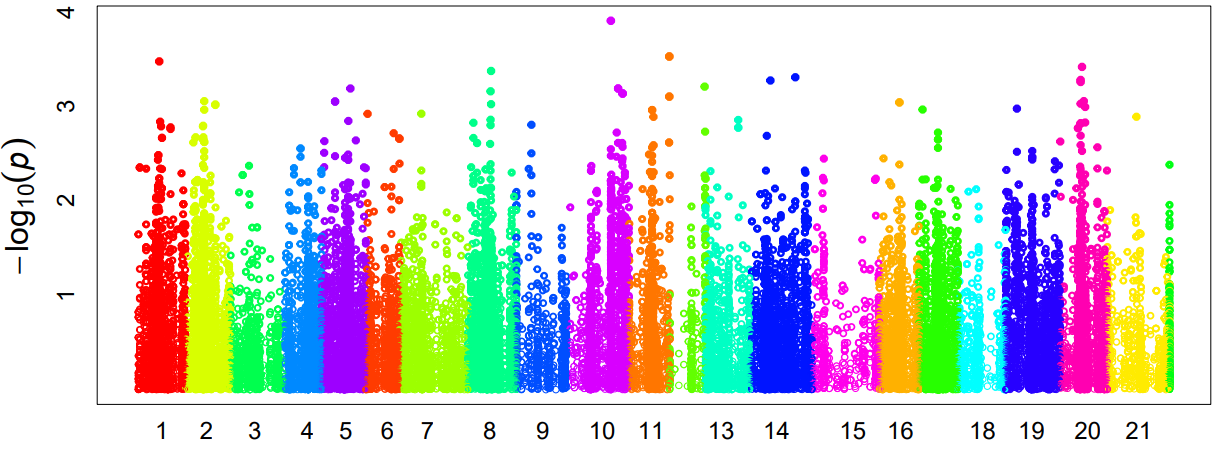 | 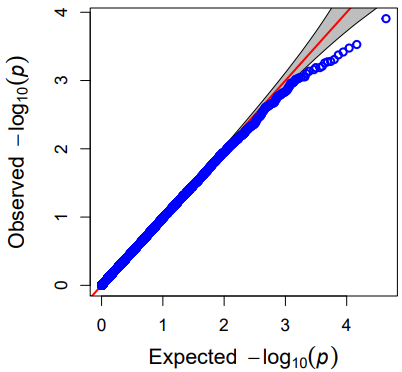 |
| Grain per spike |  |
| 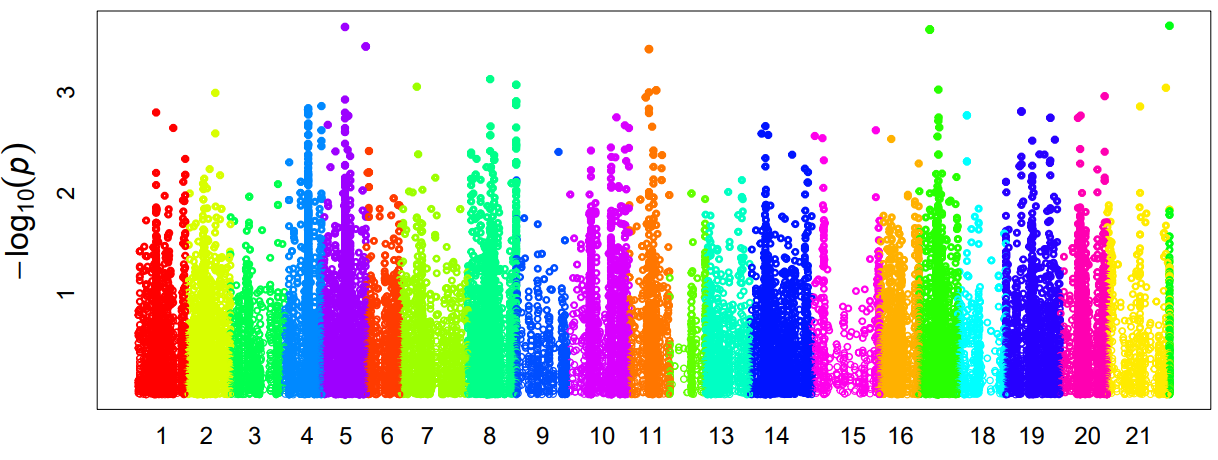 | 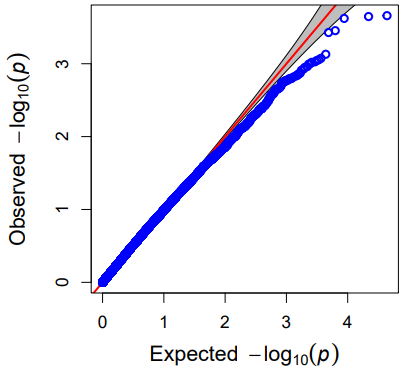 |
| Thousand kernel weight |  |
| 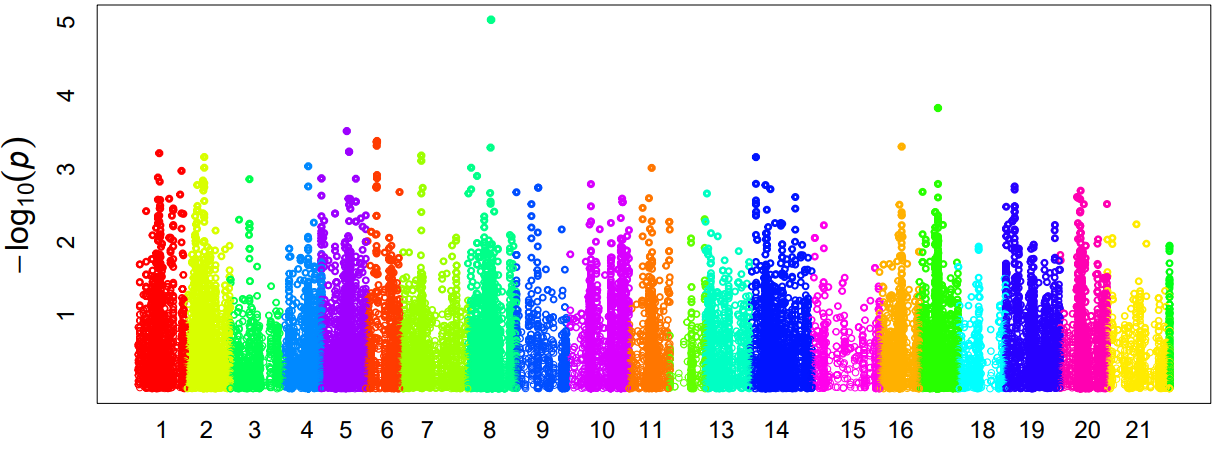 | 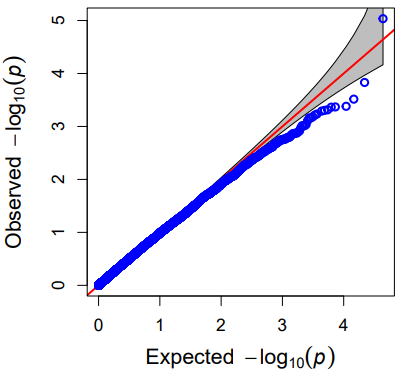 |
|  |  |
|  |  |
|  |  |
| Spike weight |  |
| 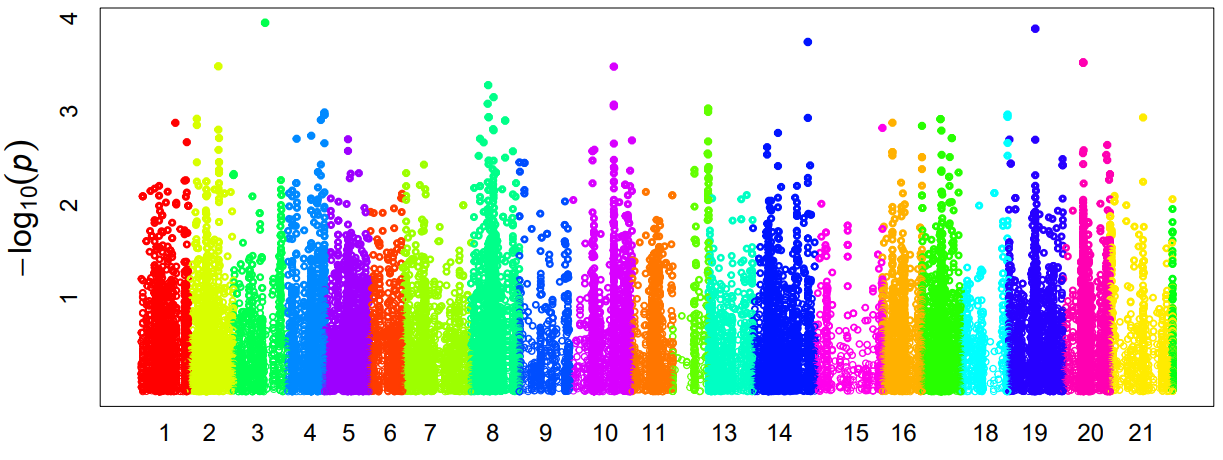 | 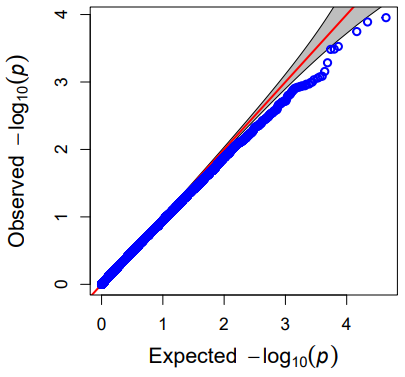 |
| Spike area |  |
| 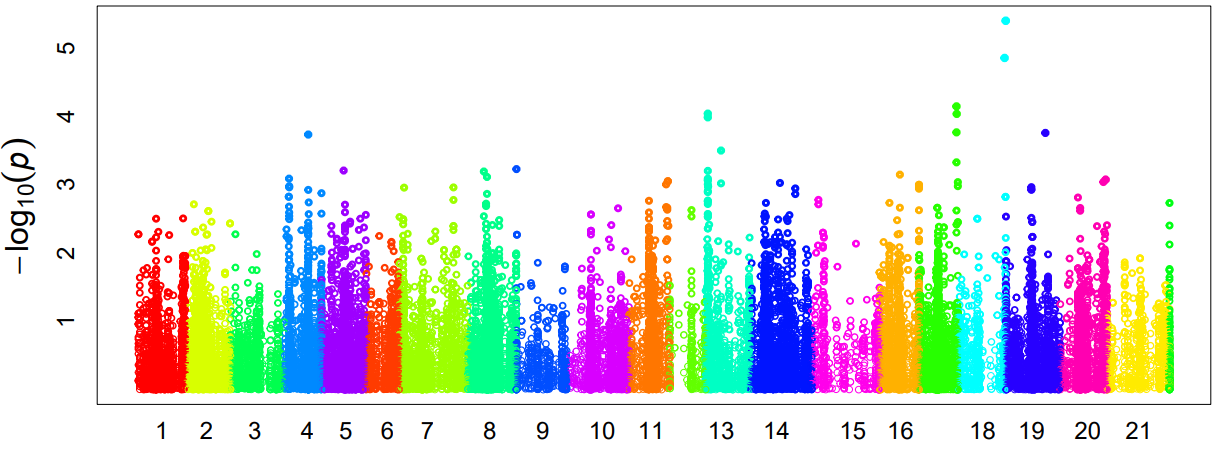 | 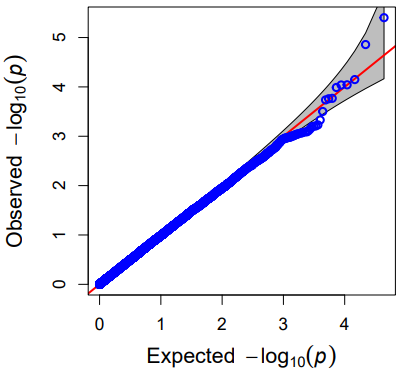 |
| Spike harvest index |  |
| 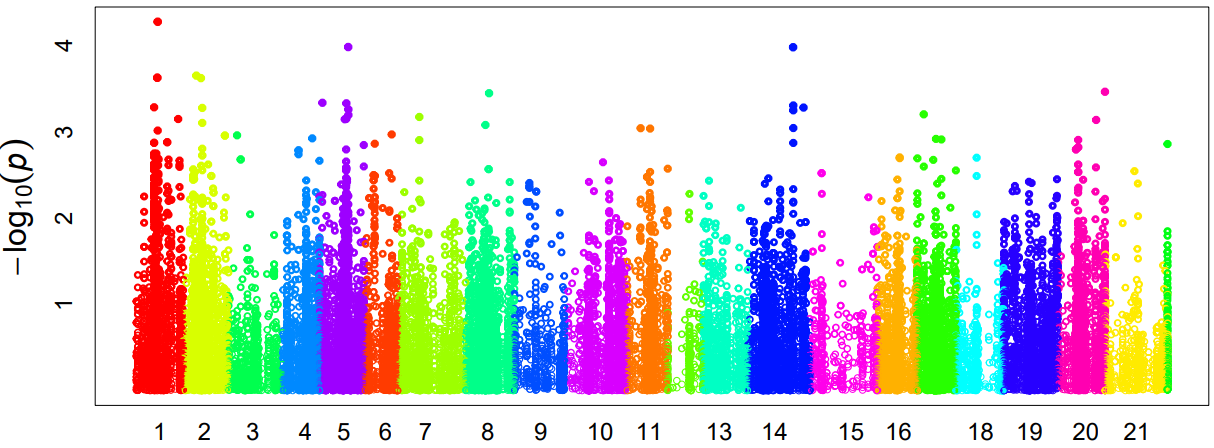 | 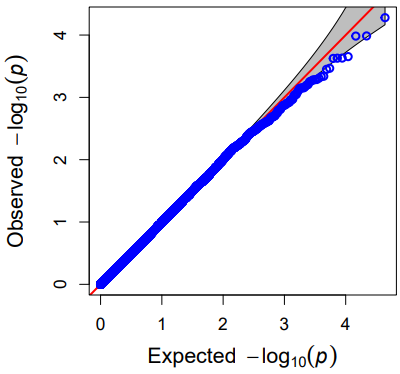 |
| Spike fertility |  |
| 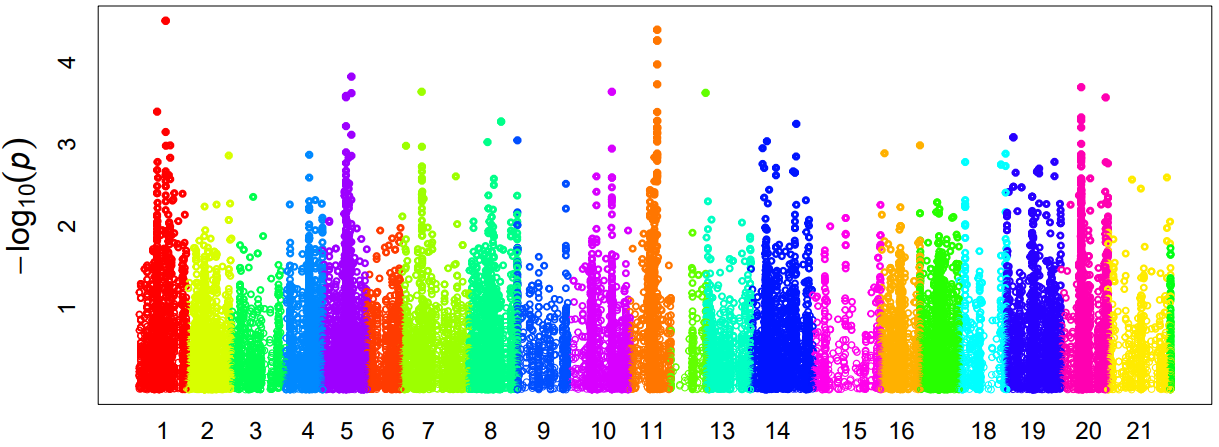 | 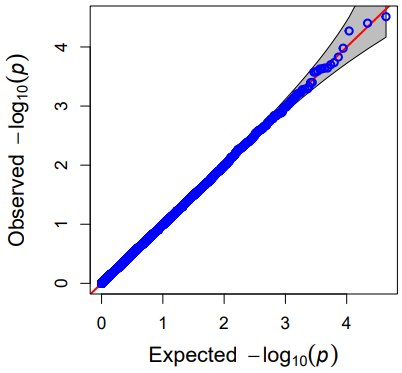 |
|  |  |
|  |  |
|  |  |
|  |  |
|  |  |
| **BRR (B)** |  |
| Plant height |  |
| 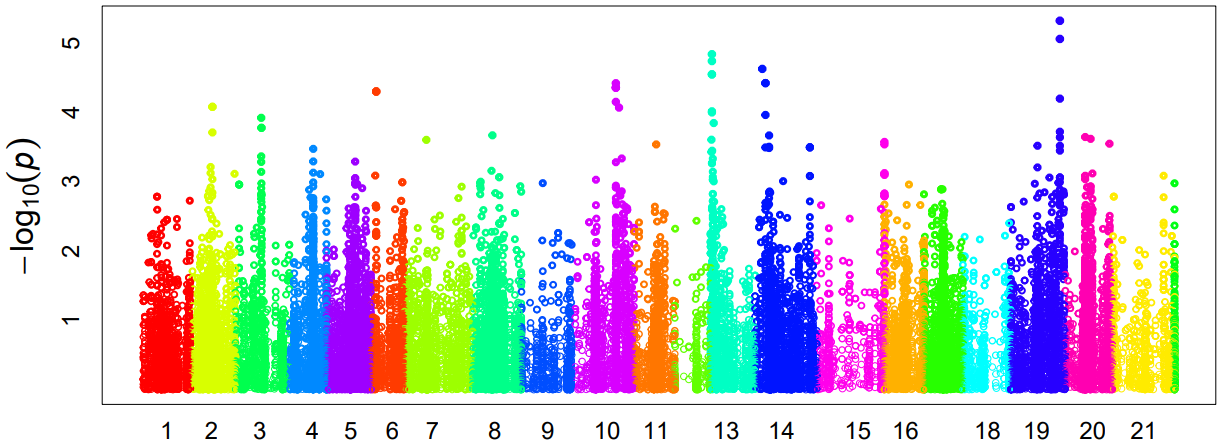 | 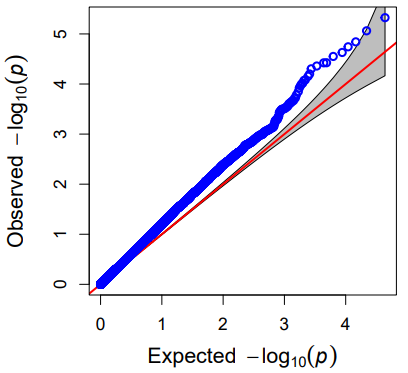 |
| Grain yield |  |
| 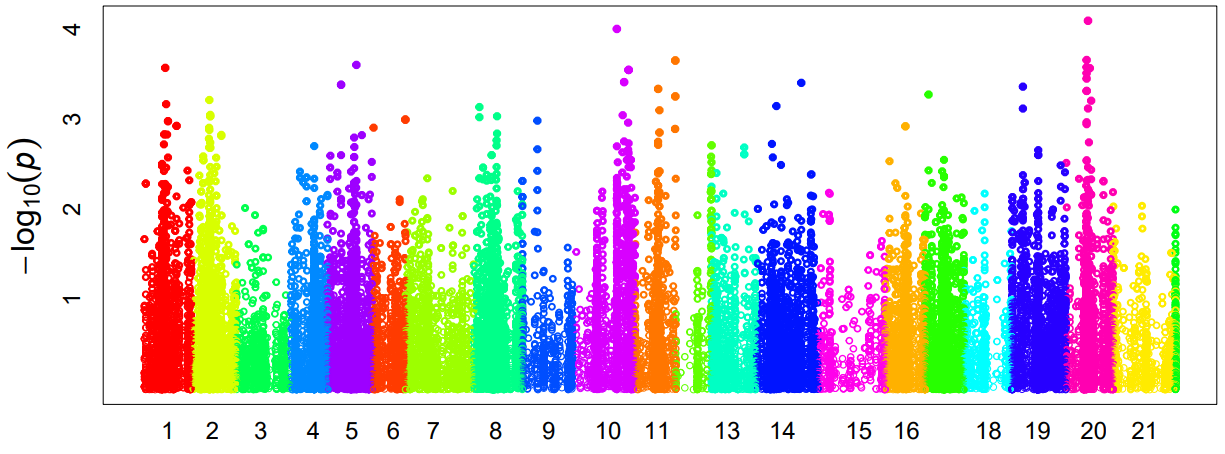 | 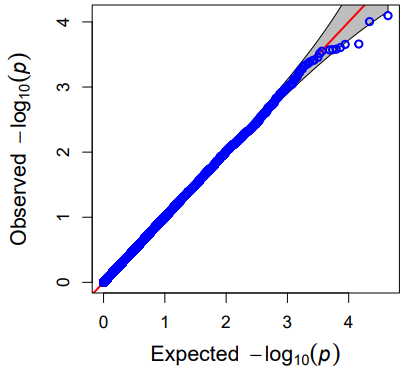 |
| Grain per spike |  |
| 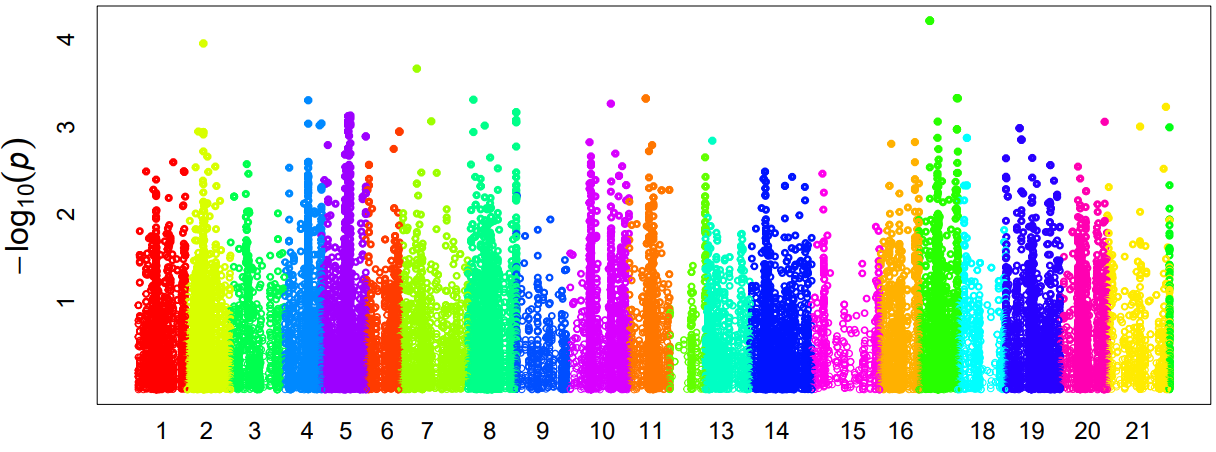 | 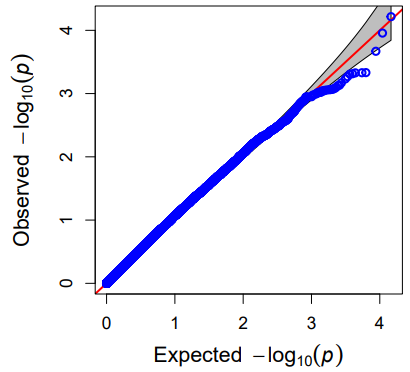 |
| Thousand kernel weight |  |
| 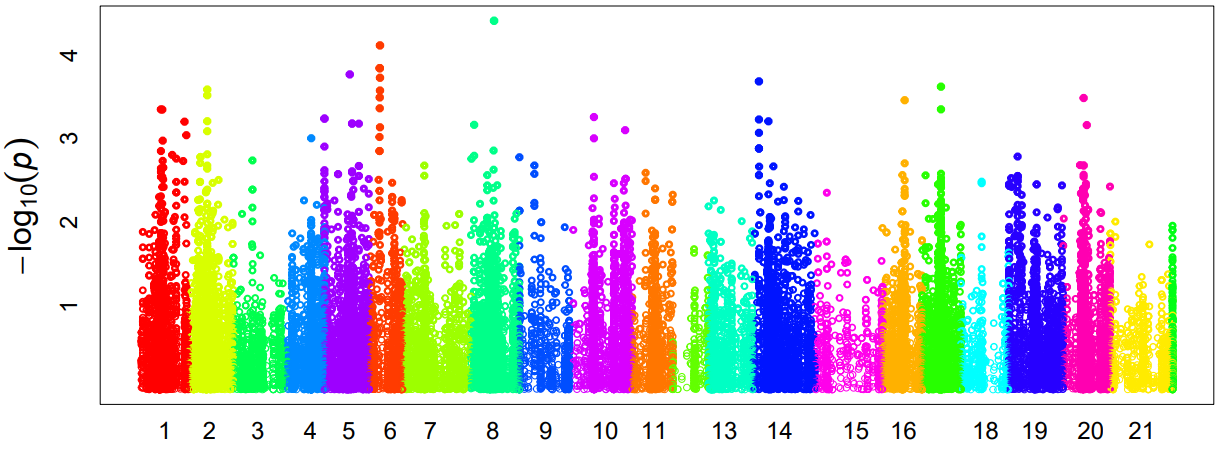 | 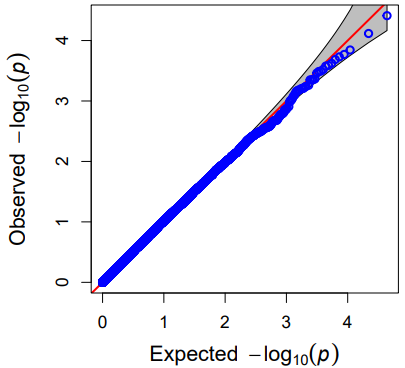 |
|  |  |
|  |  |
|  |  |
| Spike weight |  |
| 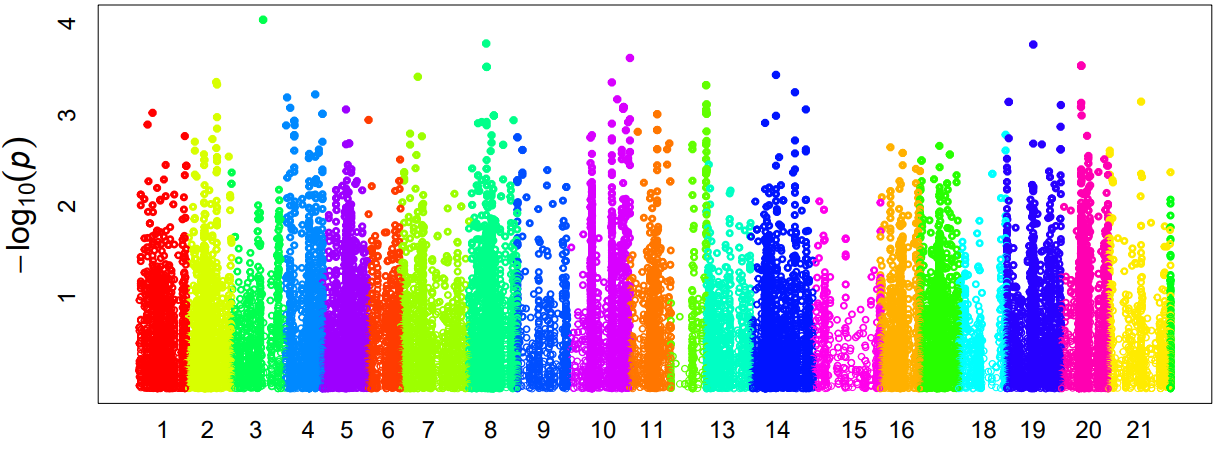 | 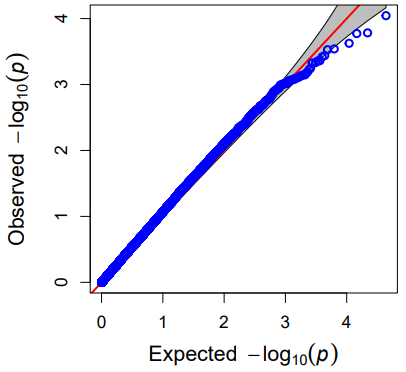 |
| Spike area |  |
| 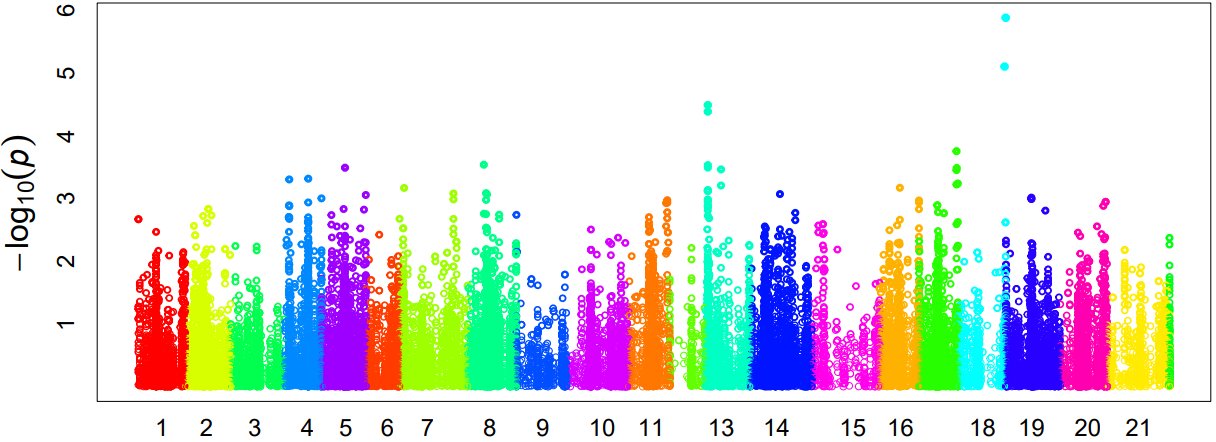 | 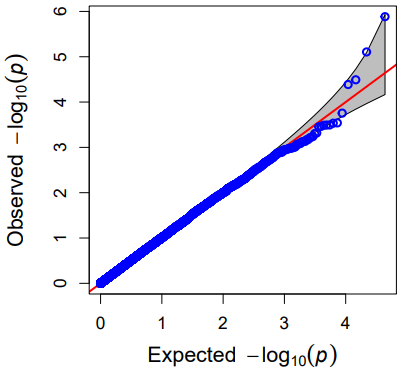 |
| Spike harvest index |  |
| 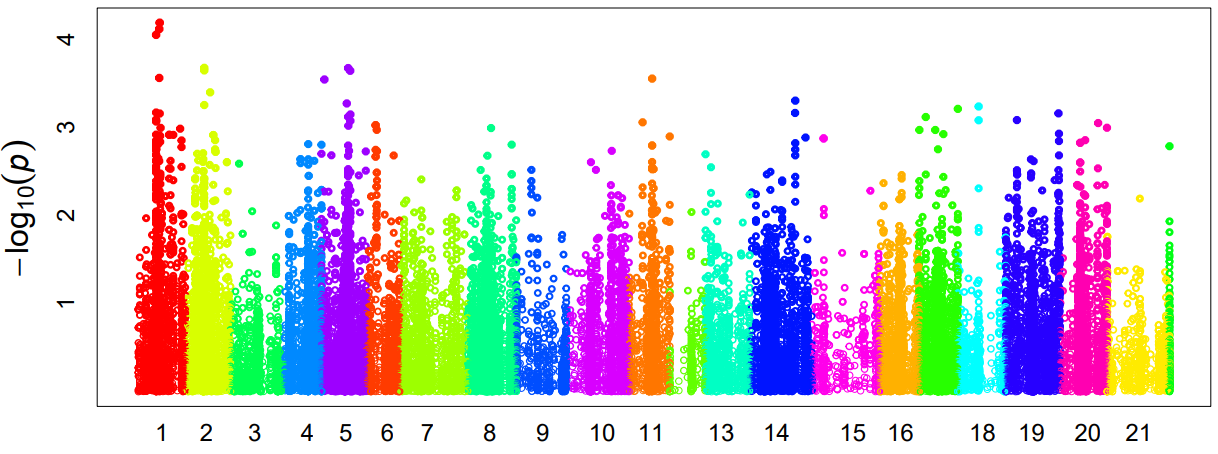 | 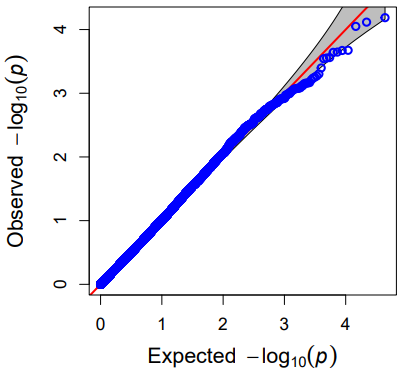 |
| Spike fertility |  |
| 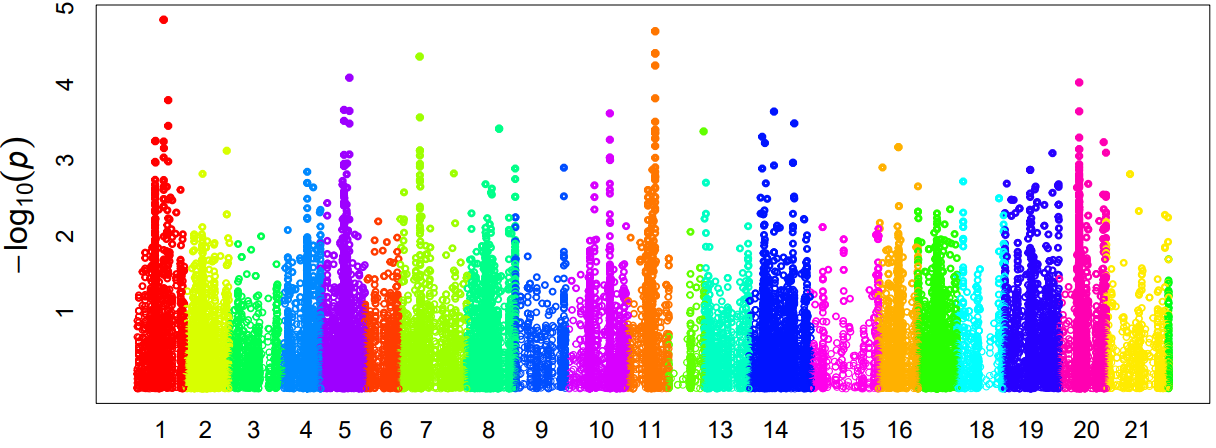 | 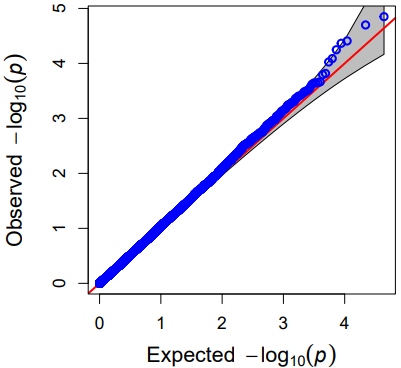 |
|  |  |
| **gBLUP (C)** |  |
| Plant height |  |
| 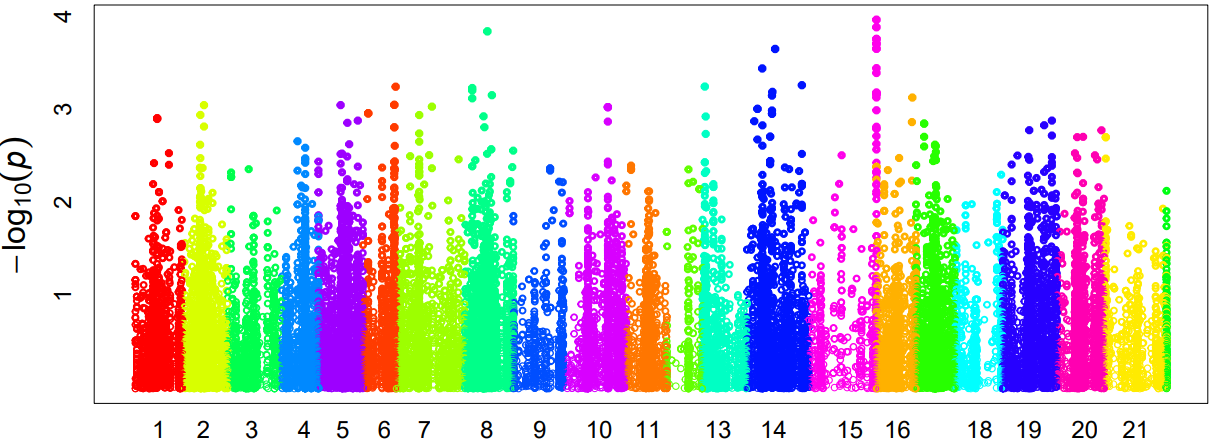 | 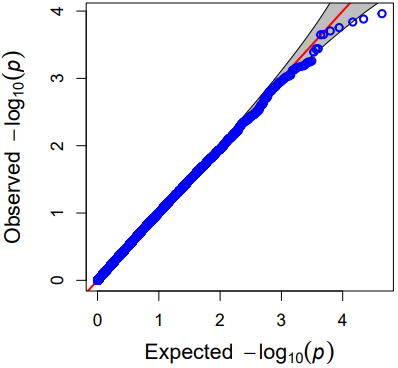 |
| Grain yield |  |
|  |  |
| Grain per spike |  |
|  |  |
| Thousand kernel weight |  |
|  |  |
|  |  |
|  |  |
|  |  |
| Spike weight |  |
|  |  |
| Spike area |  |
|  |  |
| Spike harvest index |  |
|  |  |
| Spike fertility |  |
|  |  |
|  |  |
|  |  |
|  |  |
|  |  |
| **rrBLUP (D)** |  |
| Plant height |  |
|  |  |
| Grain yield |  |
|  |  |
| Grain per spike |  |
|  |  |
| Thousand kernel weight |  |
|  |  |
|  |  |
|  |  |
|  |  |
| Spike weight |  |
|  |  |
| Spike area |  |
|  |  |
| Spike harvest index |  |
|  |  |
| Spike fertility |  |
|  |  |

**Supplementary Fig. 5** Manhattan and QQ-plots of highly associated haplotypes for and MLM in Iranian wheat landraces and cultivars in rain-fed environments. X axis represents chromosomes: 1) 1A, 2) 1B, 3) 1D, 4) 2A, 5) 2B, 6) 2D, 7) 3A, 8) 3B, 9) 3D, 10) 4A, 11) 4B, 12) 4D, 13) 5A, 14) 5B, 15) 5D, 16) 6A, 17) 6B, 18) 6D, 19) 7A, 20) 7B, 21)7D.

**Supplementary Fig 6.** The KEGG pathway of metabolic pathways.

**Supplementary Fig 7.** The KEGG pathway of oxidative phosphorylation.

**Supplementary Fig 8.** The KEGG pathway of biosynthesis of amino acids.

**Supplementary Fig 9.** The KEGG pathway of ascorbate and aldarate metabolism

**Supplementary Fig 10.** The KEGG pathway of sulfur metabolism.

**Supplementary Fig 11.** The KEGG pathway of fatty acid elongation.

The pathway map without coloring is the original version that is manually drawn by in-house software called KegSketch. The other pathway maps with coloring are all computationally generated as summarized below.

Reference pathway: this is the original version; white boxes are hyperlinked to KO, ENZYME, and REACTION entries in metabolic pathways; they are hyperlinked to KO entries in non-metabolic pathways.

Reference pathway (KO): blue boxes are hyperlinked to KO entries that are selected from the original version.

Reference pathway (EC): blue boxes are hyperlinked to ENZYME entries that are selected from the original version.

Reference pathway (Reaction): blue boxes are hyperlinked to REACTION entries that are selected from the original version.

Organism-specific pathway: green boxes are hyperlinked to GENES entries by converting K numbers (KO identifiers) to gene identifiers in the reference pathway, indicating the presence of genes in the genome and also the completeness of the pathway.
